# Four New Sesquiterpene Pyridine Alkaloids from the Roots of *Tripterygium wilfordii* Hook. f.

**DOI:** 10.3390/molecules31020271

**Published:** 2026-01-13

**Authors:** Ya-Dan Wang, Yu-Tong Li, Yong-Jian Wang, Zhong-Mou Zhang, Bo-Rui Zou, Ying-Lin Dai, Hui-Ying Yang, Xian-Fu Wu

**Affiliations:** 1 National Institutes for Food and Drug Control, Beijing 102629, China; y.dwang@163.com (Y.-D.W.); liyutongvincent@163.com (Y.-T.L.); 15651902092@163.com (Y.-L.D.);; 2 State Key Laboratory of Component-Based Chinese Medicine, Tianjin Key Laboratory of TCM Chemistry and Analysis, Tianjin University of Traditional Chinese Medicine, Jinghai, Tianjin 301617, China; 3 School of Traditional Chinese Medicine, Beijing University of Chinese Medicine, Beijing 102629, China; 4 State Key Laboratory of Drug Regulatory Science, Beijing 102629, China

**Keywords:** *Tripterygium wilfordii*, sesquiterpene pyridine alkaloids (SPAs), anti-inflammatory

## Abstract

Investigation of the roots of *Tripterygium wilfordii* Hook. f. resulted in the isolation of nine sesquiterpene pyridine alkaloids (SPAs), including four previously undescribed compounds **1**–**4**. The structures of all compounds were elucidated by extensive spectroscopic analysis, including NMR and HRESIMS. In particular, compound **1** was found to possess an unprecedented cage-like ether moiety, representing the first report of such a structural feature within this class of alkaloids. All isolated compounds were evaluated for their anti-inflammatory activity using a lipopolysaccharide (LPS)-induced RAW264.7 macrophage model. Compounds **1**, **2**, and **4** exhibited significant inhibition of nitric oxide (NO) production, with IC_50_ values of 7.14 ± 1.89, 8.55 ± 0.37, and 14.76 ± 0.39 μM. Furthermore, compounds **1** and **2** suppressed the secretion of key pro-inflammatory cytokines, including TNF-*α*, IL-6, and IL-1*β*, in the same cellular model. These results not only enhanced the structural diversity of SPAs identified from *T. wilfordii*, but also highlight their potential as promising anti-inflammatory lead compounds.

## 1. Introduction

*Tripterygium wilfordii* Hook. f., a plant historically used in traditional Chinese medicine, is widely employed for treating rheumatoid arthritis, nephritis, and other autoimmune and inflammatory diseases. Modern pharmacological investigations have established that its bioactivities, including anti-inflammatory, immunosuppressive, and antitumor effects, are primarily attributed to a diverse array of secondary metabolites, such as diterpenoids, triterpenoids, and alkaloids. Among them, diterpenoids exhibit potent activities but are often associated with considerable toxicity, which limited their broader clinical application. In contrast, SPAs have attracted increasing research interest due to their unique structures, well-documented pharmacological profiles, and relatively lower toxicity. SPAs from *T. wilfordii* are characterized by a dihydro-*β*-agarofuran skeleton esterified with various nicotinic acid derivatives, leading to structurally diverse and highly substituted natural products. Based on the nature of the acylating groups, they are classified into subtypes such as wilfordate (W), evoninate (E), *iso*-wilfordate (IW), and *iso*-evoninate (IE) types [1,2,3,4]. Previous study reported that the alkaloids possess a range of pharmacological activities, including anti-inflammatory, immunomodulatory, and multidrug resistance-reversing effects, highlighting their potential as promising lead compounds for drug development [5,6,7,8,9]. Although numerous SPAs have been isolated from *T. wilfordii*, its chemical diversity and pharmacological potential remain incompletely explored.

**Figure 1:**
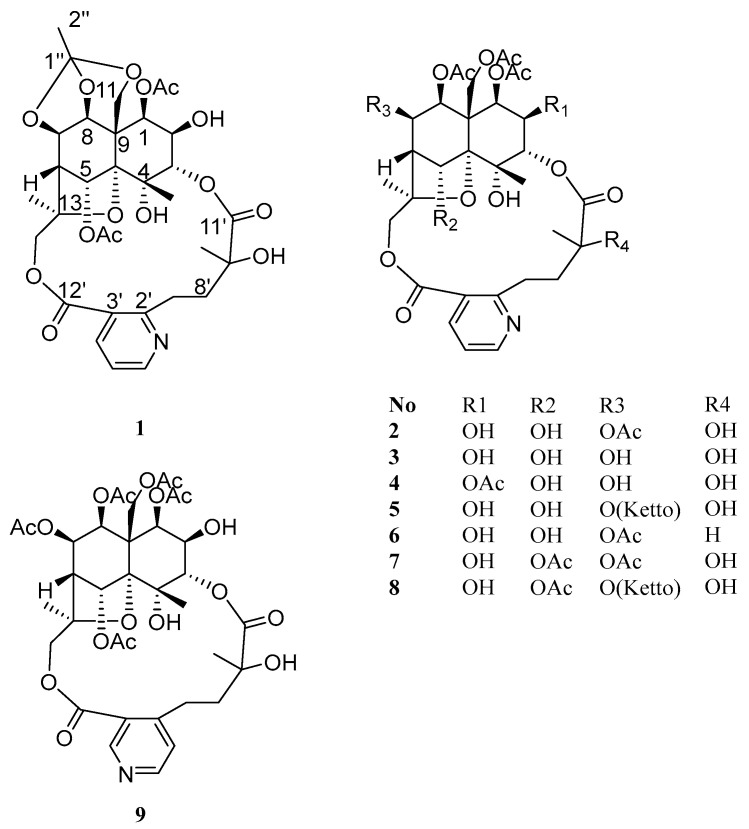
Structures of compounds **1**–**9**.

To further investigate structurally novel and biologically active alkaloids, a systematic phytochemical study was conducted on the alkaloid fraction of the roots of *T. wilfordii*. This study resulted in the isolation of nine SPAs (**1**–**9**), including four new compounds—wilforidatine A (**1**), 2,5-dideacetylalatusinine (**2**), 2,5,7-trideacetylalatusinine (**3**), and 5,7-dideacetylalatusinine (**4**)—along with five known analogues—tripterygiumine T (**5**), 5-deacetylwilforjine (**6**), tripfordine A (**7**), chiapenine ES-IV (**8**), and wilfordsuine (**9**) (Figure 1). Notably, compound **1** exhibited an unprecedented cage-like ether moiety formed by the linkage of C-7, C-8, and C-11 with a 1,1,1-trihydroxyethane unit, representing a novel structural archetype within the class of alkaloids [2]. Evaluation of the anti-inflammatory activity of the isolated compounds using a lipopolysaccharide (LPS)-induced RAW264.7 macrophage model demonstrated that compounds **1**, **2**, **4**, **5** and **9** significantly inhibited nitric oxide (NO) production, with IC_50_ values of 7.14 ± 1.89, 8.55 ± 0.37, 14.76 ± 0.39, 4.88 ± 0.92 and 2.43 ± 0.18 μM, respectively. Furthermore, compounds **1** and **2** markedly suppressed the secretion of inflammatory cytokines, including TNF-*α*, IL-6, and IL-1*β*, in the same cellular model. These findings not only enhanced the structural diversity of SPAs from *T. wilfordii* but also highlighted their potential as promising anti-inflammatory lead compounds worthy of further investigation.

## 2. Results and Discussion

### 2.1. Structural Elucidation

**Figure 2:**
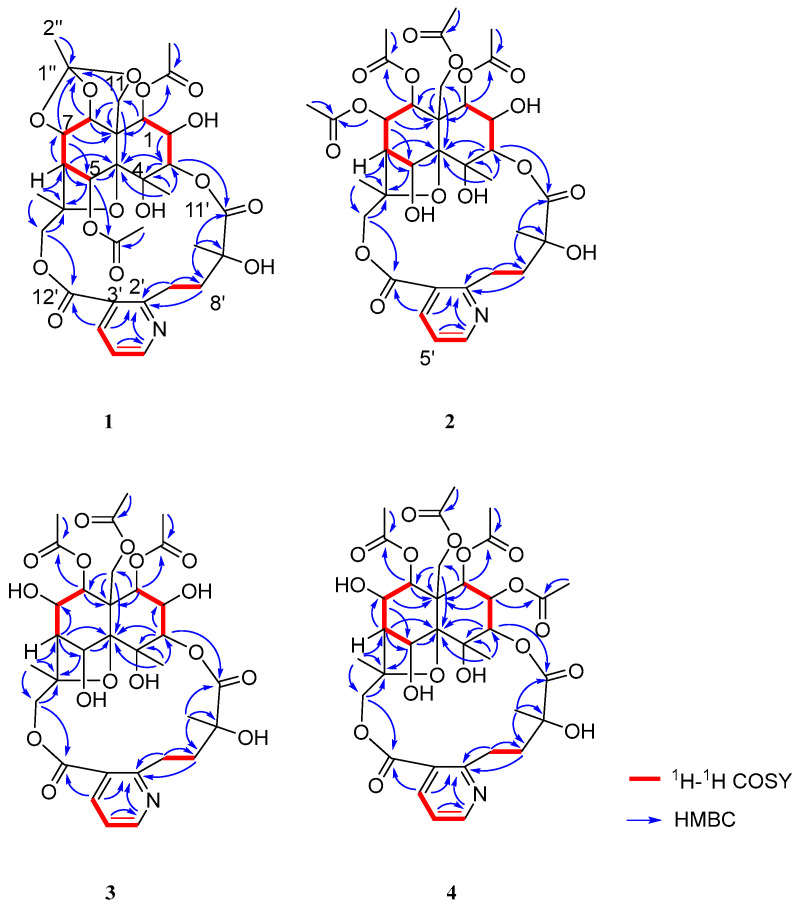
The key ^1^H-^1^H COSY and HMBC correlations of **1**–**4**.

Compound **1** was obtained as a white amorphous powder, soluble in chloroform and methanol. HR-ESI-MS exhibited a pseudo-molecular ion peak at *m*/*z* 678.2382 [M + H]^+^ (calcd C_32_H_39_O_15_N, 678.2393), consistent with 11 degrees of unsaturation. Analysis of its NMR data (Table 1 and Table 2) indicated that **1** was a 9′-hydroxylated W-type SPA, structurally related to **2**. Key differences included: (1) the presence of only two acetyl groups in **1**, two fewer than in **2**; (2) a significant downfield shift in the H-5 methine signal from *δ*_H_ 5.26 to *δ*_H_ 6.60 (1H, brs); (3) the appearance of an additional low-field quaternary carbon signal at *δ*_C_ 119.9 and a methyl group at *δ*_H_ 1.65 (3H, s) in the NMR spectra of **1**. In the HMBC spectrum, correlations from H-1 and H-5 to a carbonyl carbon at *δ*_C_ 169.6 (1-OAc) confirmed the presence of two acetyl groups attached to C-1 and C-5, respectively. Furthermore, key HMBC correlations from the methyl protons (*δ*_H_ 1.65) to the quaternary carbon (*δ*_C_ 119.9), and from H-7, H-8, and H-11 to the same carbon, suggested that C-7, C-8, and C-11 were connected via ether bonds to this quaternary carbon (Figure 2), which is itself linked to a methyl group, forming an unprecedented cage-like ether moiety. Literature consultation revealed that this structural feature, while known in compounds from Meliaceae family plants [10], was reported here for the first time in *T. wilfordii*.

The relative configuration of **1** was determined by analysis of ROESY correlations: H-8/H-1*α*, H-7, and H-14; H-12/H-3, H-5, and H-11*β*; and H-1/H-2. These data indicated the stereochemistry of the oxygenated substituents as 1*β*, 2*β*, 3*α*, 4*α*, 5*α*, 7*β*, and 8*β*, which was consistent with that of **2**. The structure of **2**, designated wilforidatine A, was established as shown in (Figure 1).

Compound **2**, obtained as a white amorphous powder, was soluble in chloroform and methanol. Its molecular formula was established as C_34_H_43_O_17_N by HR-ESI-MS (*m*/*z* 738.2603 [M + H]^+^, calcd 738.2604), indicated nine degrees of unsaturation. The ^1^H-NMR spectrum displayed signals for three methyl groups [*δ*_H_ 1.93 (3H, d, *J* = 1.2 Hz, H-12), 1.58 (3H, s, H-14), 1.58 (3H, s, H-10′)], two oxygenated methylene groups [*δ*_H_ 5.39 (1H, d, *J* = 13.2 Hz, H-11a), 4.61 (1H, d, *J* = 13.2 Hz, H-11b), 5.91 (1H, d, *J* = 12.6 Hz, H-15a), 3.68 (1H, d, *J* = 12.6 Hz, H-15b)], six oxygenated methine protons [*δ*_H_ 5.58 (1H, d, *J* = 3.0 Hz, H-1), 5.50 (1H, dd, *J* = 5.4, 4.2 Hz, H-7), 5.31 (1H, d, *J* = 6.0 Hz, H-8), 5.26 (1H, d, *J* = 3.0 Hz, H-5), 5.10 (1H, d, *J* = 3.0 Hz, H-3), 3.96 (1H, t, *J* = 3.0 Hz, H-2)], two methylene signals [*δ*_H_ 4.02 (1H, m, H-7a), 2.83 (1H, m, H-7b), 2.38 (1H, m, H-8a), 2.13 (1H, m, H-8b)], one methine [*δ*_H_ 2.44 (1H, d, *J* = 3.6 Hz, H-6)], and a set of aromatic protons indicative of a 2, 3-disubstituted pyridine ring [*δ*_H_ 8.68 (1H, dd, *J* = 4.8, 1.8 Hz, H-6′), 8.12 (1H, dd, *J* = 7.8, 1.8 Hz, H-4′), 7.21 (1H, dd, *J* = 7.8, 4.8 Hz, H-5′)]. Additionally, four acetyl methyl signals were observed [*δ*_H_ 2.18 (3H, s, 7-OAc), 2.17 (3H, s, 11-OAc), 1.96 (3H, s, 8-OAc), 1.95 (3H, s, 1-OAc)]. The ^13^C-NMR and DEPT spectra confirmed the presence of 34 carbon resonances, including four oxygenated quaternary carbons [*δ*_C_ 71.8 (C-4), 93.6 (C-10), 85.4 (C-13), 77.8 (C-9′)] and six carbonyl carbons [*δ*_C_ 170.1 (1-OAc), 170.2 (7-OAc), 169.2 (8-OAc), 169.4 (11-OAc), 173.2 (C-11′), 168.5 (C-12′)]. The ^1^H-^1^H COSY spectrum revealed two distinct spin systems: H-1/H-2/H-3 and H-5/H-6/H-7/H-8. The key HMBC correlations from H-1 to C-9 and C-11; H-3 to C-4 and C-5; H-5 to C-10 and C-13; H-6 to C-5 and C-10; H-7 to C-5, C-6, C-8, and C-9; H-8 to C-1, C-9, and C-11; H-11 to C-8, C-9, and C-10; H-12 to C-3, C-4, and C-10; H-14 to C-6, C-13, and C-15; and H-15 to C-13 and C-14 allowed the construction of a highly oxidized dihydro-*β*-agarofuran [9] sesquiterpene skeleton. Furthermore, the ^1^H-^1^H COSY correlations of H-4′/H-5′/H-6′ and H-7′/H-8′, along with HMBC correlations from H-7′ to C-2′ and C-8′; H-8′ to C-2′; H-4′ to C-12′; and H-10′ to C-8′, C-9′, and C-11′ supported the presence of a 3-carboxy-*α*-methyl picolinic acid unit. The linkage between the sesquiterpene unit and the picolinic acid moiety was established via ester bonds between C-3-O-C-11′ and C-15-O-C-12′, as evidenced by HMBC correlations from H-3 to C-11′ and from H-15 to C-12′ (Figure 2). Thus, the structure of **2** was determined as a 9′-hydroxy W-type SPA [2]. The planar structure of **2** was unequivocally established by key HMBC correlations from H-1 to the carbonyl carbon at *δ*_C_ 170.1 (1-OAc), H-7 to *δ*_C_ 170.2 (7-OAc), H-8 to *δ*_C_ 169.2 (8-OAc), and H-11 to *δ*_C_ 169.4 (11-OAc). These observations confirmed the presence of four acetoxy groups attached to C-1, C-7, C-8, and C-11, respectively.

**Figure 3:**
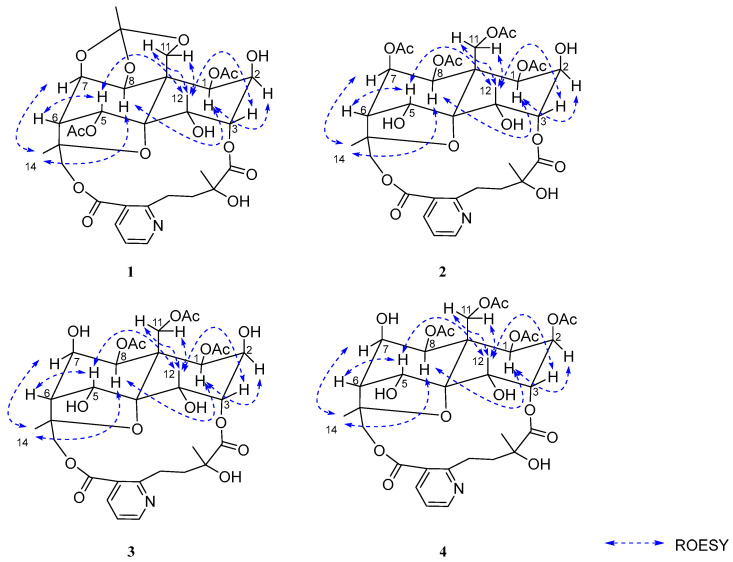
The key ROESY correlations of **1**–**4**.

The relative configuration of **2** was determined by analysis of the ROESY spectrum. Key ROESY correlations were observed between H-8/H-1*α*, H-7, and H-14; and between H-12/H-3, H-5, and H-11*β*. An additional correlation between H-1 and H-2 was also detected. Based on these findings, the relative stereochemistry of the oxygenated substituents was assigned as 1*β*, 2*β*, 3*α*, 4*α*, 5*α*, 7*β*, and 8*β* (Figure 3). In conclusion, the structure of compound **1** was determined as shown in (Figure 1). Comparison of its NMR data with those reported in the literature revealed that **2** is an analogue of the known compound alatusinine [9], differing by the absence of two acetyl groups at C-2 and C-5. Consequently, it was identified as 2,5-dideacetylalatusinine.

Compound **3** was obtained as a white amorphous powder, soluble in chloroform and methanol. HR-ESI-MS displayed a pseudo-molecular ion peak at *m*/*z* 696.2477 [M+H]^+^, corresponding to the molecular formula C_31_H_41_O_16_N (calcd. 696.2498), indicating eight degrees of unsaturation. Comparison of its ^1^H- and ^13^C-NMR data with those of compound **2** indicated a close structural similarity, suggesting that **3** also belongs to the class of 9′-hydroxylated W-type SPAs [2]. However, the ^1^H-NMR spectrum of **3** lacked one set of acetyloxy signals compared to **2**, and the H-7 proton resonance experienced a significant upfield shift from *δ*_H_ 5.50 to *δ*_H_ 4.25 (1H, m), indicating the absence of an acetyl group at C-7. Thus, **3** was deduced to be the 7-deacetyl derivative of **2**. The positions of the three remaining acetyl groups were established at C-1, C-8, and C-11 based on key HMBC correlations from H-1 to the carbonyl carbon at *δ*_C_ 169.9 (1-OAc), H-8 to *δ*_C_ 169.28 (8-OAc), and H-11 to *δ*_C_ 169.31 (11-OAc) (Figure 2), thereby confirming the planar structure of **3**.

The relative configuration was determined through analysis of the ROESY spectrum. Key correlations observed between H-8/H-1*α*, H-7, and H-14; H-12/H-3, H-5, and H-11*β*; and H-1/H-2 indicated that the stereochemistry of the oxygenated substituents was consistent with that of **2** (Figure 3), assigned as 1*β*, 2*β*, 3*α*, 4*α*, 5*α*, 7*β*, and 8*β*. In conclusion, the structure of compound **3** was determined as shown in (Figure 1). It is identified as the 7-deacetyl analogue of **2** and was consequently named 2,5,7-trideacetylalatusinine.

Compound **4** was obtained as a white amorphous powder. Its molecular formula was established as C_34_H_43_O_17_N by HR-ESI-MS (*m*/*z* 738.2584 [M + H]^+^, calcd 738.2604), corresponding to nine degrees of unsaturation. Comparison of its 1D NMR data (Table 1 and Table 2) with those of **3** indicated a high degree of structural similarity, confirming that **4** also belongs to the class of 9′-hydroxylated W-type SPAs. However, the ^1^H-NMR spectrum of **4** displayed an additional acetyl signal relative to **3**, and the H-2 proton resonance experienced a downfield shift from *δ*_H_ 3.96 to *δ*_H_ 5.13 (1H, m), indicating acetylation at the C-2 position. These observations suggested that **4** is the 2-acetyl derivative of **3**. The locations of the four acetyl groups were confirmed by key HMBC correlations: from H-1 to *δ*_C_ 169.6 (1-OAc), H-2 to *δ*_C_ 168.52 (2-OAc), H-8 to δC 169.1 (8-OAc), and H-11 to *δ*_C_ 169.3 (11-OAc) (Figure 2), thereby unambiguously establishing the planar structure of **4**. The relative configuration was determined through analysis of the ROESY spectrum, which showed correlations between H-8/H-1α, H-7, and H-14; H-12/H-3, H-5, and H-11*β*; and H-1/H-2 (Figure 3). These data indicated that the stereochemistry of the oxygenated substituents (1*β*, 2*β*, 3*α*, 4*α*, 5*α*, 7*β*, 8*β*) was consistent with that of **3**. In conclusion, the structure of **4** was determined as shown in (Figure 1) and was identified as the 2-acetyl analogue of **3**. It was consequently named 5,7-dideacetylalatusinine.

The five known compounds **5**–**9** were identified as tripterygiumine T (**5**) [11], 5-deacetylwilforjine (**6**) [6], tripfordine A (**7**) [12], chiapenine ES-IV (**8**) [9], and wilfordsuine (**9**) [13], by comparison of their spectroscopic data with reported data.

### 2.2. Biological Assay

**Figure 4:**
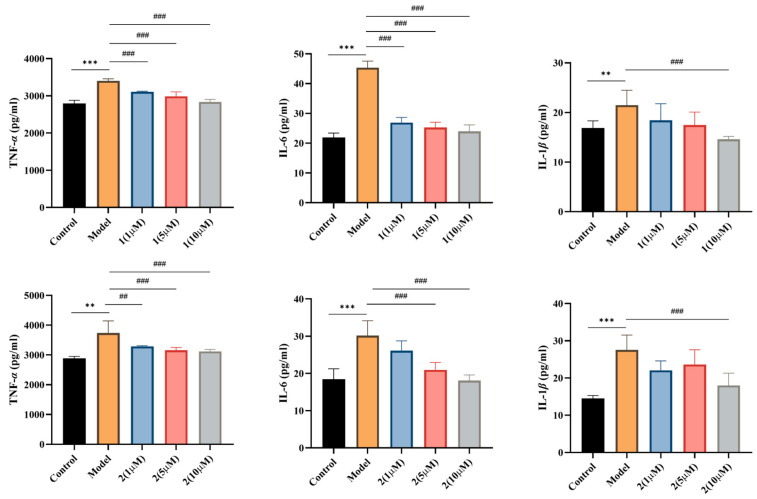
Compounds **1** and **2** significantly reduced the release levels of key inflammatory mediators TNF-*α*, IL-6, and IL-1*β*. (** *p* < 0.01, *** *p* < 0.001) vs. control group; (^##^ *p* < 0.01, ^###^ *p* < 0.001) vs. model group.

The anti-inflammatory activities of the isolated compounds (**1**–**9**) were evaluated using a lipopolysaccharide (LPS)-induced RAW264.7 macrophage model [2,3]. The inhibitory effects on nitric oxide (NO) production were measured, and the results were presented in (Table 3). Among the evaluated compounds, **1**, **2**, **4**, **5** and **9** exhibited significant concentration-dependent inhibition of NO production, with IC_50_ values ranging from 2.43 to 14.76 μM. Notably, **9** demonstrated the most potent activity (IC_50_ = 2.43 ± 0.18 μM). Compounds **1** and **5** also showed strong inhibitory effects, with IC_50_ values of 7.14 ± 1.89 μM and 4.88 ± 0.92 μM, respectively. Additionally, the effects of the two most active compounds (**1**, **2**) on the secretion of key pro-inflammatory cytokines were examined. As shown in (Figure 4), both compounds significantly suppressed the production of TNF-*α*, IL-6, and IL-1*β* in a concentration-dependent manner. These findings indicated that the isolated sesquiterpene pyridine alkaloids (SPAs), compounds **1**, **2**, **4**, **5**, and **9**, possess significant anti-inflammatory properties, potentially mediated through the suppression of NO and pro-inflammatory cytokine production. It should be noted that the findings of this study, derived from an in vitro model, provide preliminary evidence of anti-inflammatory potential. However, further investigation is required to elucidate the underlying mechanisms of action.

## 3. Experimental Section

### 3.1. Biological Assay

The instrumentation and reagents employed in this study were similar to those in our previous reports [2].

### 3.2. Extraction and Isolation

The dried roots of *Tripterygium wilfordii* (50 kg) were pulverized and exhaustively extracted three times with 10 volumes of 95% ethanol under reflux. The combined filtrates were concentrated under reduced pressure to afford a crude total extract. This extract was subsequently dispersed in water and partitioned three times with an equal volume of chloroform. The organic phases were combined and evaporated under reduced pressure, yielding a chloroform-soluble extract (200 g). A portion of this extract (200 g) was dissolved in a suitable amount of ethyl acetate and subjected to column chromatography over neutral alumina, using ethyl acetate as the eluent. After solvent removal, 68 g of an enriched fraction was obtained. This fraction (50 g) was further separated by dry loading on an ODS reverse-phase column, with a gradient elution of methanol-water (10:90 → 100:0, *v*/*v*), resulting in the collection of 16 fractions (Fr.1-16). Fraction Fr.9 (7.965 g) was first separated by preparative HPLC on a Waters XBridge Prep OBD C_18_ column (5 µm, 19 × 250 mm), using an isocratic eluent of ACN-H_2_O (23:77, *v*/*v*) at a flow rate of 16 mL/min, to yield seven subfractions (Fr.9-1 to Fr.9-7). Subfraction Fr.9-2 (260 mg) was further purified by semipreparative HPLC on a Waters XSelect HSS T3 OBD Prep C_18_ column (5 µm, 10 × 250 mm) with CAN-H_2_O (30:70, *v*/*v*; 4 mL/min), affording five fractions (Fr.9-2-1 to Fr.9-2-5). Fr.9-2-1 (139 mg) was subsequently chromatographed on a YMC-Triart Phenyl column (5 µm, 10 × 250 mm) under isocratic conditions [ACN-H_2_O (30:70, *v*/*v*); 4 mL/min] to yield compounds **5** (4.56 mg, *t*_R_ 20 min) and **1** (2.32 mg, *t*_R_ 22 min). Subfraction Fr.9-4 (450 mg) was separated on a YMC-Pack Ph column (5 µm, 10 × 250 mm) using CAN-H_2_O (33:67, *v*/*v*; 4 mL/min), giving two pooled fractions (Fr.9-4-1 and Fr.9-4-2). Fr.9-4-1 (290 mg) was then subjected to semipreparative HPLC on a Waters XSelect HSS T3 OBD Prep C_18_ column with ACN-H_2_O (28:72, *v*/*v*; 4 mL/min), yielding compounds **3** (0.84 mg, *t*_R_ 17 min) and **4** (9.96 mg, *t*_R_ 19 min). Purification of Fr.9-4-2 (54 mg) on a YMC-Triart Phenyl column [ACN-H_2_O (30:70, *v*/*v*); 4 mL/min] afforded compound **2** (17.55 mg, *t*_R_ 20 min). Subfraction Fr.9-5 (150 mg) was fractionated on a Dikam Inspire C_18_ column (5 µm, 10 × 250 mm) with MeOH-H_2_O (50:50, *v*/*v*; 4 mL/min) to give five fractions (Fr.9-5-1 to Fr.9-5-5). Fr.9-5-1 (59 mg) was re-chromatographed on the same Dikam Inspire C_18_ column under isocratic conditions [ACN-H_2_O (30:70, *v*/*v*); 4 mL/min], yielding compounds **6** (4 mg, *t*_R_ 19 min) and **7** (15 mg, *t*_R_ 24 min). Subfraction Fr.9-6 (312 mg) was separated on a Waters XSelect HSS T3 OBD Prep C_18_ column using ACN-H_2_O (28:72, *v*/*v*; 4 mL/min), providing four fractions (Fr.9-6-1 to Fr.9-6-4). Final purification of Fr.9-6-4 (75 mg) on a YMC-Triart Phenyl column [ACN-H_2_O (30:70, *v*/*v*); 2 mL/min] yielded compound **8** (37 mg, *t*_R_ 60 min). Subfraction Fr.9-7 (380 mg) was directly purified by semipreparative HPLC on a YMC-Triart Phenyl column with ACN-H_2_O (29:71, *v*/*v*; 4 mL/min) to afford compound **9** (8.91 mg, *t*_R_ 35 min).

### 3.3. Characterization of New Compounds

*Wilforidatine A* (**4**): White amorphous powder; ${\left[\alpha \right]}_{D}^{25}$= 30 (c 0.03, MeOH); UV (MeOH) λ_max_ (log *ε*) = 202 (4.31), 220 (3.95), 268 (3.43) nm; IR *v*_max_ 3336, 1740, 1372, 1229, 1047 cm^−1^; ^1^H NMR and ^13^C NMR spectral data, see Table 1 and Table 2; HRESIMS: *m*/*z* 678.2382 [M + H]^+^ (calcd for C_32_H_40_O_15_N, 678.2393).
*2,5-dideacetylalatusinine* (**2**): White amorphous powder, ${\left[\alpha \right]}_{D}^{25}$ = 8.6 (c 0.06, MeOH); UV (MeOH) λ_max_ (log *ε*) = 202 (4.28), 214 (4.12), 268 (3.65) nm; IR *v*_max_ 3356, 1740, 1372, 1256, 1045, 709 cm^−1^; ^1^H NMR and ^13^C NMR spectral data, see Table 1 and Table 2; HRESIMS: *m*/*z* 738.2603 [M + H]^+^ (calcd for C_34_H_44_NO_17_, 738.2604).
*2,5,7-trideacetylalatusinine* (**3**): White amorphous powder; ${\left[\alpha \right]}_{D}^{25}$= 15.0 (c 0.03, MeOH); UV (MeOH) λ_max_ (log *ε*) = 202 (4.22), 219 (3.92), 268 (3.32) nm; IR *v*_max_ 1742, 1370, 1236, 1045, 1033, 953 cm^−1^; ^1^H NMR and ^13^C NMR spectral data, see Table 1 and Table 2; HRESIMS: *m*/*z* 696.2477 [M + H]^+^ (calcd for C_31_H_42_O_16_N_,_ 696.2498).
*5,7-dideacetylalatusinine* (**4**): White amorphous powder; ${\left[\alpha \right]}_{D}^{25}$= −39.0 (c 0.03, MeOH); UV (MeOH) λ_max_ (log *ε*) = 203 (4.44), 215 (4.25), 268 (3.80) nm; IR *v*_max_ 3336, 1735, 1373, 1248, 1229, 1079, 954 cm^−1^; ^1^H NMR and ^13^C NMR spectral data, see Table 1 and Table 2; HRESIMS: *m*/*z* 738.2584 [M + H]^+^ (calcd for C_34_H_44_O_17_N, 738.2604).

### 3.4. Cell Culture

See Supplementary Materials.

### 3.5. Anti-Inflammatory Activity Assays

See Supplementary Materials.

## 4. Conclusions

In conclusion, a phytochemical investigation of the roots of *Tripterygium wilfordii* Hook. f. resulted in the isolation and structural characterization of nine sesquiterpene pyridine alkaloids (SPAs). Among these, four were identified as previously undescribed compounds (**1**–**4**), while five were known analogues (**5**–**9**). The structures of the new alkaloids were unequivocally established through comprehensive analysis of spectroscopic data, including 1D and 2D NMR and HRESMS. Notably, compound **1** possessed a unique cage-like ether moiety. This structural feature represents a novel archetype within the SPA family isolated from this plant source.

Evaluation of the anti-inflammatory potential of the isolated compounds demonstrated that several, particularly **1**, **2**, **4**, **5**, and **9**, significantly inhibited the production of nitric oxide (NO) and key pro-inflammatory cytokines (TNF-*α*, IL-6, IL-1*β*) in LPS-induced RAW 264.7 macrophages. The structure–activity relationship (SAR) analysis indicated that acetylation of hydroxyl groups at specific positions of the SPA skeleton can markedly enhance anti-inflammatory potency, providing critical insights for future medicinal chemistry optimization. This study enhanced the known chemical diversity of *T. wilfordii* and underscored the potential of its sesquiterpenoid alkaloids as valuable scaffolds for the development of novel anti-inflammatory agents. Further investigation into the precise molecular mechanisms and in vivo efficacy of these compounds is warranted to fully assess their therapeutic potential.

## Figures and Tables

**Table 1:** ^1^H-NMR data of compounds **1**–**4** (CDCl_3_, 600 MHz).

Position	*δ*_H_ (*J* in Hz)			
	1	2	3	4
1	5.54 (1H, d, 3.0)	5.58 (1H, d, 3.0)	5.51 (1H, d, 3.0)	5.58 (1H, d, 3.0)
2	3.90 (1H, m)	3.96 (1H, m)	3.97 (1H, brs)	5.13 (1H, m)
3	4.99 (1H, d, 3.6)	5.10 (1H, d, 3.0)	5.06 (1H, d, 3.0)	4.98 (1H, d, 3.0)
5	6.60 (1H, brs)	5.26 (1H, d, 3.0)	5.21 (1H, brs)	5.20 (1H, d, 3.0)
6	2.51 (1H, d, 3.0)	2.44 (1H, d, 3.6)	2.51 (1H, d, 4.2)	2.52 (1H, d, 3.6)
7	4.41 (1H, m)	5.50 (1H, dd, 5.4, 4.2)	4.25 (1H, m)	4.23 (1H, m)
8	4.40 (1H, d, 5.4)	5.31 (1H, d, 6.0)	5.29 (1H, d, 6.0)	5.29 (1H, d, 6.0)
11	3.98 (1H, d, 13.2) 4.14 (1H, d, 13.2)	4.61 (1H, d, 13.2) 5.39 (1H, d, 13.2)	4.61 (1H, d, 13.8) 5.22 (1H, d, 13.8)	4.47 (1H, d, 13.2) 5.12 (1H, d, 13.2)
12	1.66 (3H, d, 1.2)	1.93 (3H, d, 1.2)	1.91 (3H, brs)	1.88 (3H, d, 1.2)
14	1.45 (3H, s)	1.58 (3H, s)	1.53 (3H, s)	1.53 (3H, s)
15	3.77 (1H, d, 12.0) 5.79 (1H, d, 12.0)	3.68 (1H, d, 12.6) 5.91 (1H, d, 12.6)	3.70 (1H, d, 12.6) 5.93 (1H, d, 12.6)	3.69 (1H, d, 12.6) 5.91 (1H, d, 12.6)
4′	8.11 (1H, dd, 7.8, 1.8)	8.12 (1H, dd, 7.8, 1.8)	8.11 (1H, dd, 7.8, 1.8)	8.13 (1H, dd, 7.8, 1.8)
5′	7.21 (1H, dd, 7.8, 4.8)	7.21 (1H, dd, 7.8, 4.8)	7.20 (1H, dd, 7.8, 4.8)	7.20 (1H, dd, 7.8, 4.8)
6′	8.67 (1H, dd, 4.8, 1.8)	8.68 (1H, dd, 4.8, 1.8)	8.68 (1H, dd, 4.8, 1.8)	8.68 (1H, dd, 4.8, 1.8)
7′	2.87 (1H, m) 3.94 (1H, m)	2.83 (1H, m) 4.02 (1H, m)	2.82 (1H, m) 4.04 (1H, m)	2.83 (1H, m) 4.07 (1H, m)
8′	2.20 (1H, m) 2.47 (1H, m)	2.13 (1H, m) 2.38 (1H, m)	2.13 (1H, m) 2.42 (1H, m)	2.13 (1H, m) 2.46 (1H, m)
10′	1.41 (3H, s)	1.42 (3H, s)	1.40 (3H, s)	1.44 (3H, s)
1-OAc	2.17 (3H, s)	1.95 (3H, s)	1.99 (3H, s)	1.89 (3H, s)
2-OAc				2.18 (3H, s)
5-OAc	2.18 (3H, s)			
7-OAc		2.18 (3H, s)		
8-OAc		1.96 (3H, s)	2.22 (3H, s)	2.09 (3H, s)
11-OAc		2.17 (3H, s)	2.10 (3H, s)	2.22 (3H, s)
4-OH	4.70 (1H, d, 1.2)	6.21 (1H, d, 1.2)		6.25 (1H, d, 1.2)
2″	1.65 (3H, s)			

**Table 2:** ^13^C-NMR data of compounds 1–4 (CDCl_3_, 150 MHz).

Position	*δ*_C_			
	1	2	3	4
1	72.4	75.2	74.8	72.8
2	70.5	70.5	70.1	68.7
3	78.1	77.9	78.1	75.8
4	69.9	71.8	71.6	71.6
5	73.9	74.2	73.69	73.6
6	49.0	52.4	54.6	54.4
7	75.2	69.3	68.2	68.3
8	77.3	71.5	73.66	73.2
9	54.9	51.0	52.5	52.0
10	93.3	93.6	93.7	93.1
11	59.0	61.3	61.5	61.0
12	23.6	23.8	23.5	23.2
13	85.2	85.4	85.2	85.2
14	18.4	18.1	17.9	17.8
15	69.5	70.9	71.1	70.9
2′	164.3	165.2	165.3	165.5
3′	126.1	125.4	125.6	125.3
4′	138.0	138.0	137.9	137.8
5′	120.8	120.9	120.8	120.6
6′	152.2	152.5	152.4	152.4
7′	31.2	31.6	31.5	31.4
8′	38.5	39.4	39.1	38.8
9′	77.5	77.8	78.0	78.1
10′	28.0	27.1	27.6	27.9
11′	172.6	173.2	173.2	172.6
12′	168.1	168.5	168.6	168.49
1-OAc	170.9/21.2	170.1/20.9	169.9/20.9	169.6/20.5
2-OAc				168.52/20.1
5-OAc	169.6/21.8			
7-OAc		170.2/21.1		
8-OAc		169.2/20.6	169.25/21.4	169.1/20.8
11-OAc		169.4/21.6	169.31/21.0	169.3/21.3
1″	119.9			
2″	21.9			

**Table 3:** NO inhibitory effects of **1**, **2**, **4**, **5** and **9** in LPS-induced RAW 264.7 cells.

Compound	Cell Viability (%) (10μM) (*n* = 3)	IC_50_ (μM) (*n* = 6)
**1**	107.06 ± 11.37	7.14 ± 1.89 μM
**2**	102.12 ± 9.05	8.55 ± 0.37 μM
**4**	115.39 ± 4.77	14.76 ± 0.39 μM
**5**	107.06 ± 11.37	4.88 ± 0.92 μM
**9**	114.23 ± 0.55	2.43 ± 0.18 μM

IC_50_ screening concentration of 0.1, 0.5, 1, 5, 10, 50 μM were used.

## Data Availability

The data presented in this study are available in the article and Supplementary Materials.

## Supplementary Materials

The following supporting information can be downloaded at: https://www.mdpi.com/article/10.3390/molecules31020271/s1. Figures S1–S44: 1D-, 2D-NMR, HRMS, UV, and IR spectra for the new compounds; Figures S45–S59: HRMS, 1H- and 13C-NMR spectra for the known compounds; Page 3 for Cell culture and Anti-inflammatory activity assays.
