# Supplementary material for: Four New Sesquiterpene Pyridine Alkaloids from the Roots of Tripterygium wilfordii Hook. f
Source: Molecules. 2026 Jan 13;31(2):271. doi: 10.3390/molecules31020271 (PMC12844141; doi:10.3390/molecules31020271)

Supporting information

Contents:

Cell culture 3
Anti-inflammatory activity assays 3
Figure S1. ^{1}H-NMR spectrum of compound **1** ($CDCl_3$, 600 MHz) 4
Figure S2. ^{13}C-NMR spectrum of compound **1** ($CDCl_3$, 150 MHz) 5
Figure S3. DEPT 135° spectrum of compound **1** 6
Figure S4. DEPT 90° spectrum of compound **1** 7
Figure S5. ^{1}H-^{1}H COSY spectrum of compound **1** 8
Figure S6. HSQC spectrum of compound **1** 9
Figure S7. HMBC spectrum of compound **1** 10
Figure S8. ROESY spectrum of compound **1** 11
Figure S9. IR spectrum of **1** 12
Figure S10. UV spectrum of **1** 13
Figure S11. HR-ESI-MS spectrum of compound **1** 14
Figure S12. ^{1}H-NMR spectrum of compound **2** ($CDCl_3$, 600 MHz) 15
Figure S13. ^{13}C-NMR spectrum of compound **2** ($CDCl_3$, 150 MHz) 16
Figure S14. DEPT 135° spectrum of compound **2** 17
Figure S15. DEPT 90° spectrum of compound **2** 18
Figure S16. ^{1}H-^{1}H COSY spectrum of compound **2** 19
Figure S17. HSQC spectrum of compound **2** 20
Figure S18. HMBC spectrum of compound **2** 21
Figure S19. ROESY spectrum of compound **2** 22
Figure S20. IR spectrum of **2** 23
Figure S21. UV spectrum of **2** 24
Figure S22. HR-ESI-MS spectrum of compound **2** 25
Figure S23. ^{1}H-NMR spectrum of compound **3** ($CDCl_3$, 600 MHz) 26
Figure S24. ^{13}C-NMR spectrum of compound **3** ($CDCl_3$, 150 MHz) 27
Figure S25. DEPT 135° spectrum of compound **3** 28
Figure S26. DEPT 90° spectrum of compound **3** 29
Figure S27. ^{1}H-^{1}H COSY spectrum of compound **3** 30
Figure S28. HSQC spectrum of compound **3** 31
Figure S29. HMBC spectrum of compound **30** 32
Figure S30. ROESY spectrum of compound **3** 33
Figure S31. IR spectrum of **3** 34
Figure S32. UV spectrum of **3** 35
Figure S33. HR-ESI-MS spectrum of compound **3** 36

Figure S34. ^{1}H-NMR spectrum of compound **4** ($CDCl_3$, 600 MHz) 37
Figure S35. ^{13}C-NMR spectrum of compound **4** ($CDCl_3$, 150 MHz) 38
Figure S36. DEPT 135° spectrum of compound **4** 39
Figure S37. DEPT 90° spectrum of compound **4** 40
Figure S38. ^{1}H-^{1}H COSY spectrum of compound **4** 41
Figure S39. HSQC spectrum of compound **4** 42
Figure S40. HMBC spectrum of compound **4** 43
Figure S41. ROESY spectrum of compound **4** 44
Figure S42. IR spectrum of **4** 45
Figure S43. UV spectrum of **4** 46
Figure S44. HR-ESI-MS spectrum of compound **4** 47
Figure S45. ^{1}H-NMR spectrum of compound **5** ($CDCl_3$, 600 MHz) 48
Figure S46. ^{13}C-NMR spectrum of compound **5** ($CDCl_3$, 150 MHz) 49
Figure S47. HR-ESI-MS spectrum of compound **5** 50
Figure S48. ^{1}H-NMR spectrum of compound **6** ($CDCl_3$, 600 MHz) 51
Figure S49. ^{13}C-NMR spectrum of compound **6** ($CDCl_3$, 150 MHz) 52
Figure S50. HR-ESI-MS spectrum of compound **6** 53
Figure S51. ^{1}H-NMR spectrum of compound **7** ($CDCl_3$, 600 MHz) 54
Figure S52. ^{13}C-NMR spectrum of compound **7** ($CDCl_3$, 150 MHz) 55
Figure S53. HR-ESI-MS spectrum of compound **7** 56
Figure S54. ^{1}H-NMR spectrum of compound **8** ($CDCl_3$, 600 MHz) 57
Figure S55. ^{13}C-NMR spectrum of compound **8** ($CDCl_3$, 150 MHz) 58
Figure S56. HR-ESI-MS spectrum of compound **8** 59
Figure S57. ^{1}H-NMR spectrum of compound **9** ($CDCl_3$, 600 MHz) 60
Figure S58. ^{13}C-NMR spectrum of compound **9** ($CDCl_3$, 150 MHz) 61
Figure S59. HR-ESI-MS spectrum of compound **9** 62

Cell culture

The RAW264.7 cells were obtained from Procell, China, and cultured in DMEM medium (Solarbio, China) supplemented with 10% fetal bovine serum (Gibco, USA) and 100 IU/mL penicillin/streptomycin (Solarbio, China). Cells were kept in a humidified incubator at 37 °C with 5% CO_2.

Anti-inflammatory activity assays

RAW264.7 cells were seeded at a density of 30,000 cells per well into 96-well plates. The following groups were established: blank control, solvent control, and drug treatment groups. Cells were stimulated with 1 μg/mL LPS for 24 h, followed by collection of cell supernatants, and the metabolite of nitric oxide (NO), using the Griess method to reflect NO production. To exclude interference from drug cytotoxicity, cell viability was assessed via the CCK8 assay. Finally, by comparing NO levels among drug groups and the model group, the inhibitory effect and potency of drugs on LPS-induced NO production were evaluated. Inflammatory cytokine expression in the model and treatment groups was measured using human interleukin-1β (IL-1β), human interleukin-6 (IL-16), and human tumor necrosis factor α (TNF-α) enzyme-linked immunosorbent assay (ELISA) kits.

Figure S1. ^{1}H-NMR spectrum of compound **1** ($CDCl_3$, 600 MHz)

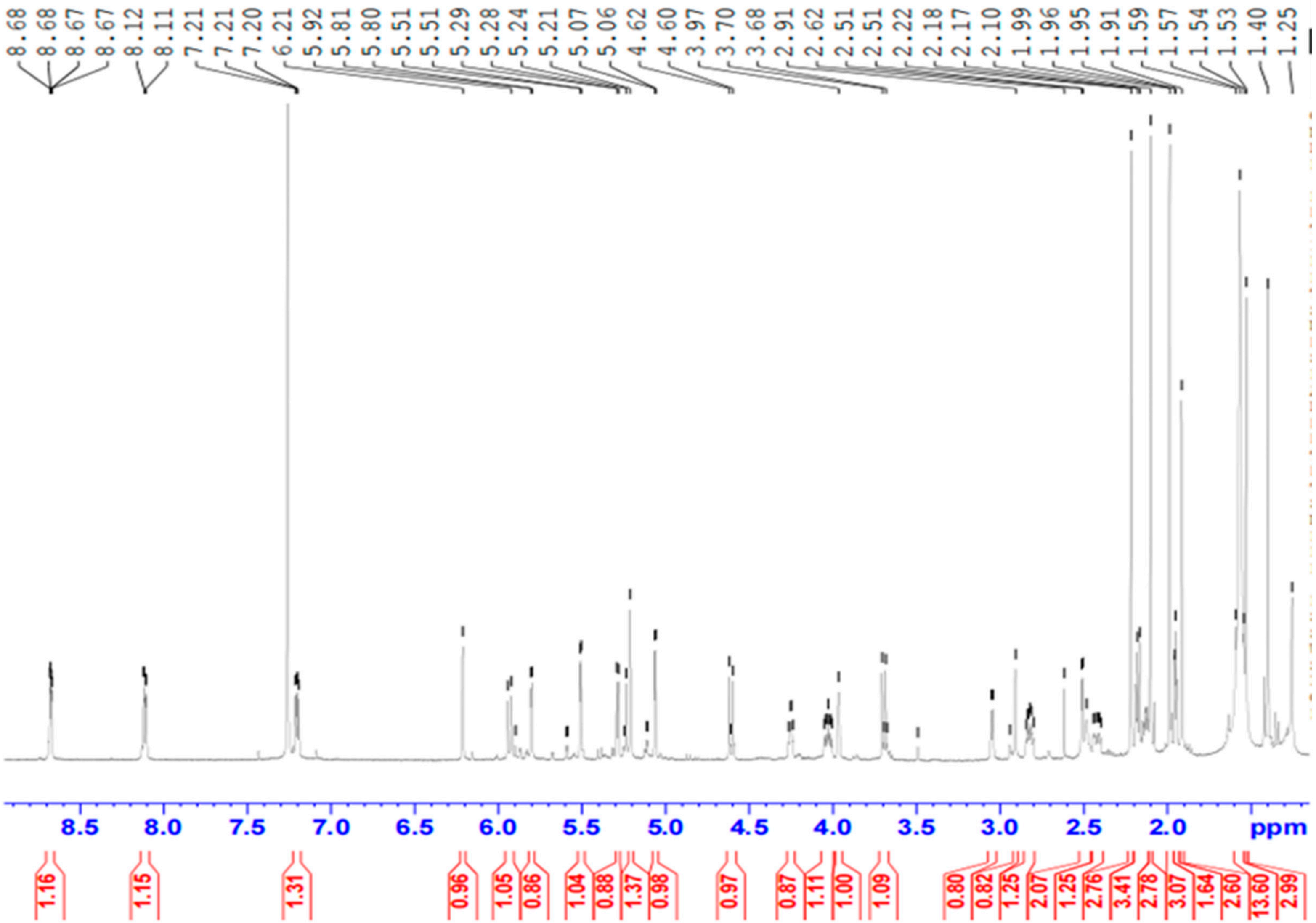

Figure S2. ^{13}C-NMR spectrum of compound **1** ($CDCl_3$, 150 MHz)

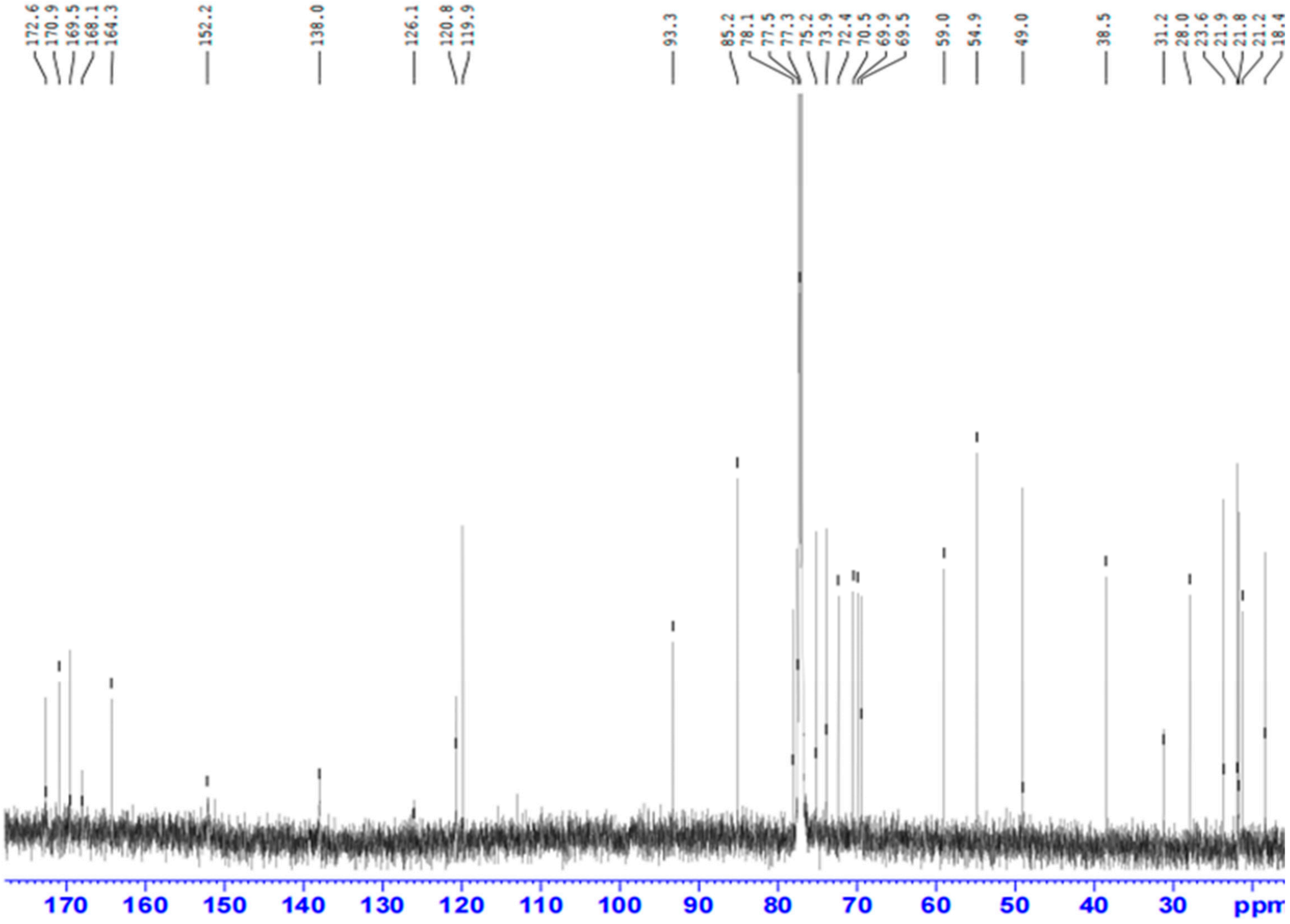

Figure S3. DEPT 135° spectrum of compound **1**

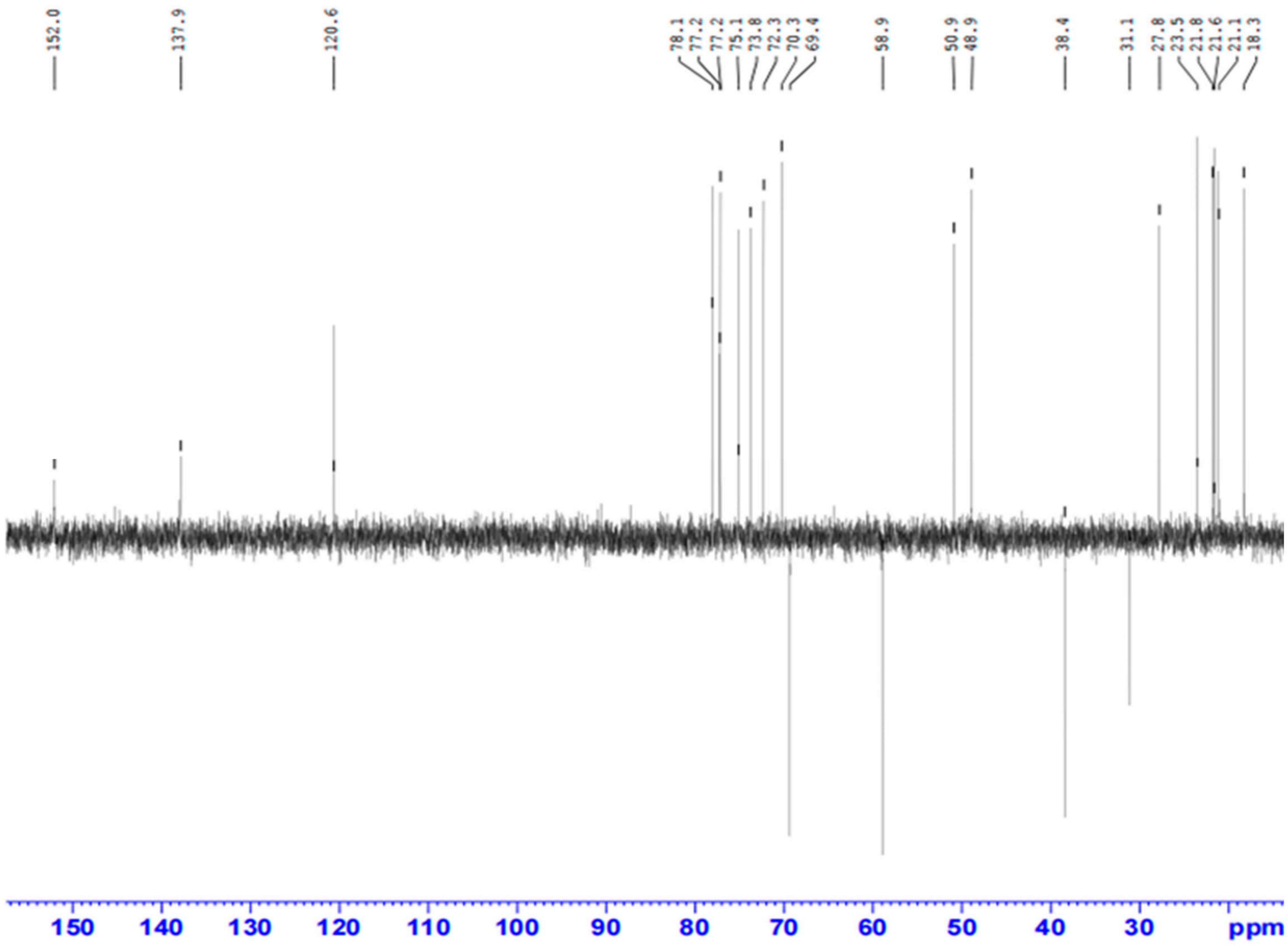

Figure S4. DEPT 90° spectrum of compound **1**

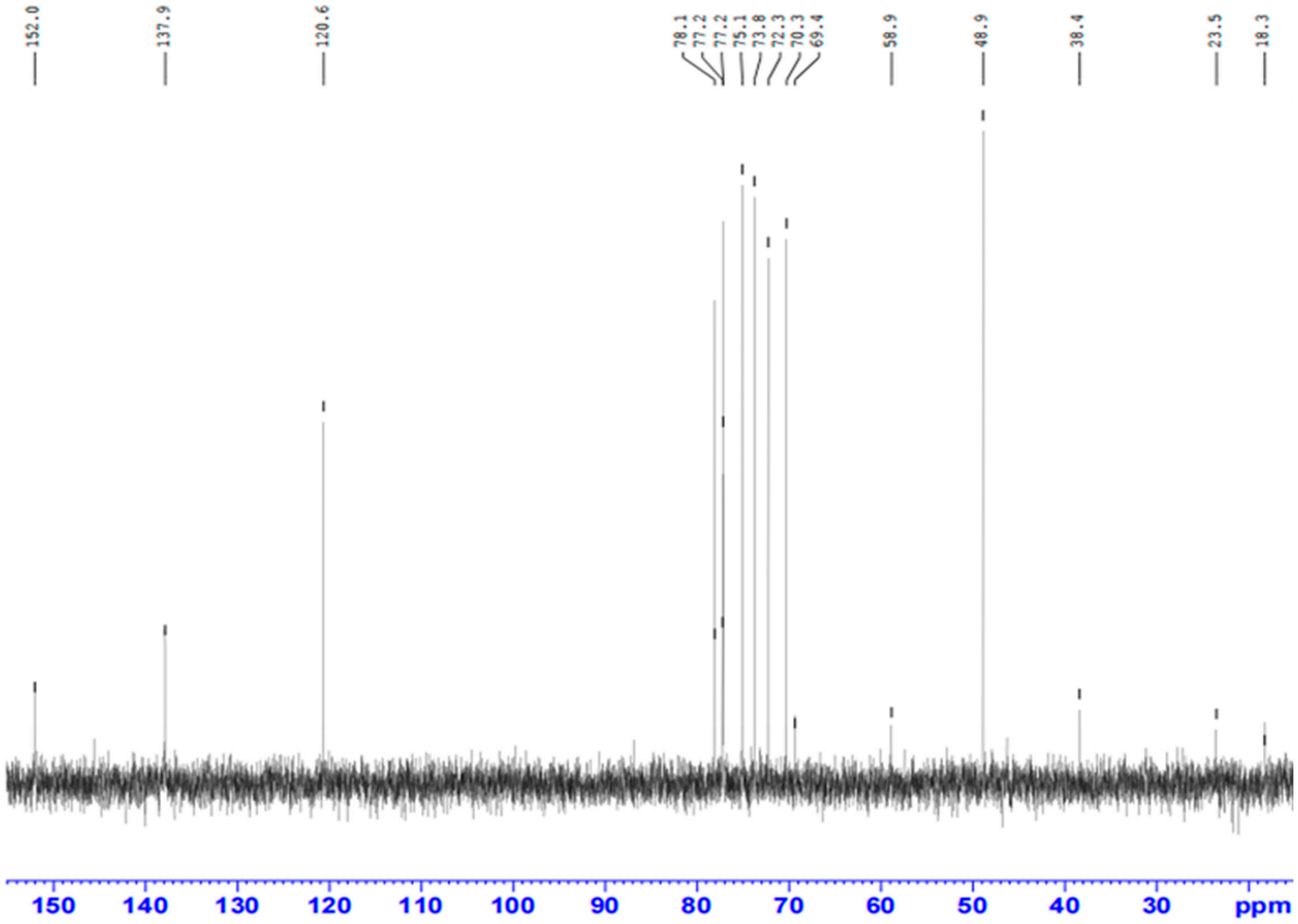

Figure S5. ^{1}H-^{1}H COSY spectrum of compound **1**

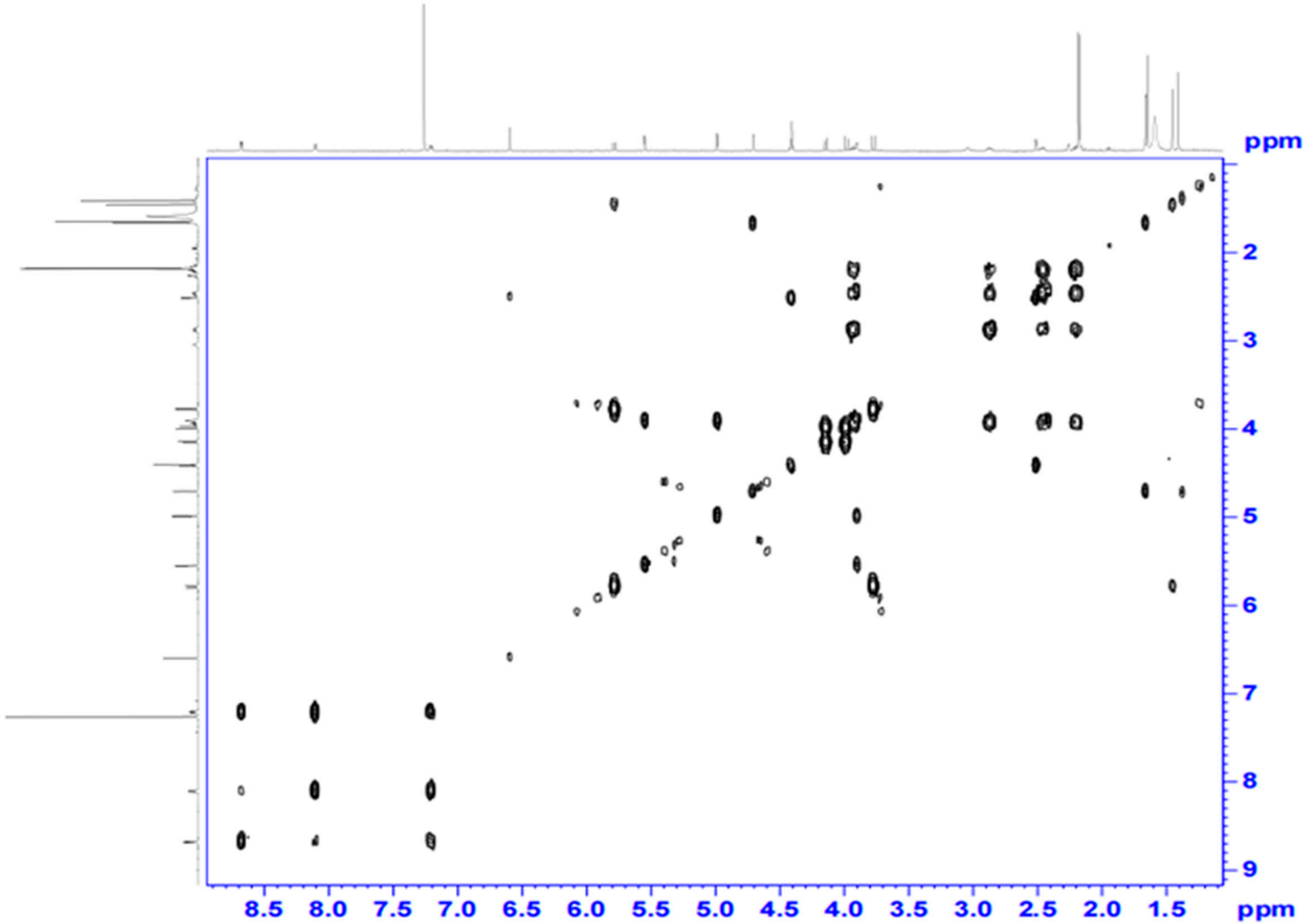

Figure S6. HSQC spectrum of compound **1**

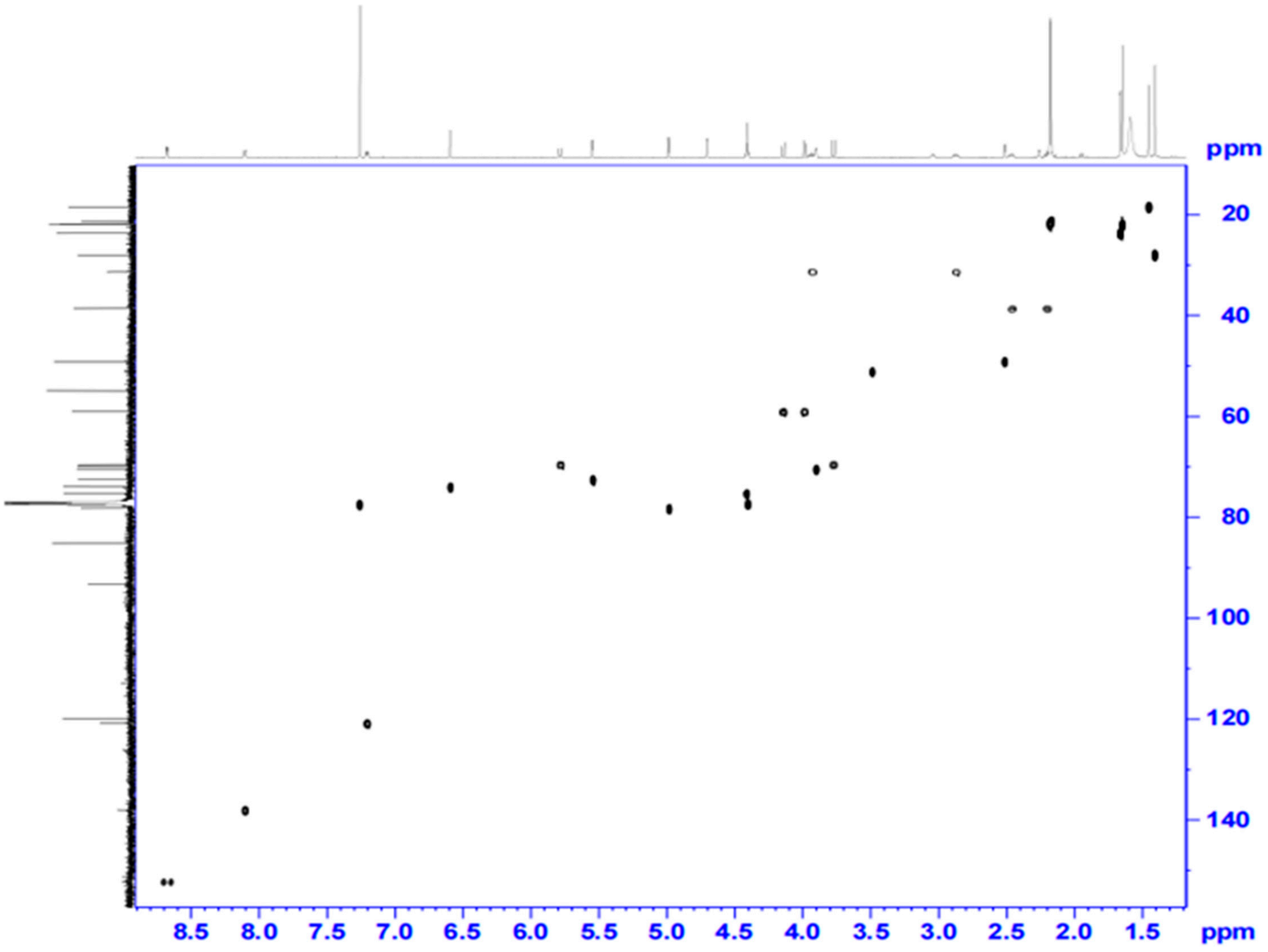

Figure S7. HMBC spectrum of compound **1**

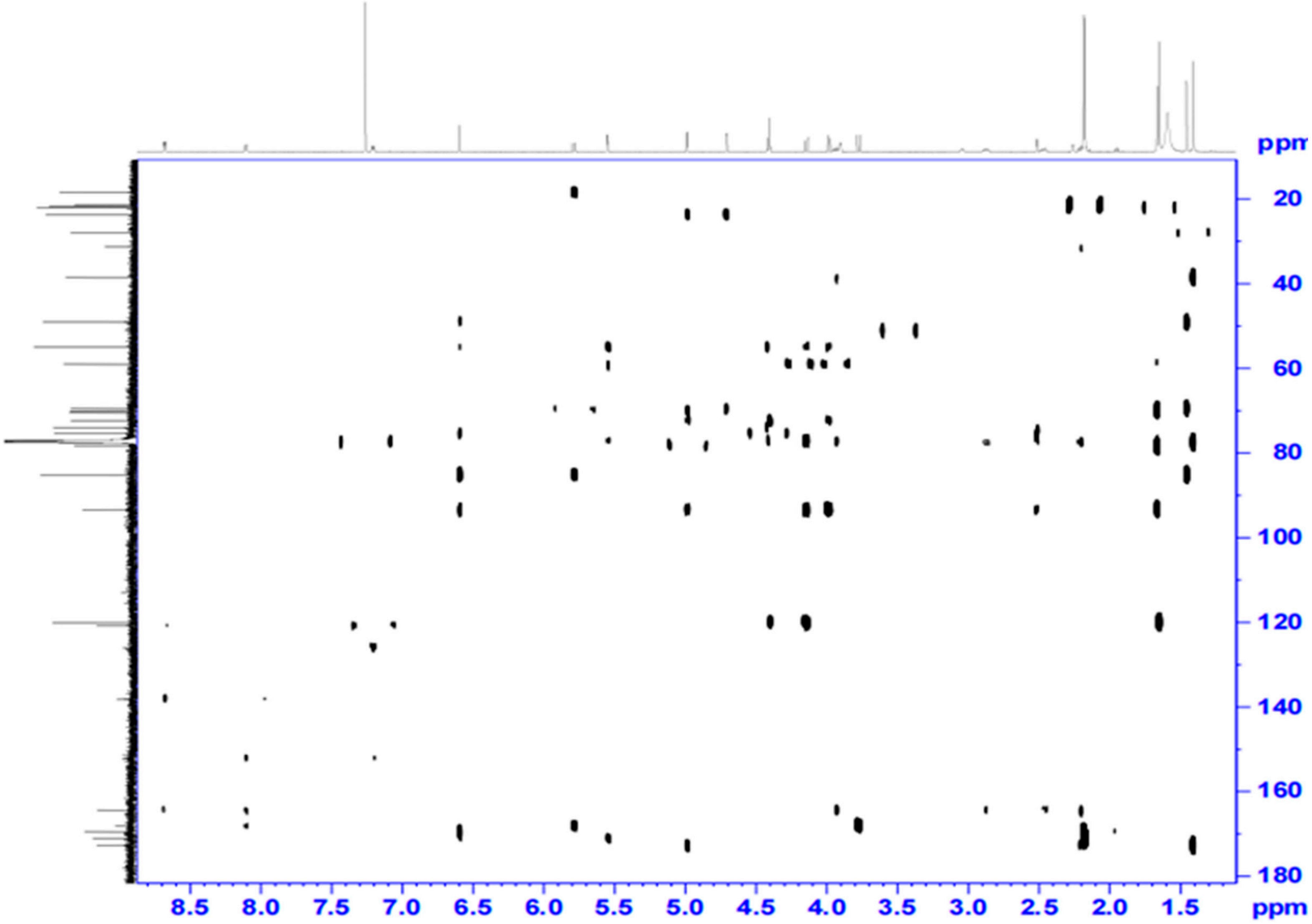

Figure S8. ROESY spectrum of compound **1**

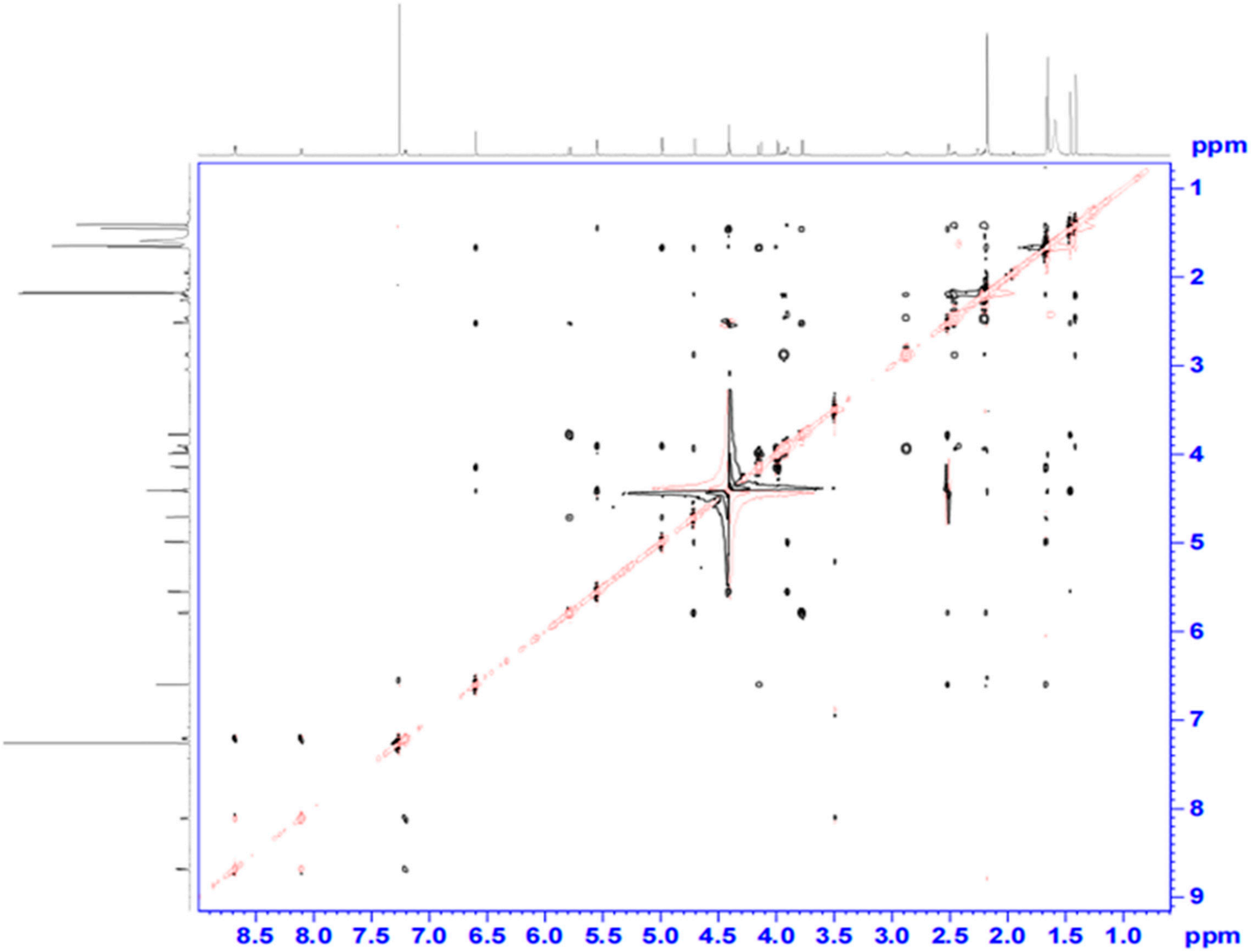

Figure S9. IR spectrum of **1**

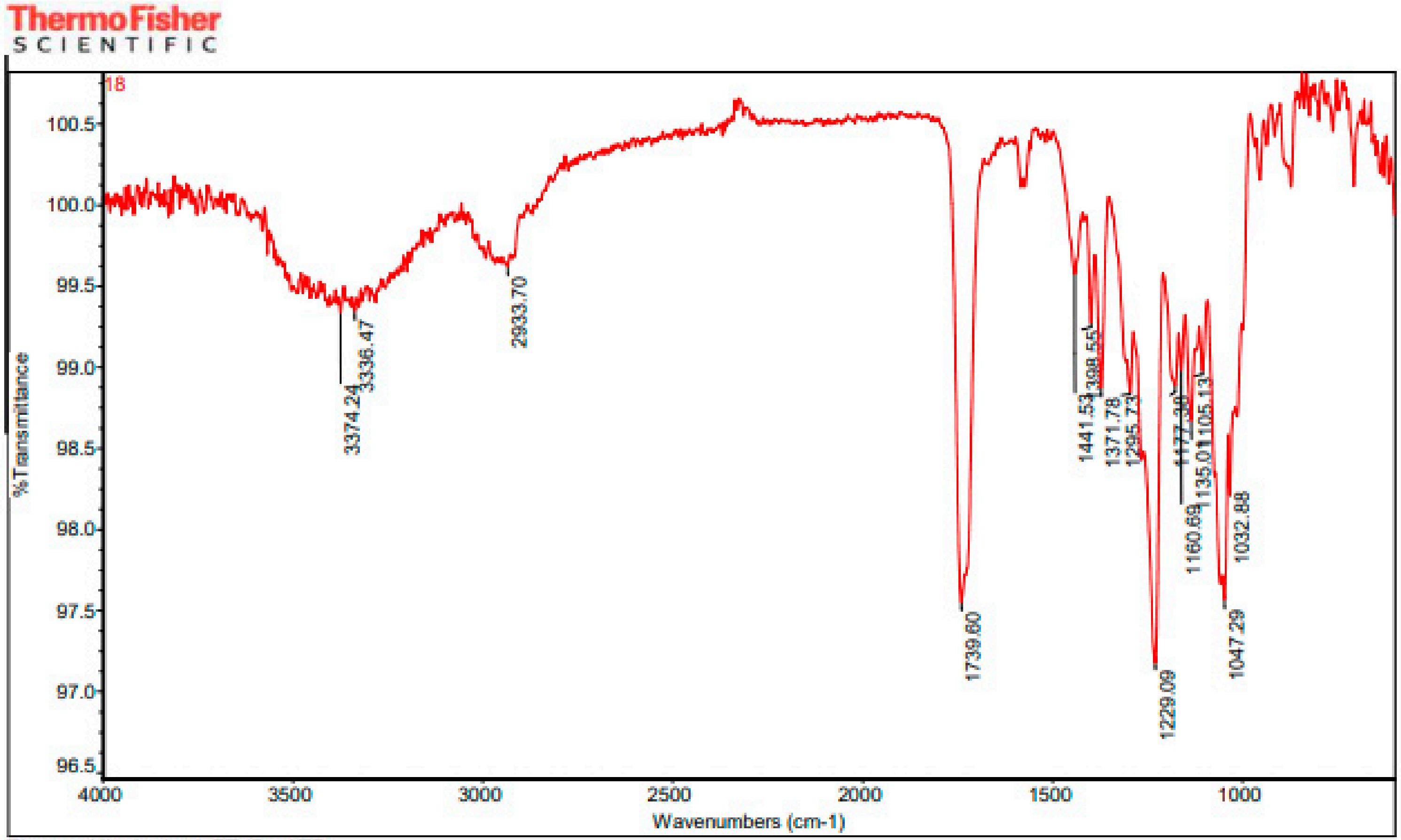

Thermo Nicolet is50 FT-IR ATR
18
Number of sample scans: 16
Number of background scans: 16
Resolution: 4.000
Sample gain: 8.0
Optical velocity: 0.4747
Aperture: 150.00

Figure S10. UV spectrum of **1**

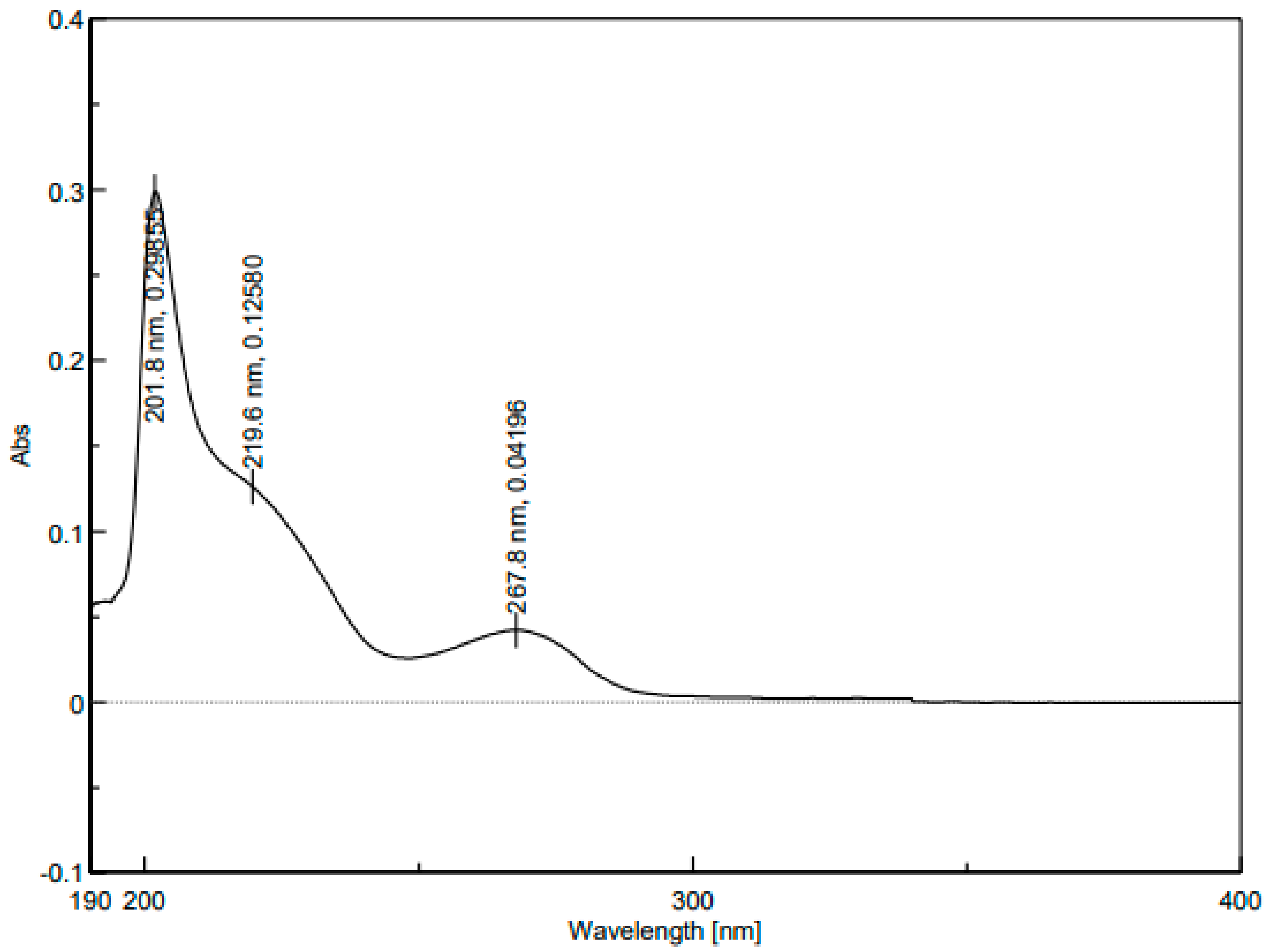

Figure S11. HR-ESI-MS spectrum of compound **1**

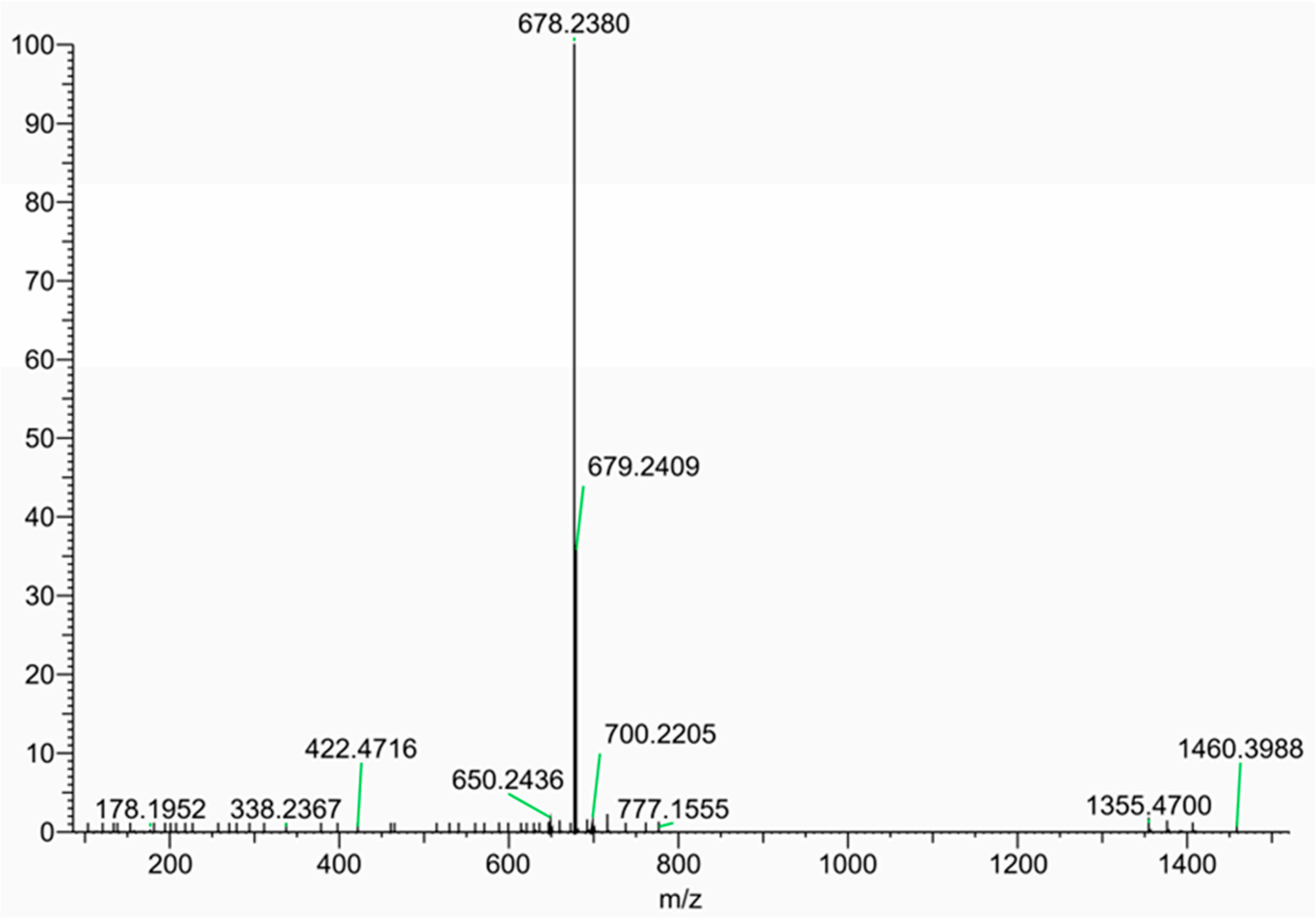

Figure S12. ^{1}H-NMR spectrum of compound **2** ($CDCl_3$, 600 MHz)

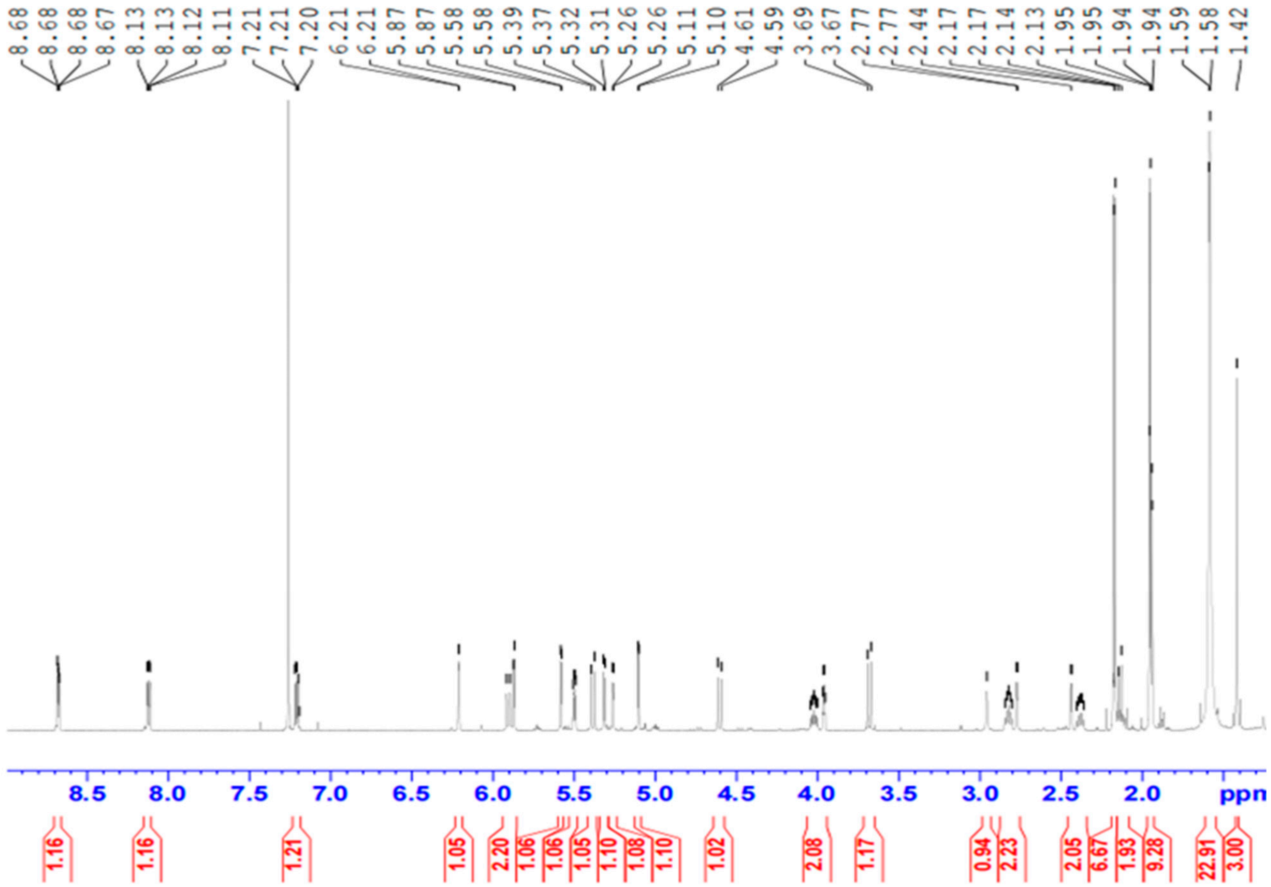

Figure S13. ^{13}C-NMR spectrum of compound **2** ($CDCl_3$, 150 MHz)

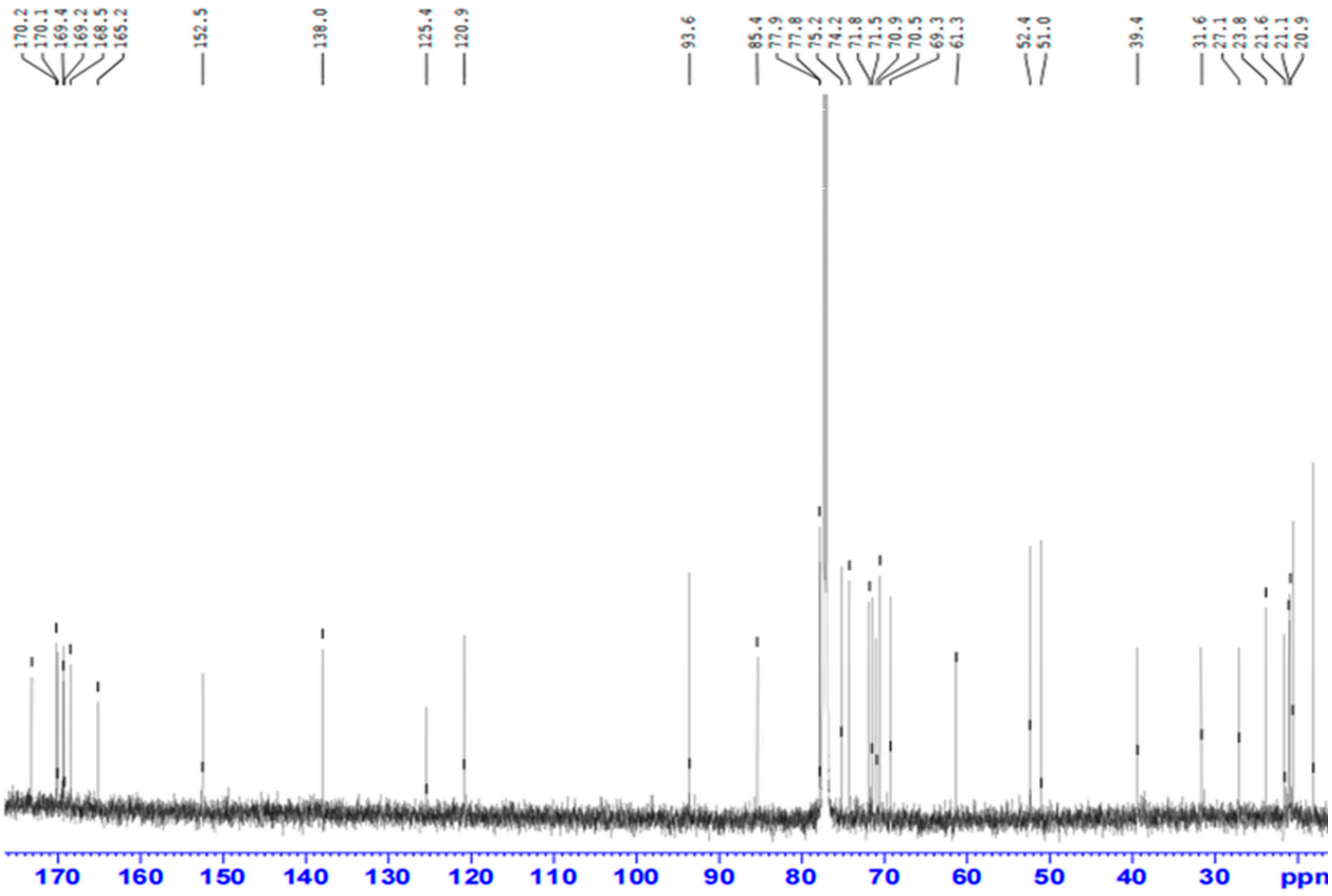

Figure S14. DEPT 135° spectrum of compound **2**

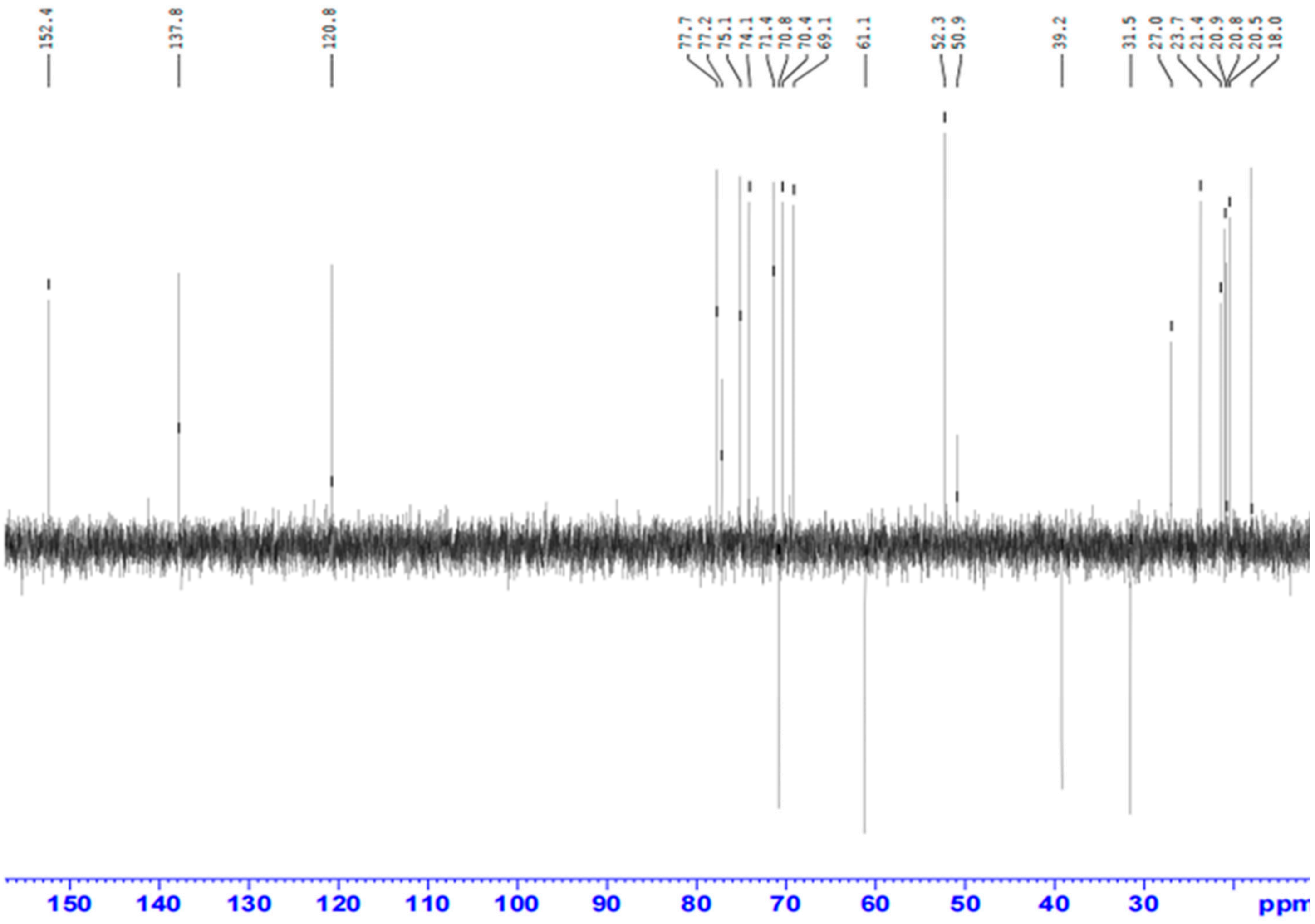

Figure S15. DEPT 90° spectrum of compound **2**

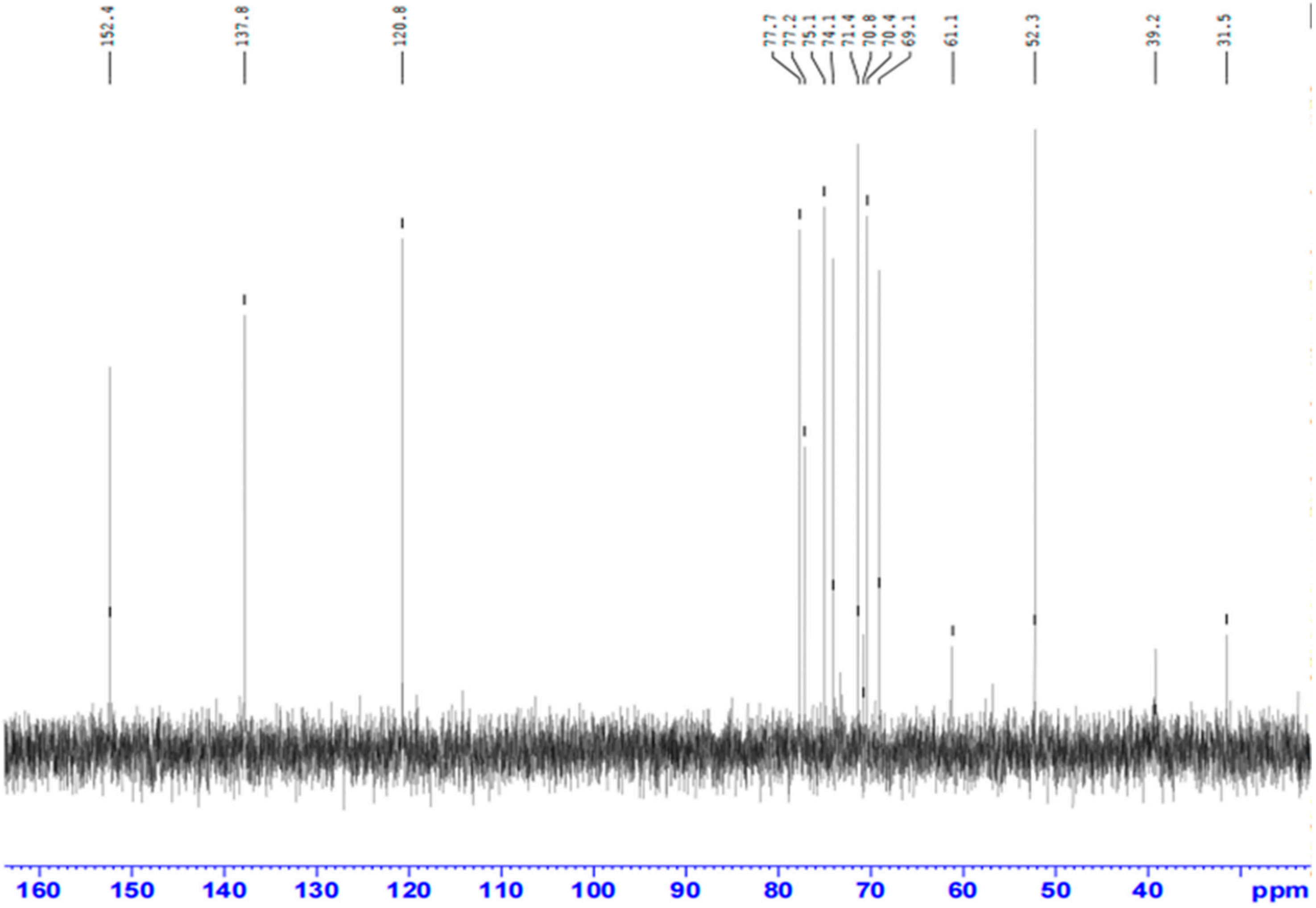

Figure S16. ^1H-^1H COSY spectrum of compound **2**

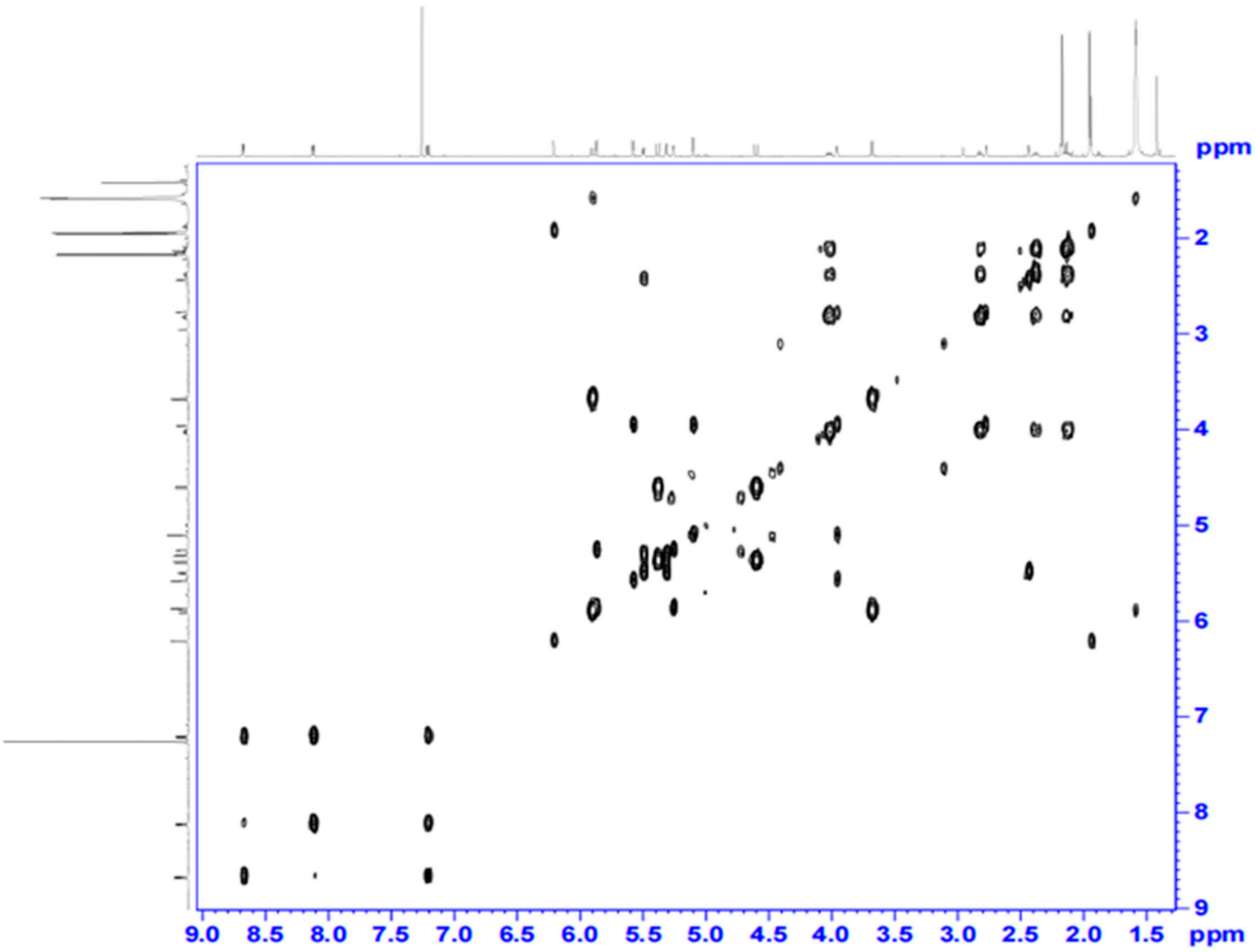

Figure S17. HSQC spectrum of compound **2**

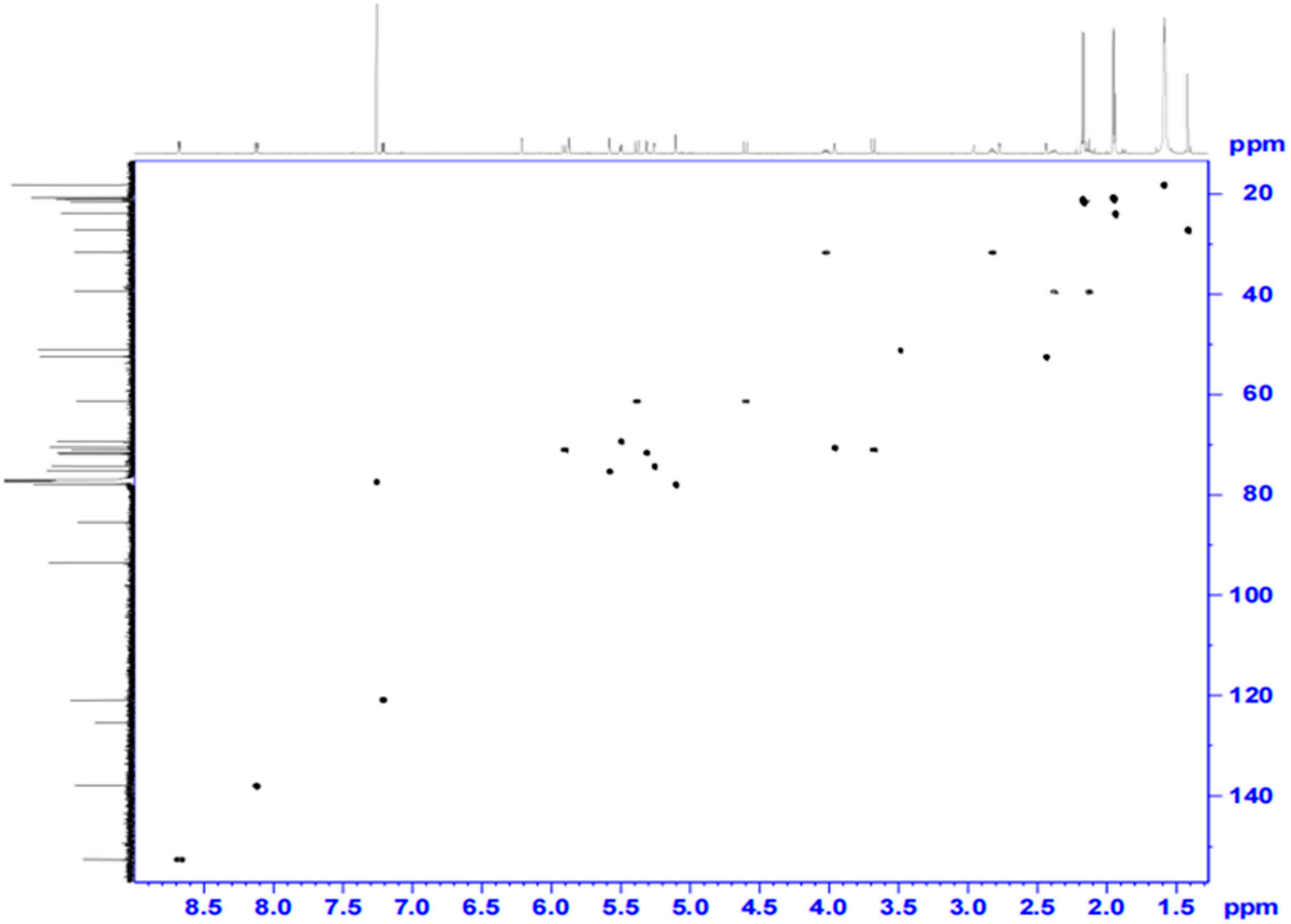

Figure S18. HMBC spectrum of compound **2**

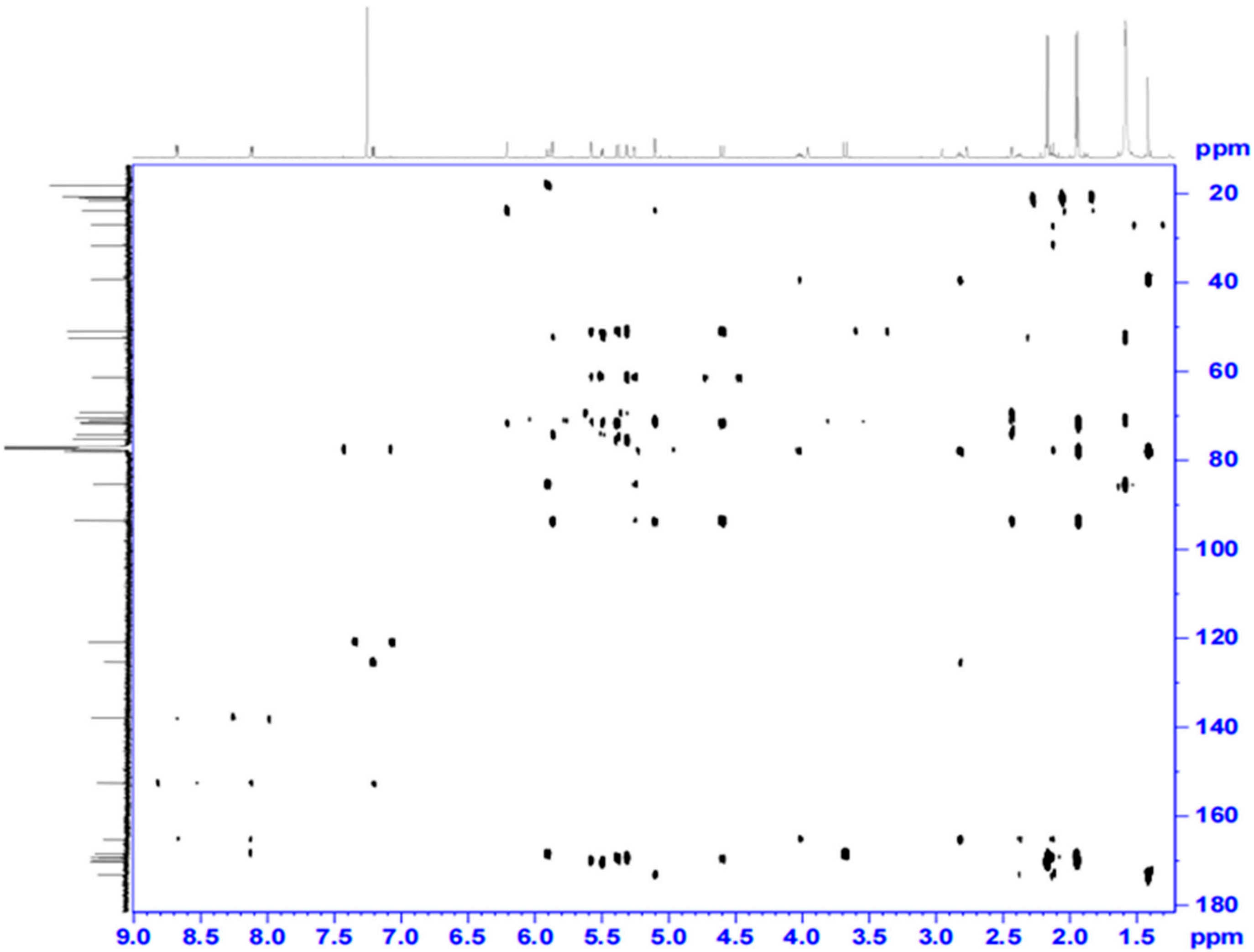

Figure S19. ROESY spectrum of compound **2**

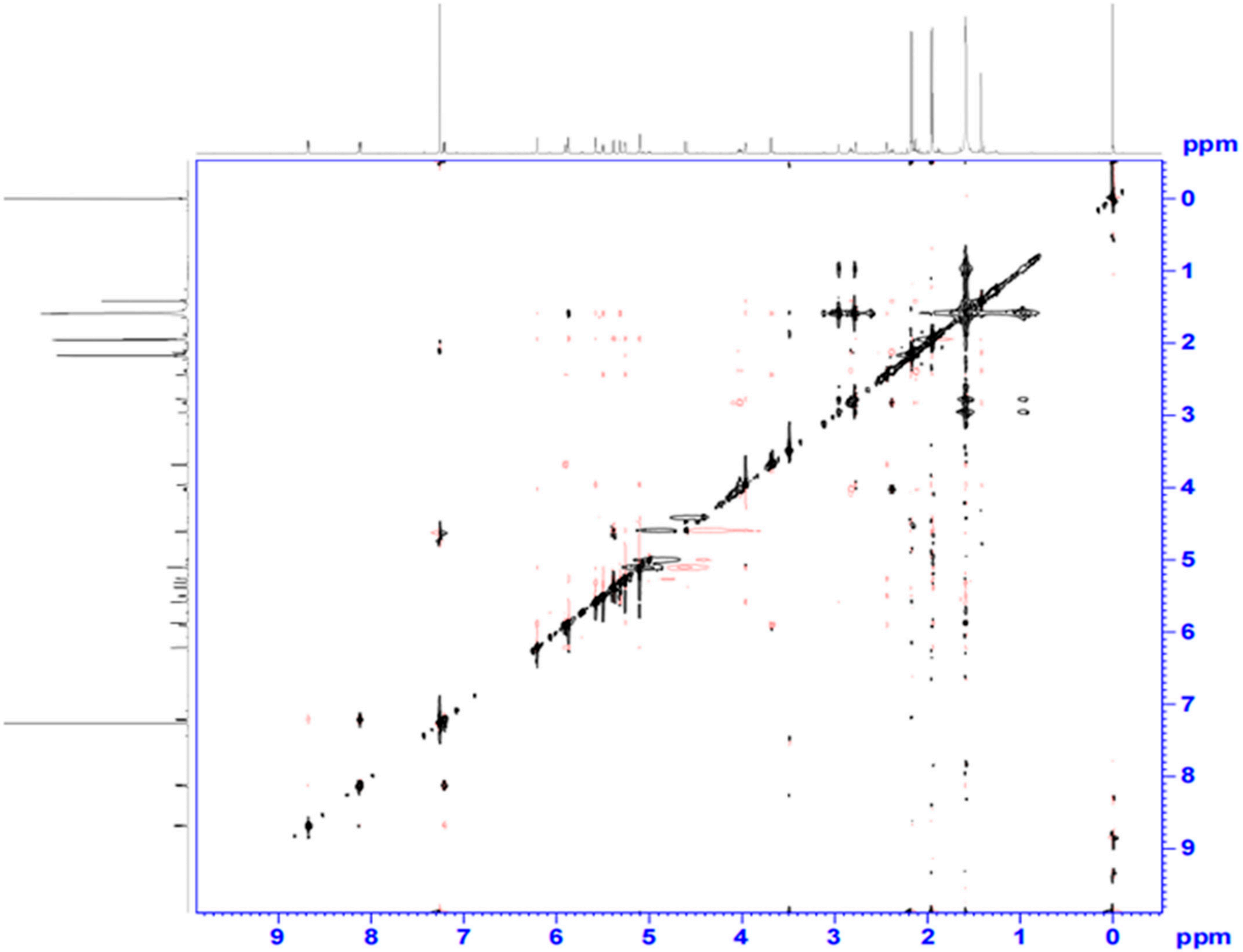

Figure S20. IR spectrum of **2**

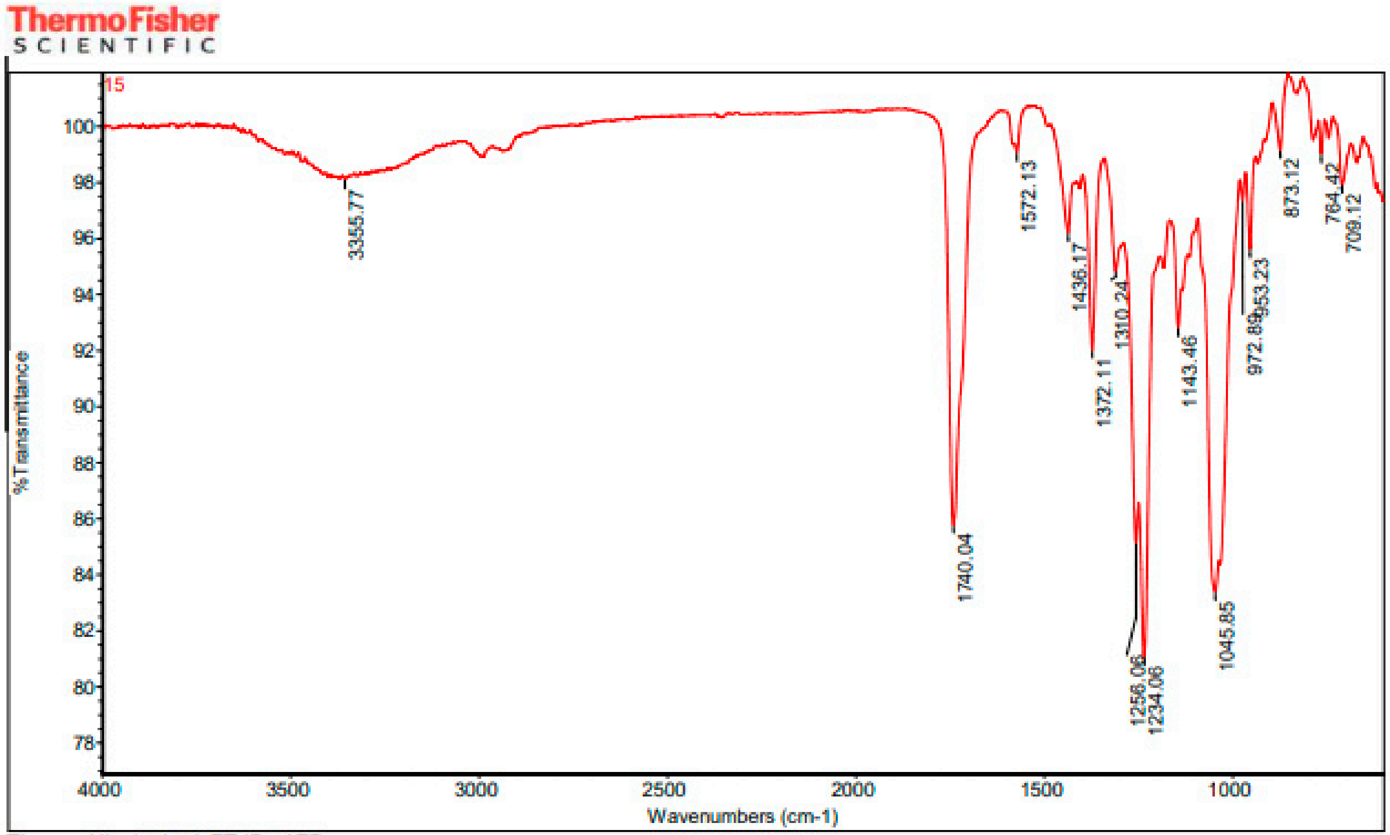

Thermo Nicolet is50 FT-IR ATR
15
Number of sample scans: 16
Number of background scans: 16
Resolution: 4.000
Sample gain: 8.0
Optical velocity: 0.4747
Aperture: 150.00

Figure S21. UV spectrum of **2**

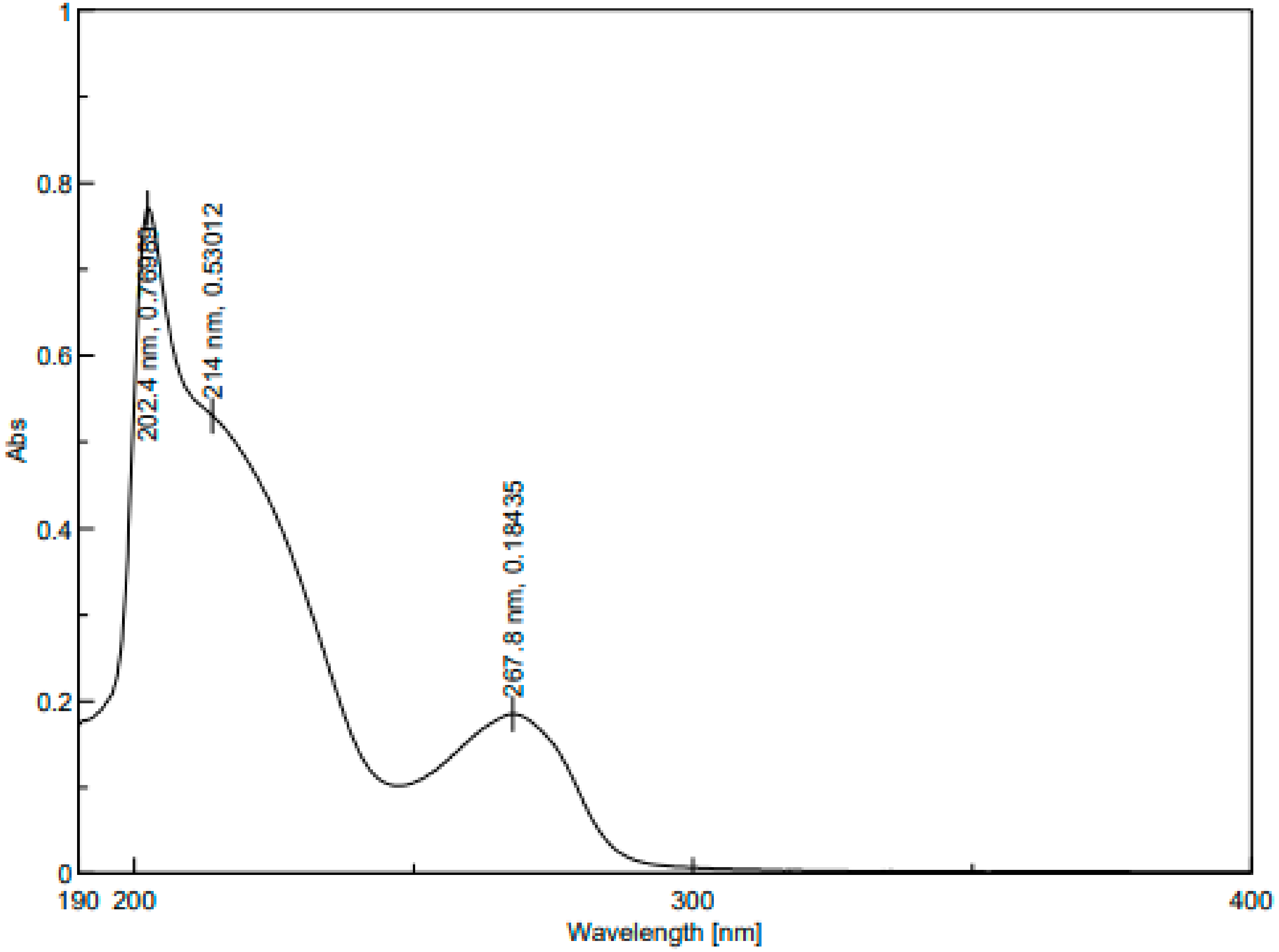

Figure S22. HR-ESI-MS spectrum of compound **2**

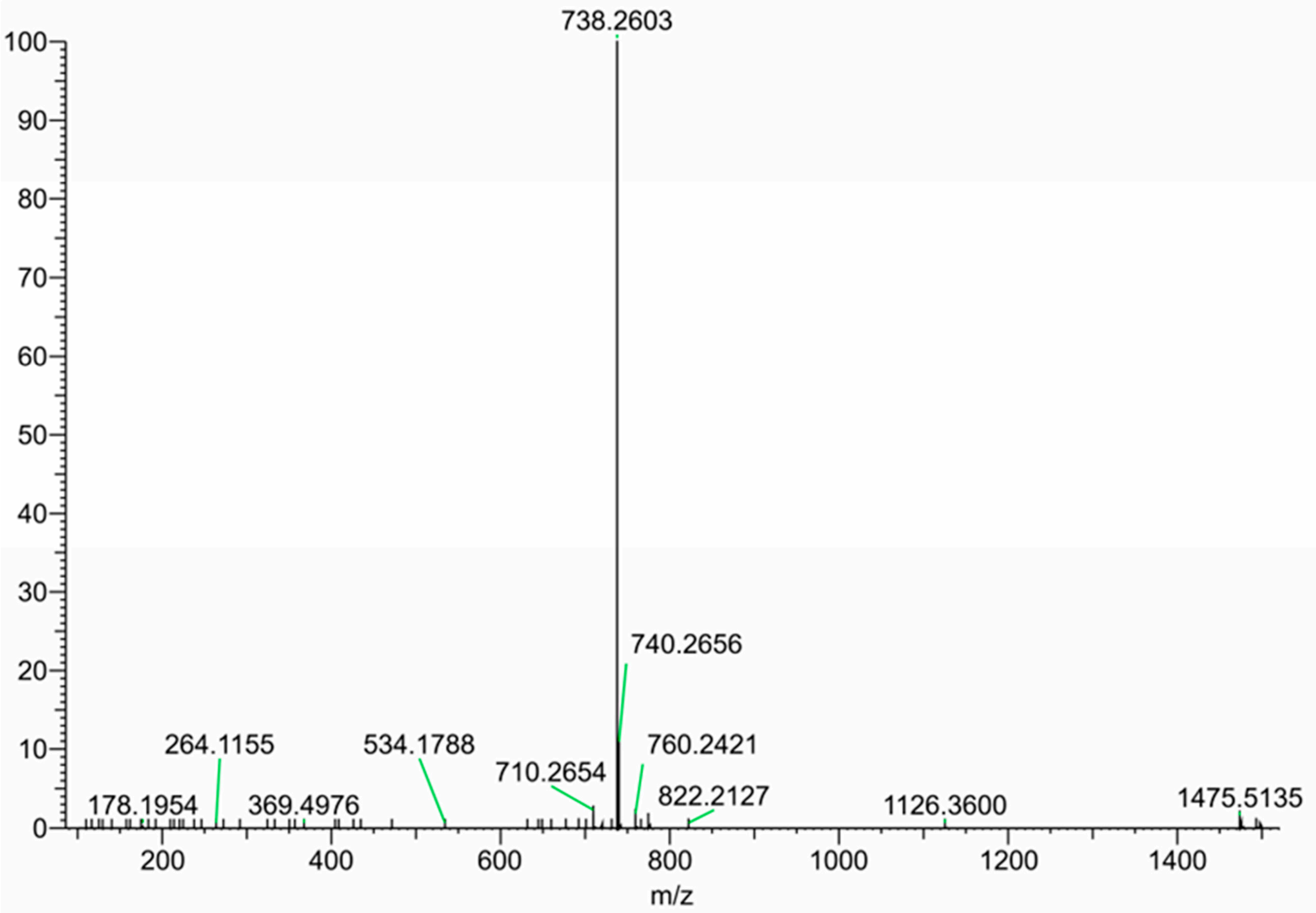

Figure S23. ¹H-NMR spectrum of compound **3** ($CDCl_3$, 600 MHz)

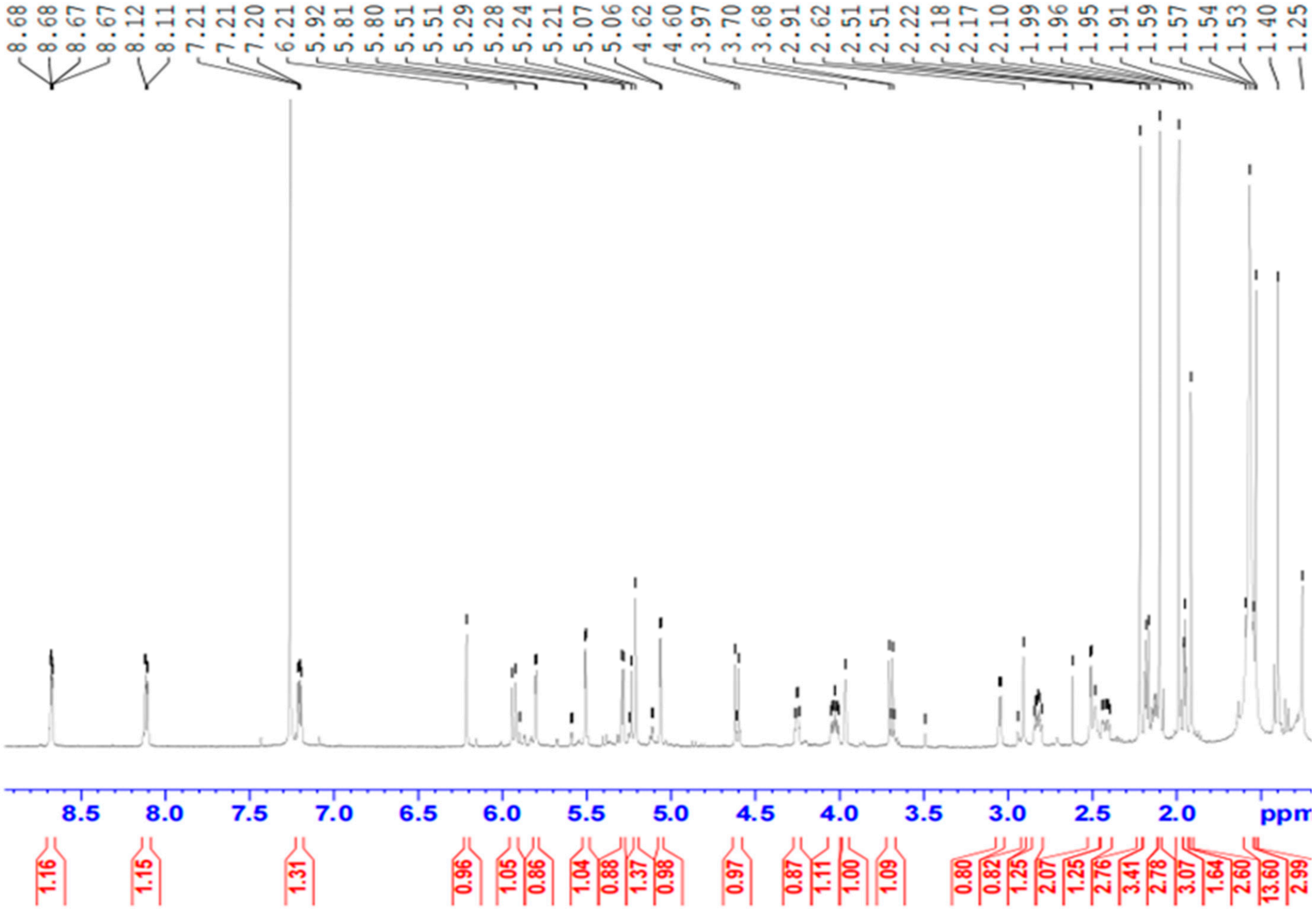

Figure S24. ^{13}C-NMR spectrum of compound **3** ($CDCl_3$, 150 MHz)

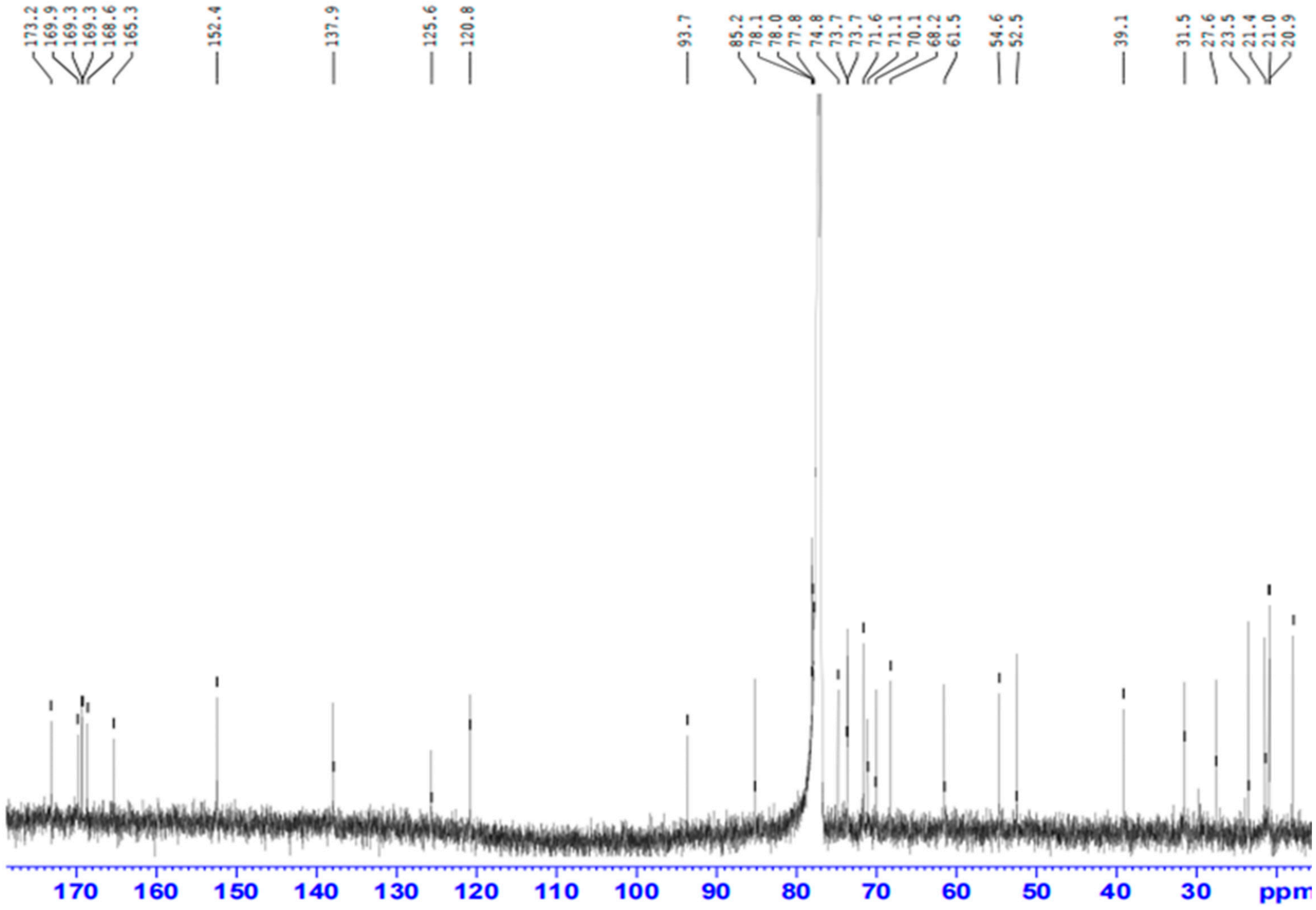

Figure S25. DEPT 135° spectrum of compound **3**

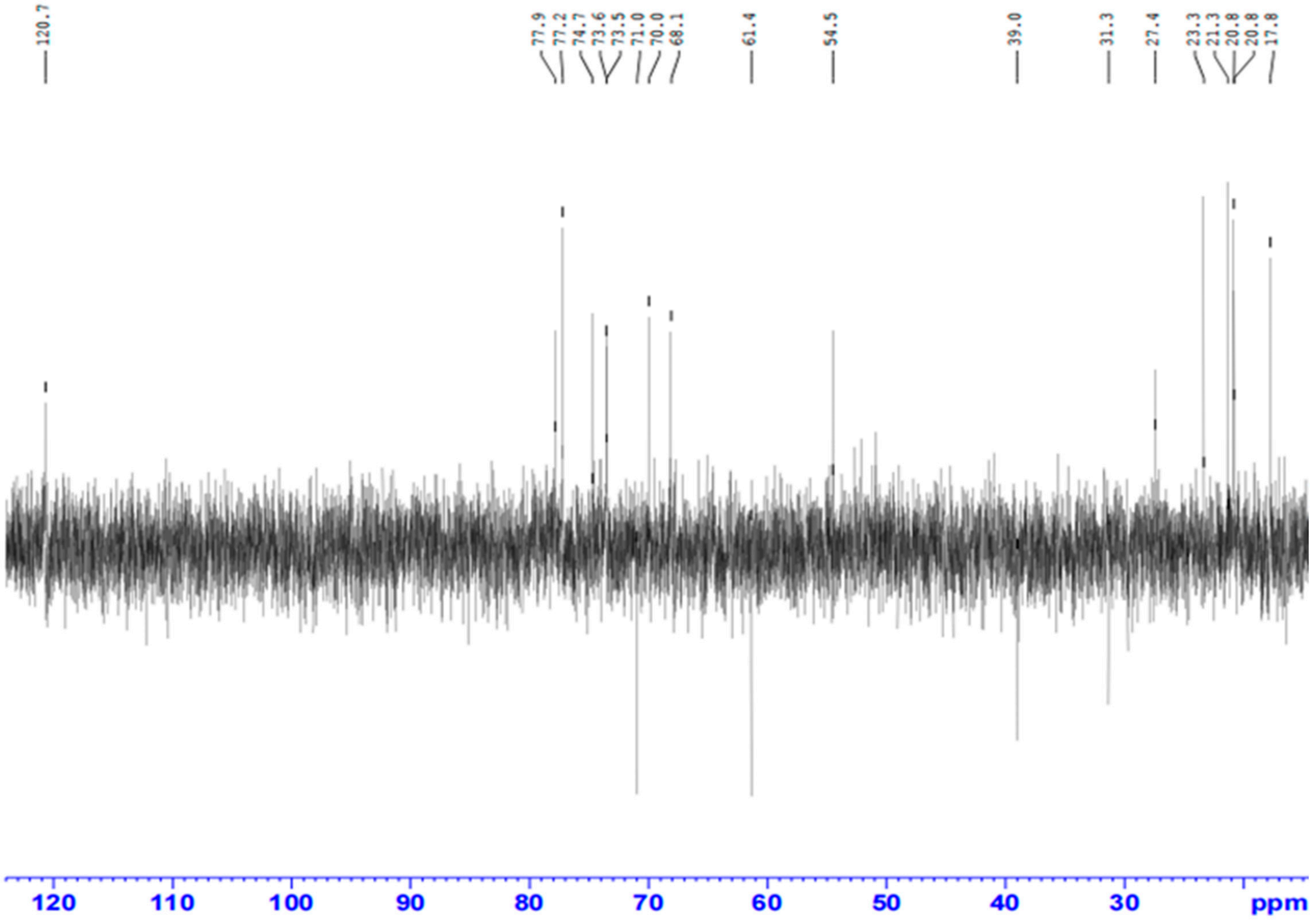

Figure S26. DEPT 90° spectrum of compound **3**

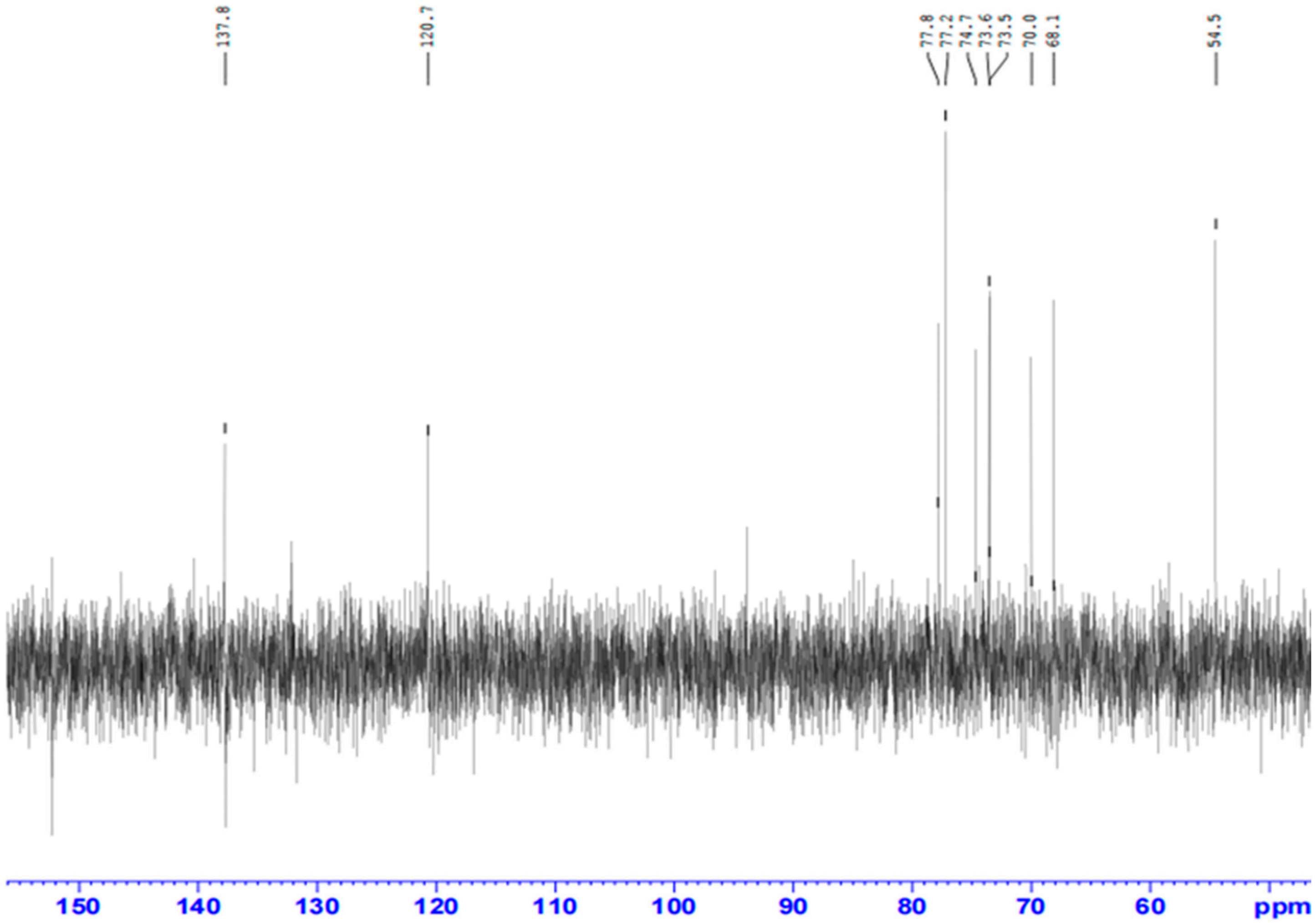

Figure S27. ^{1}H-^{1}H COSY spectrum of compound **3**

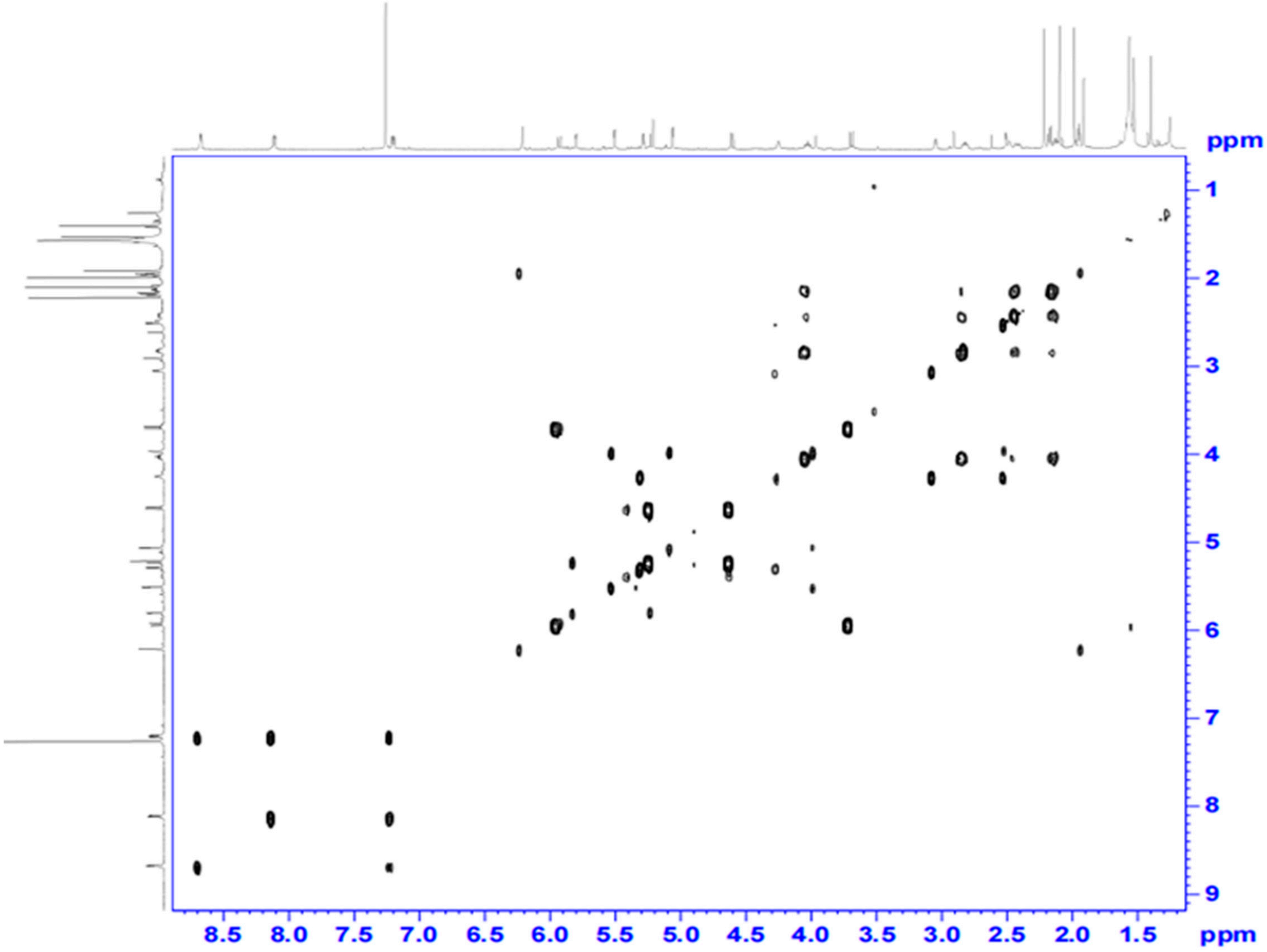

Figure S28. HSQC spectrum of compound **3**

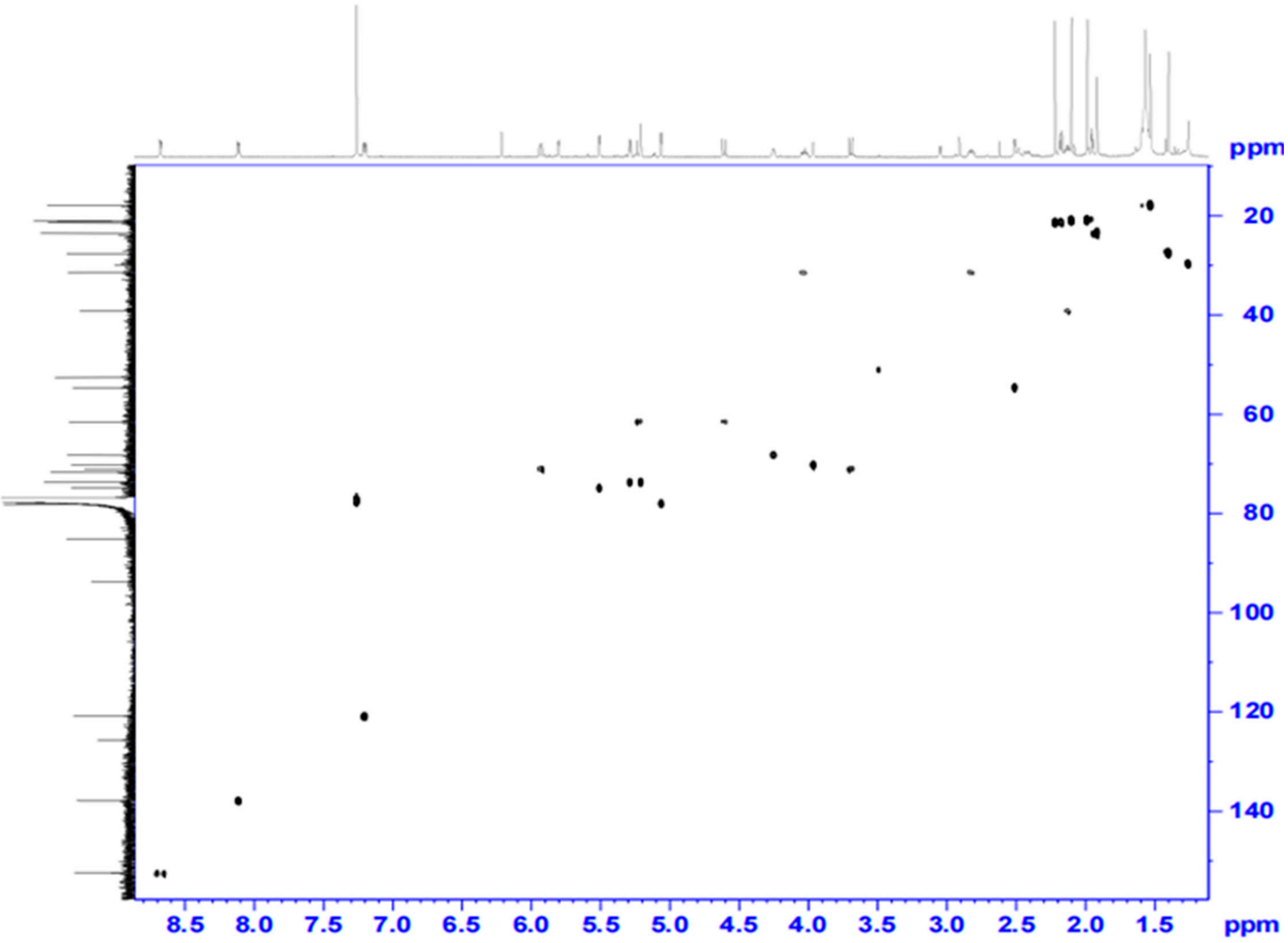

Figure S29. HMBC spectrum of compound **30**

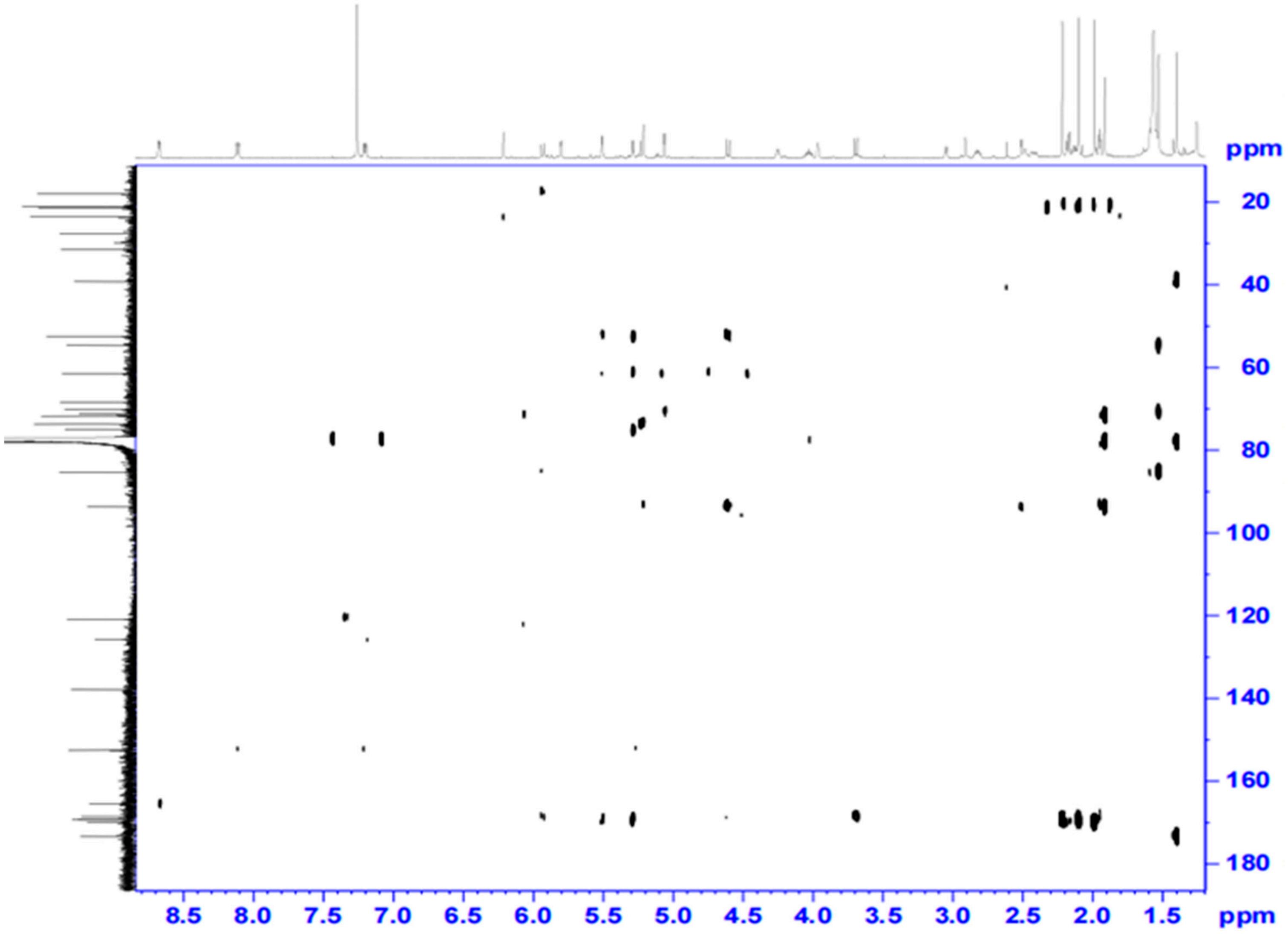

Figure S30. ROESY spectrum of compound **3**

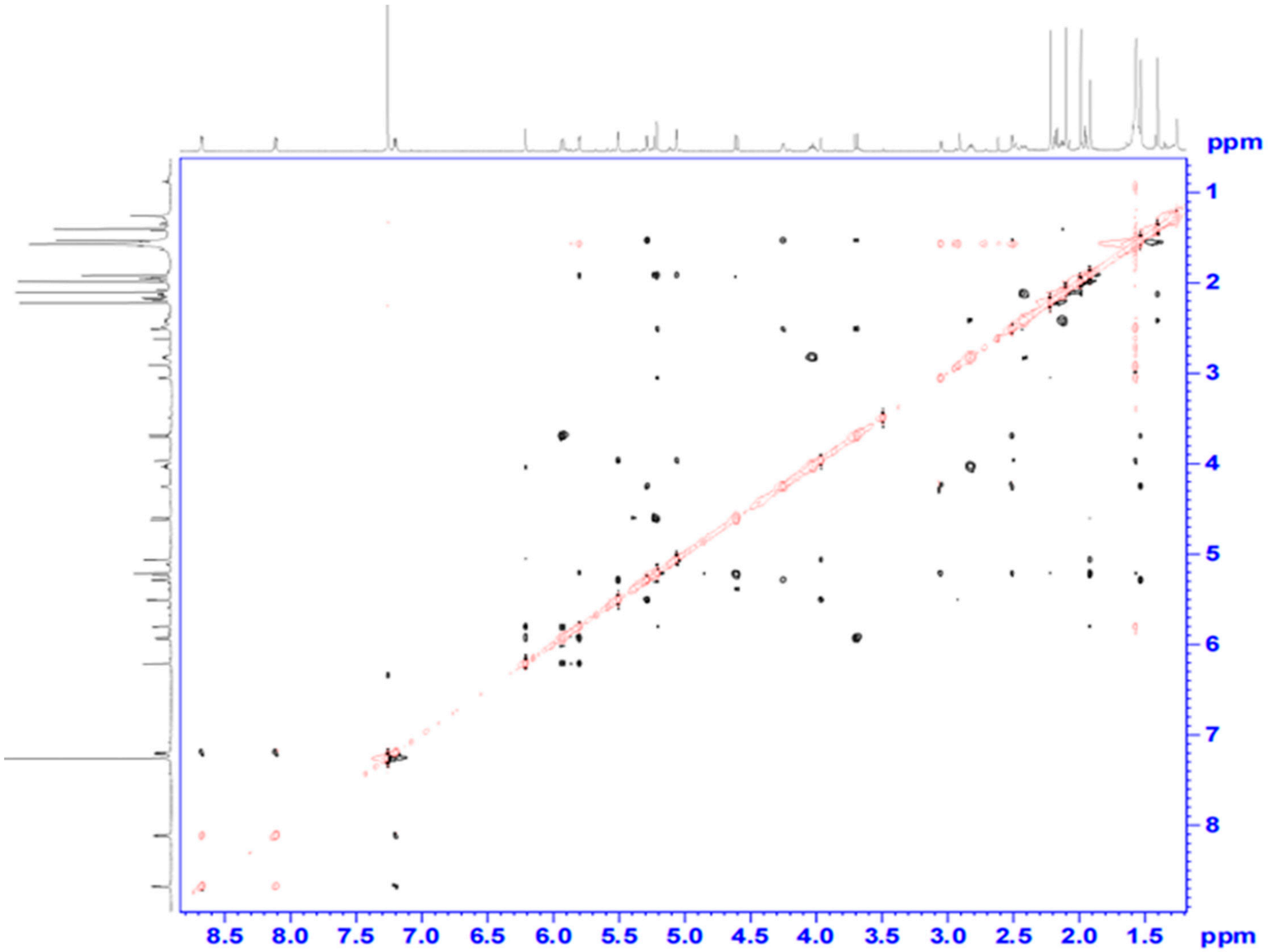

Figure S31. IR spectrum of **3**

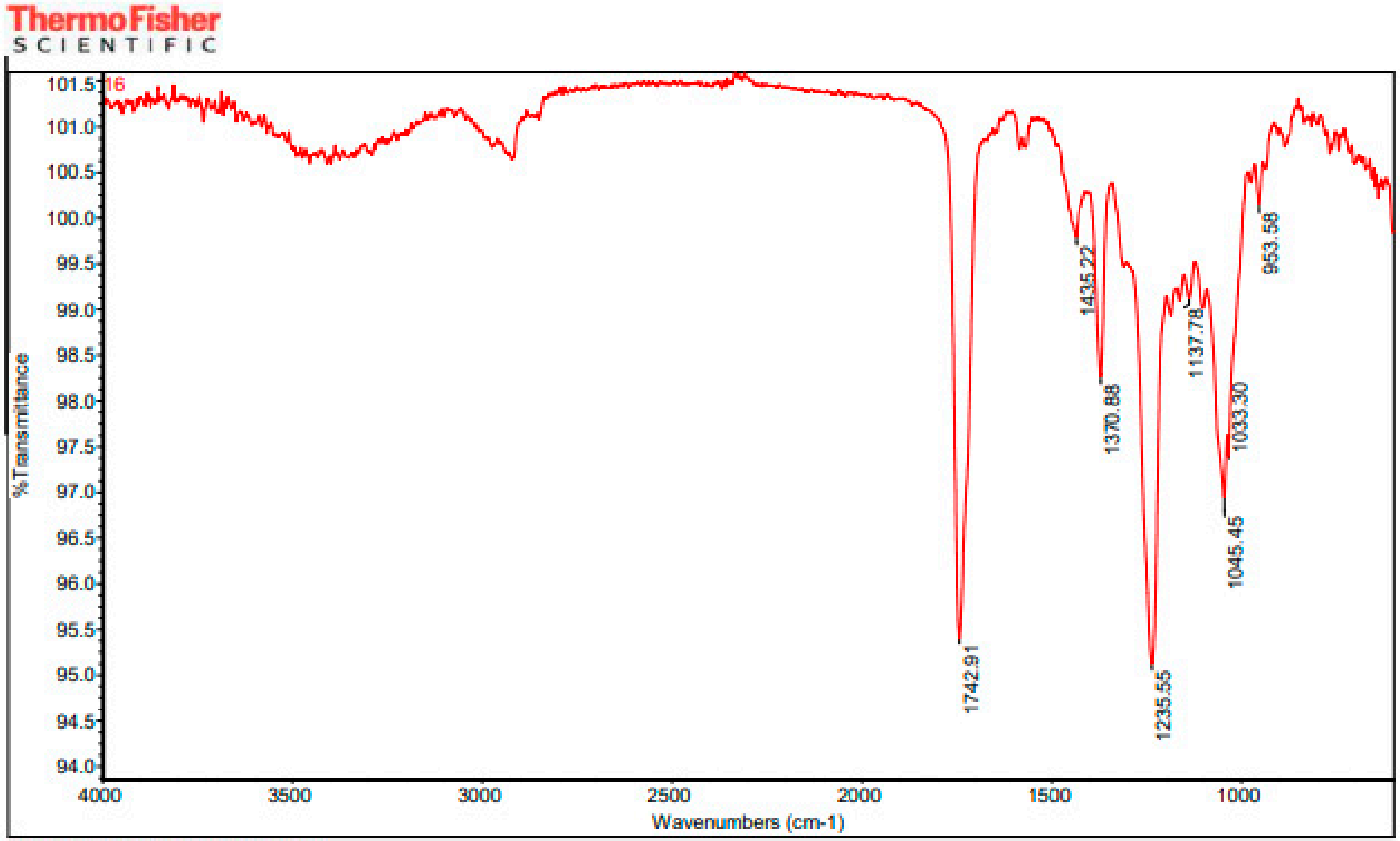

Thermo Nicolet is50 FT-IR ATR
16
Number of sample scans: 16
Number of background scans: 16
Resolution: 4.000
Sample gain: 8.0
Optical velocity: 0.4747
Aperture: 150.00

Figure S32. UV spectrum of **3**

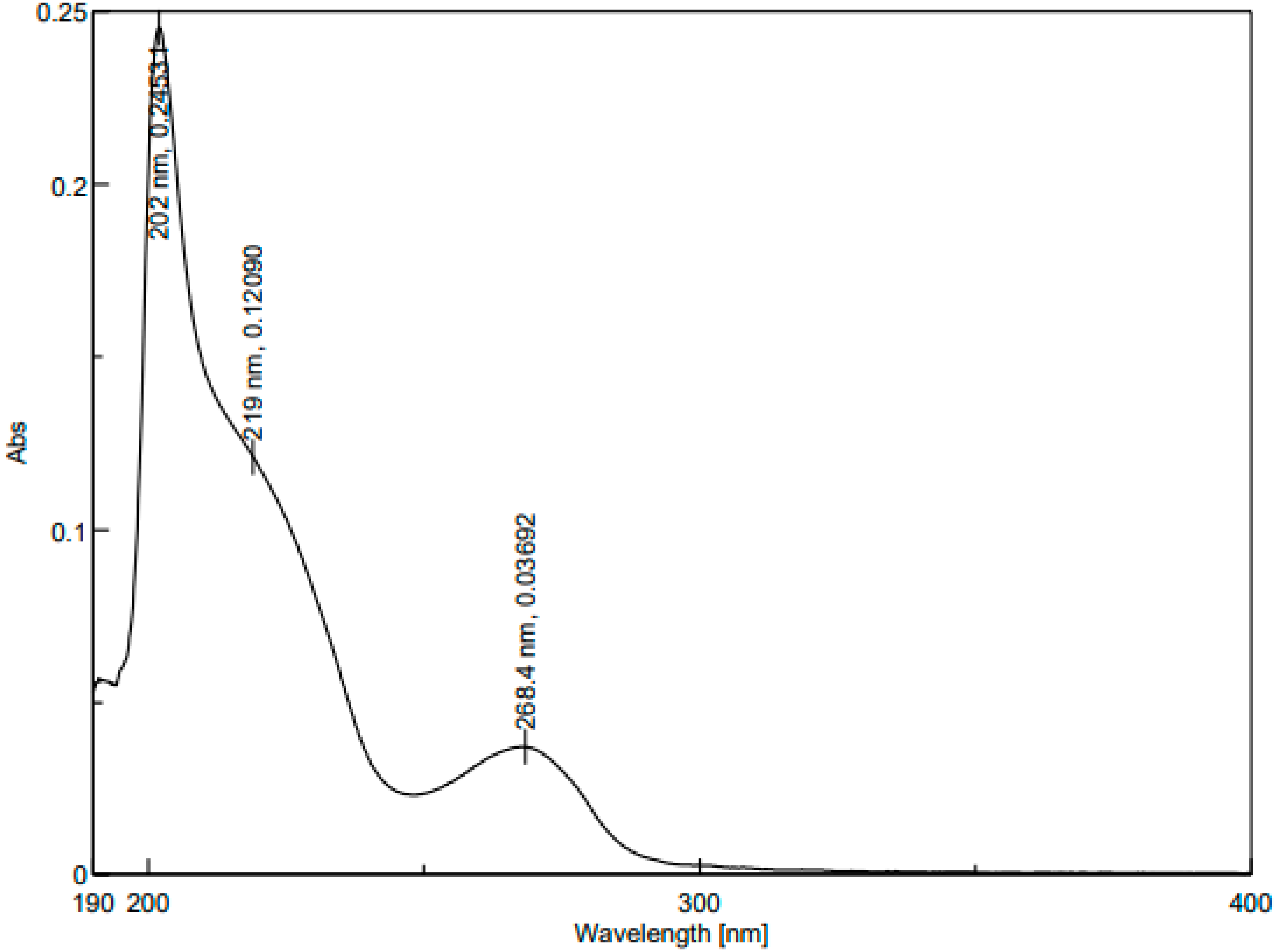

Figure S33. HR-ESI-MS spectrum of compound **3**

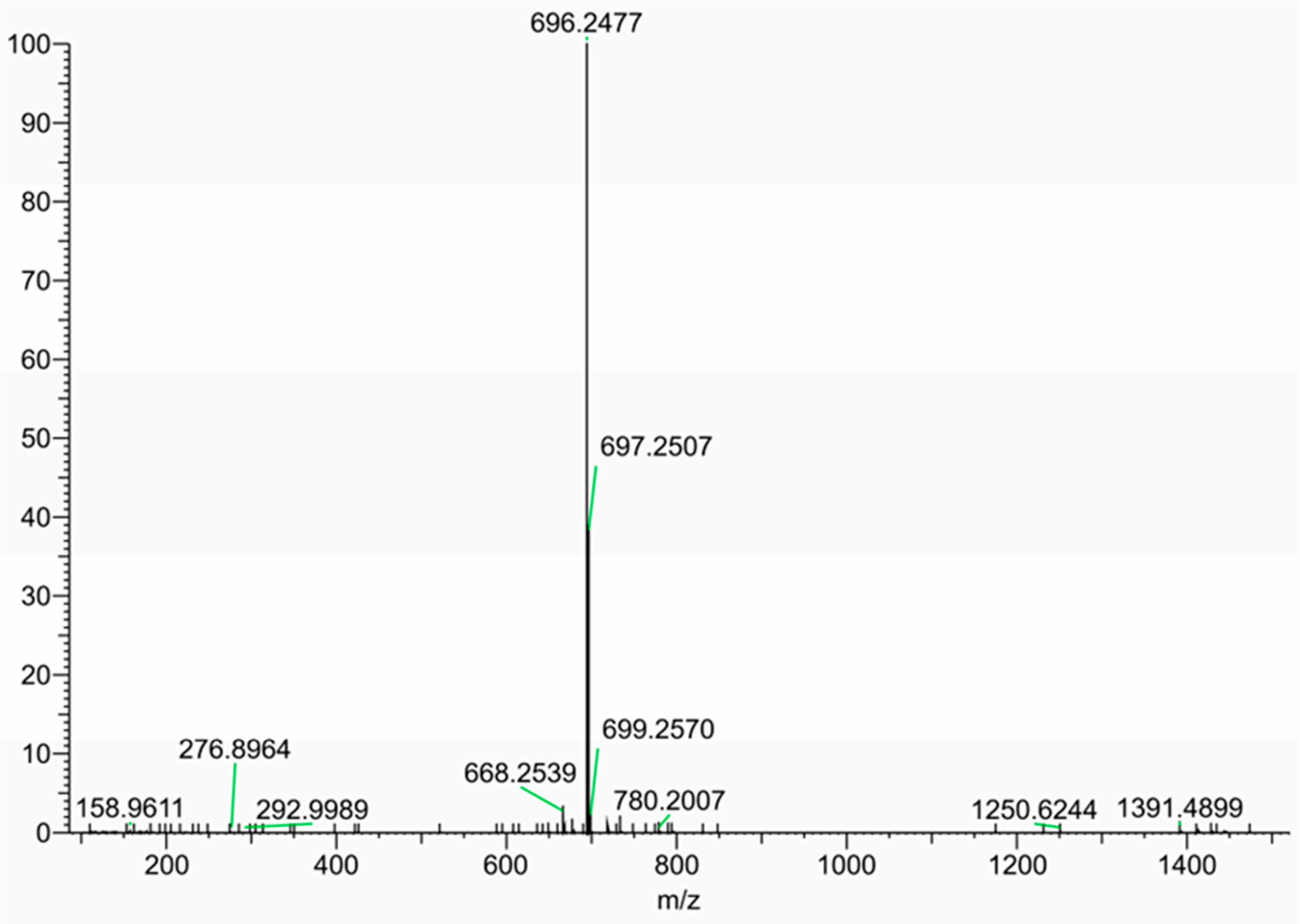

Figure S34. ^{1}H-NMR spectrum of compound **4** ($CDCl_3$, 600 MHz)

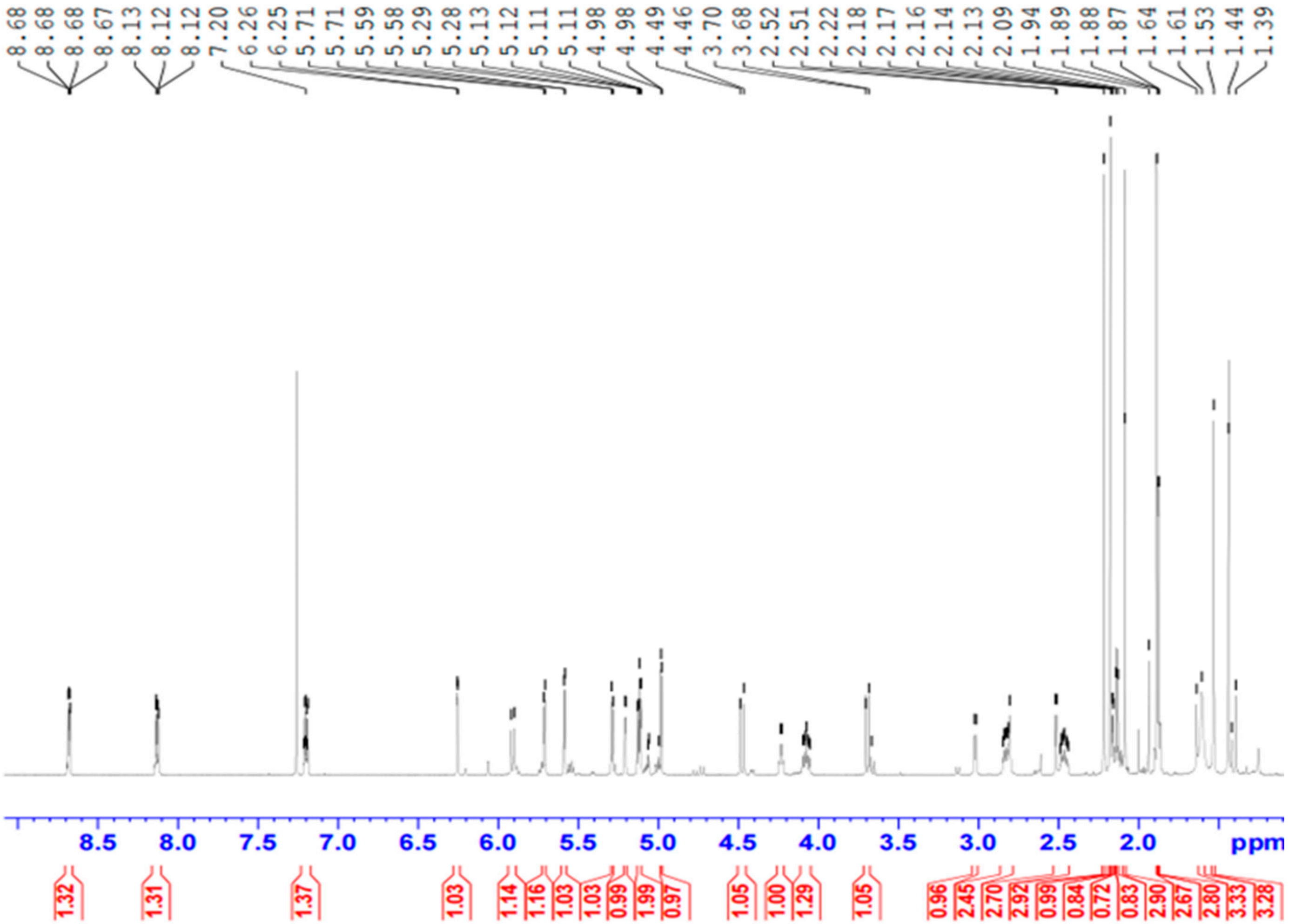

Figure S35. ^{13}C-NMR spectrum of compound **4** ($CDCl_3$, 150 MHz)

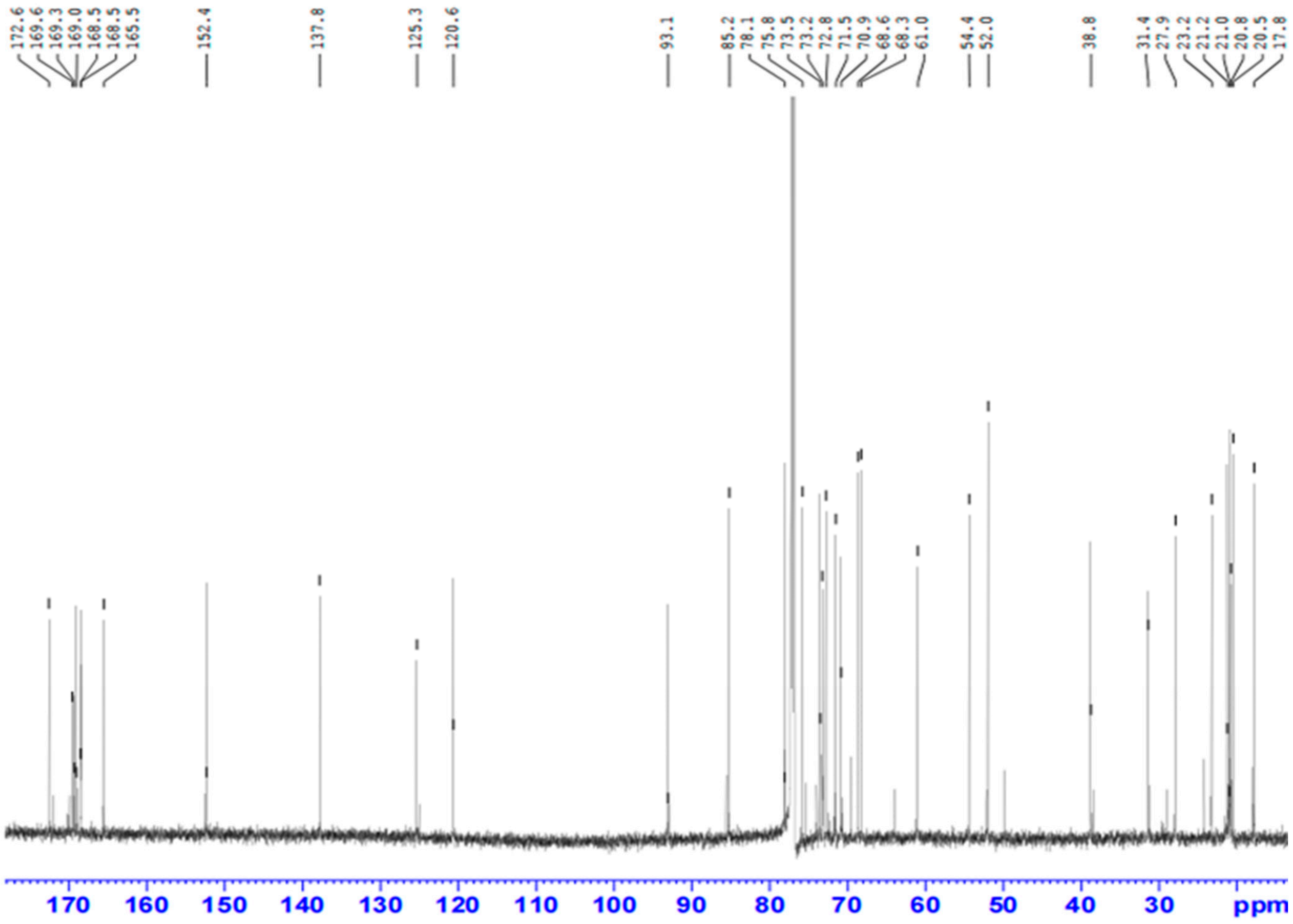

Figure S36. DEPT 135° spectrum of compound **4**

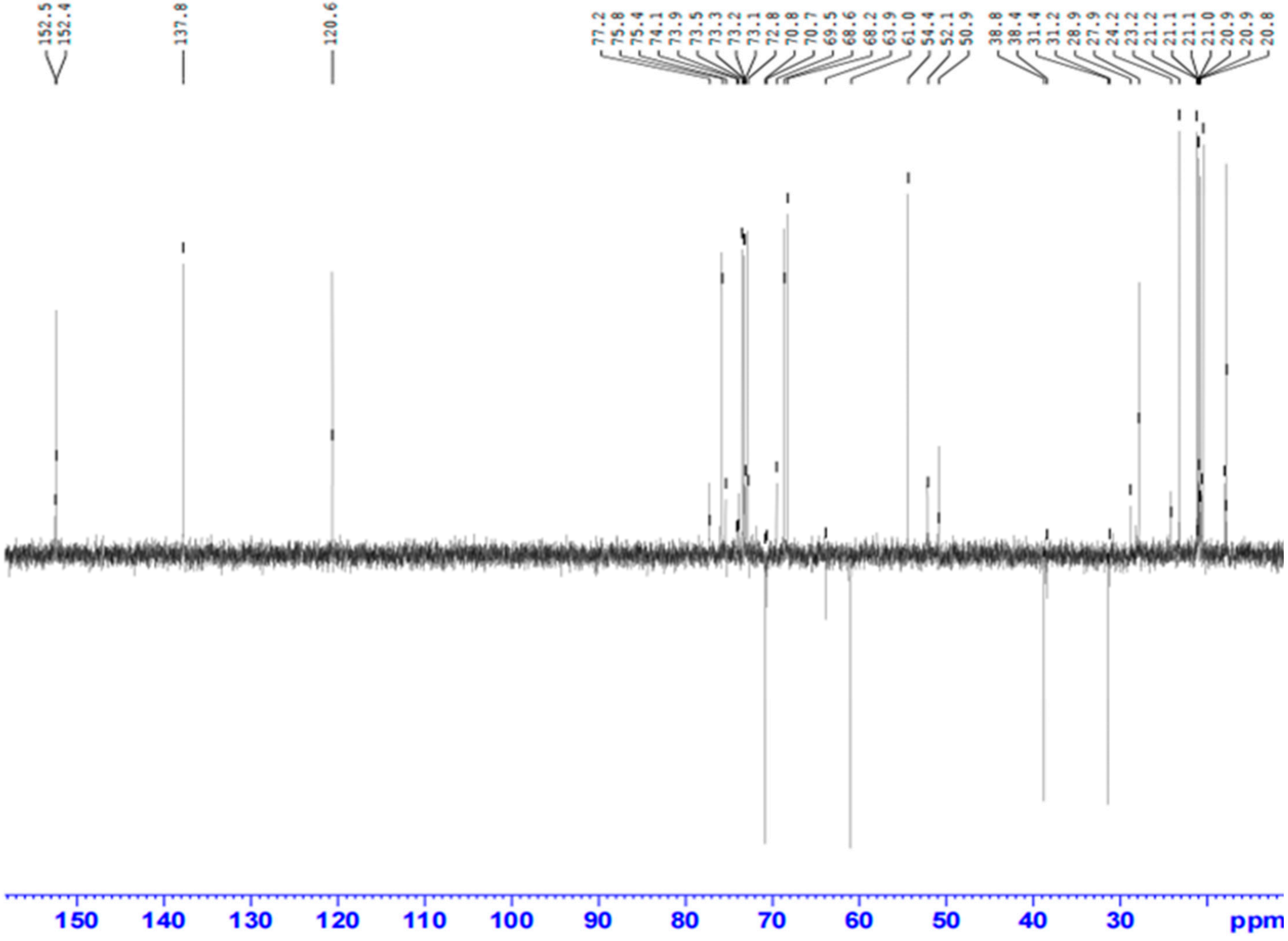

Figure S37. DEPT 90° spectrum of compound **4**

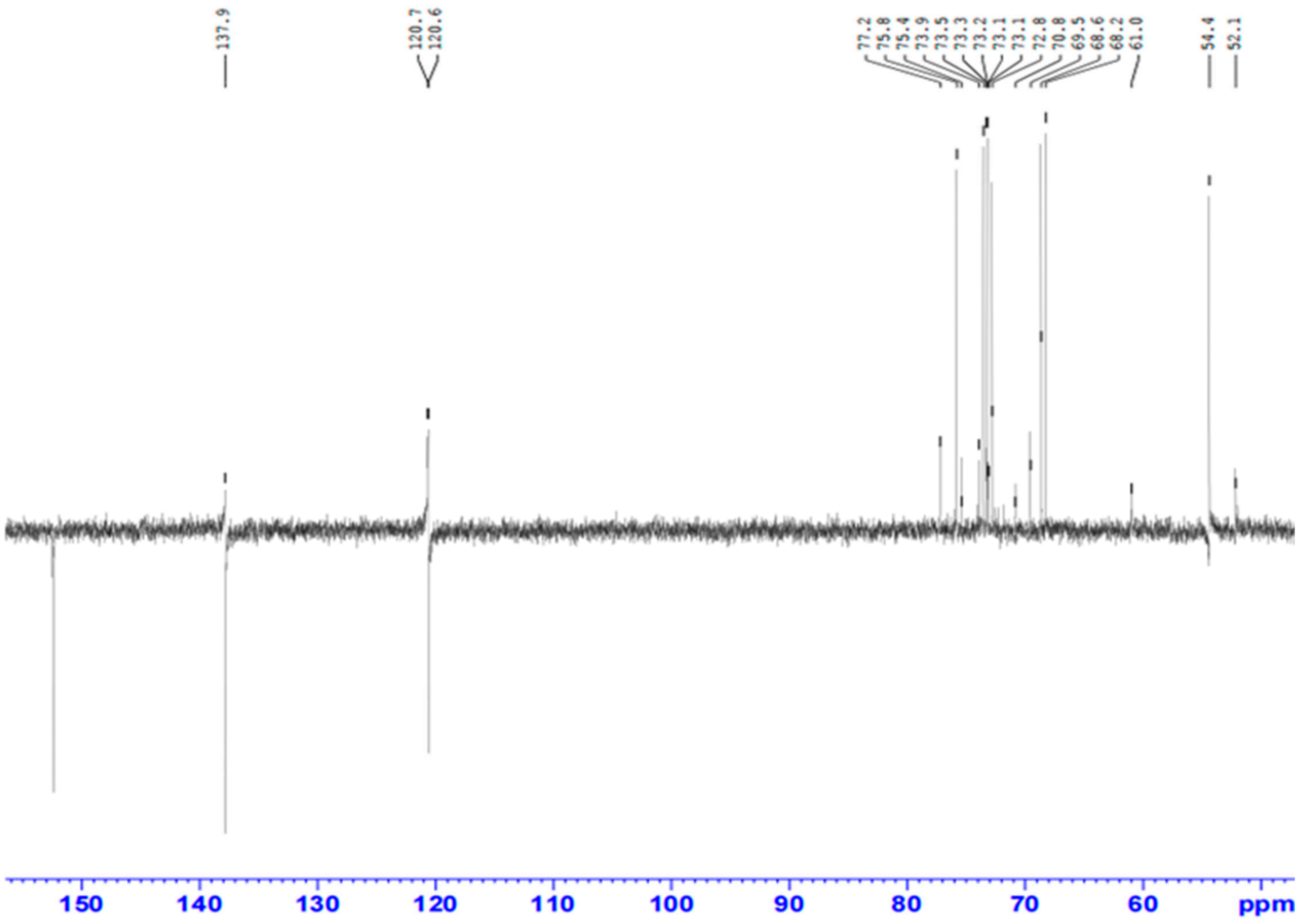

Figure S38. ^1H-^1H COSY spectrum of compound **4**

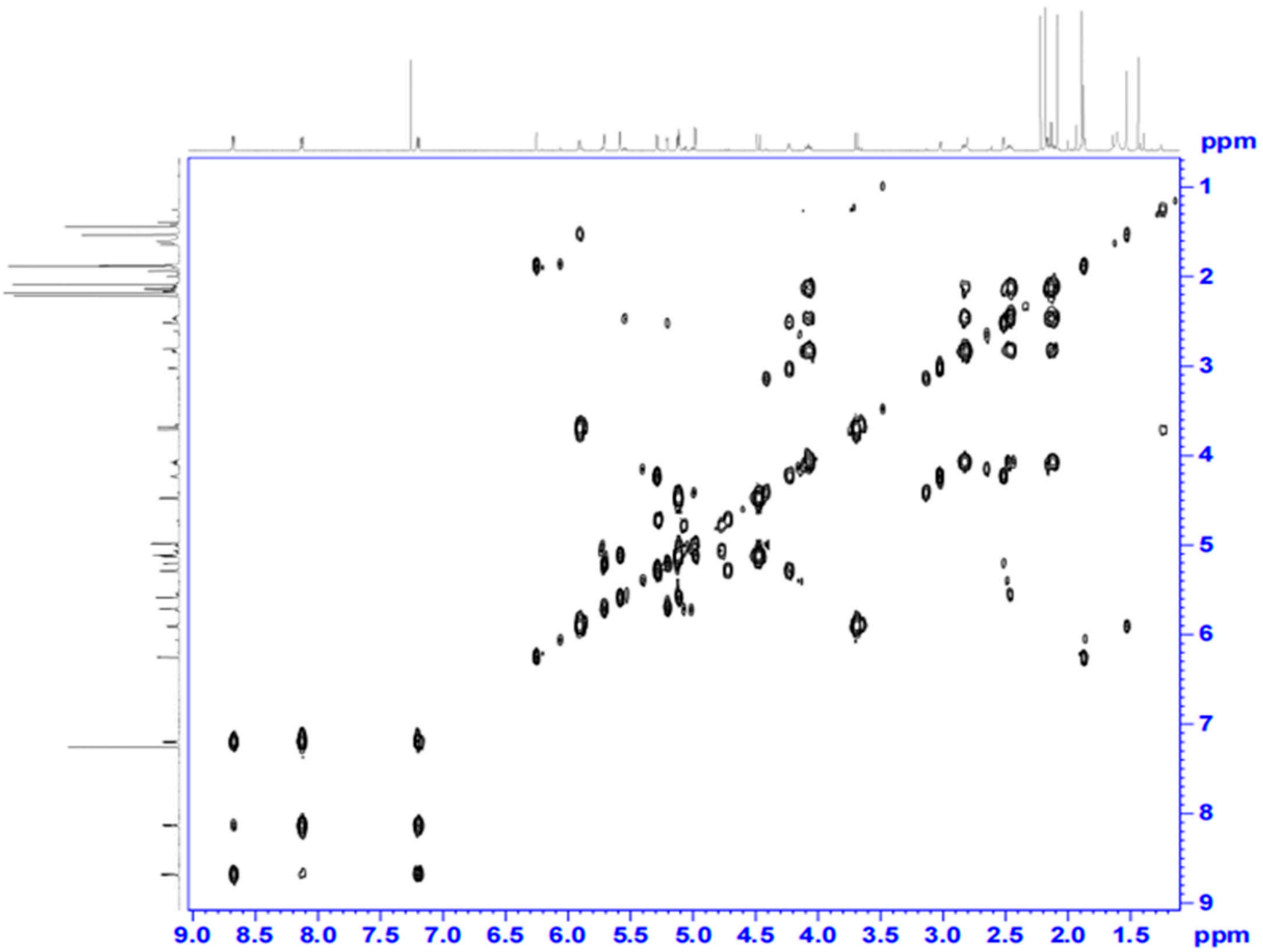

Figure S39. HSQC spectrum of compound **4**

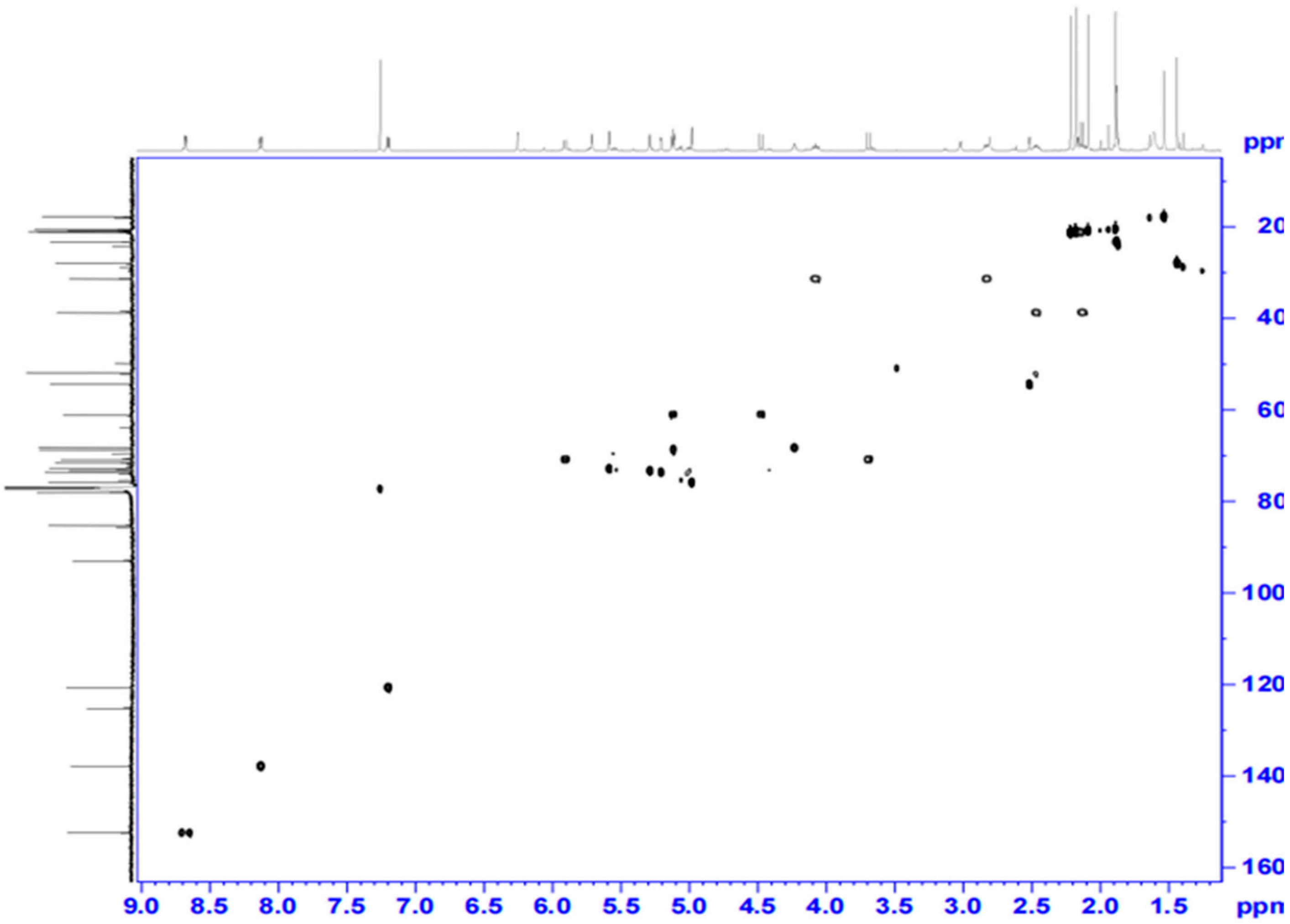

Figure S40. HMBC spectrum of compound **4**

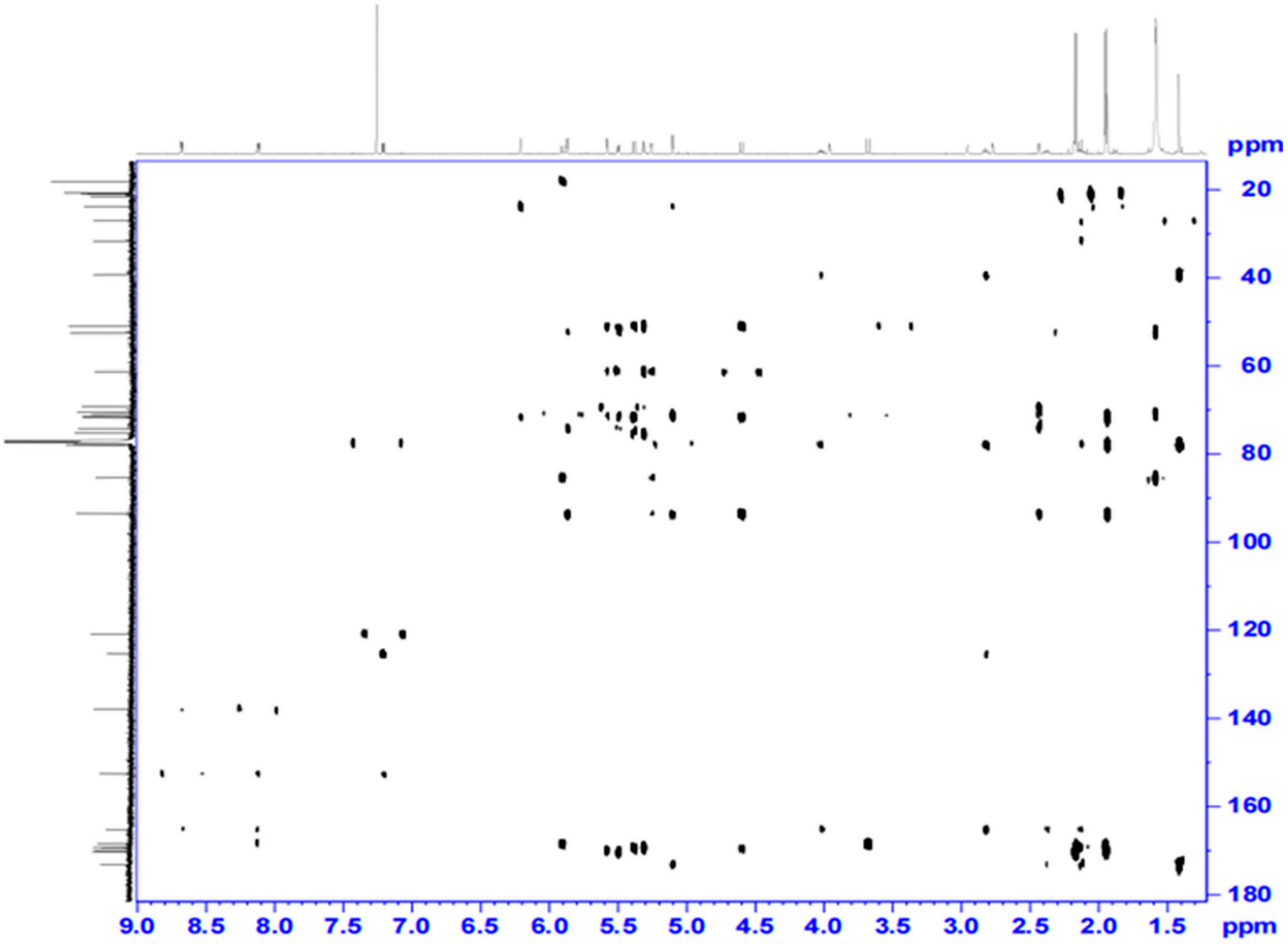

Figure S41. ROESY spectrum of compound **4**

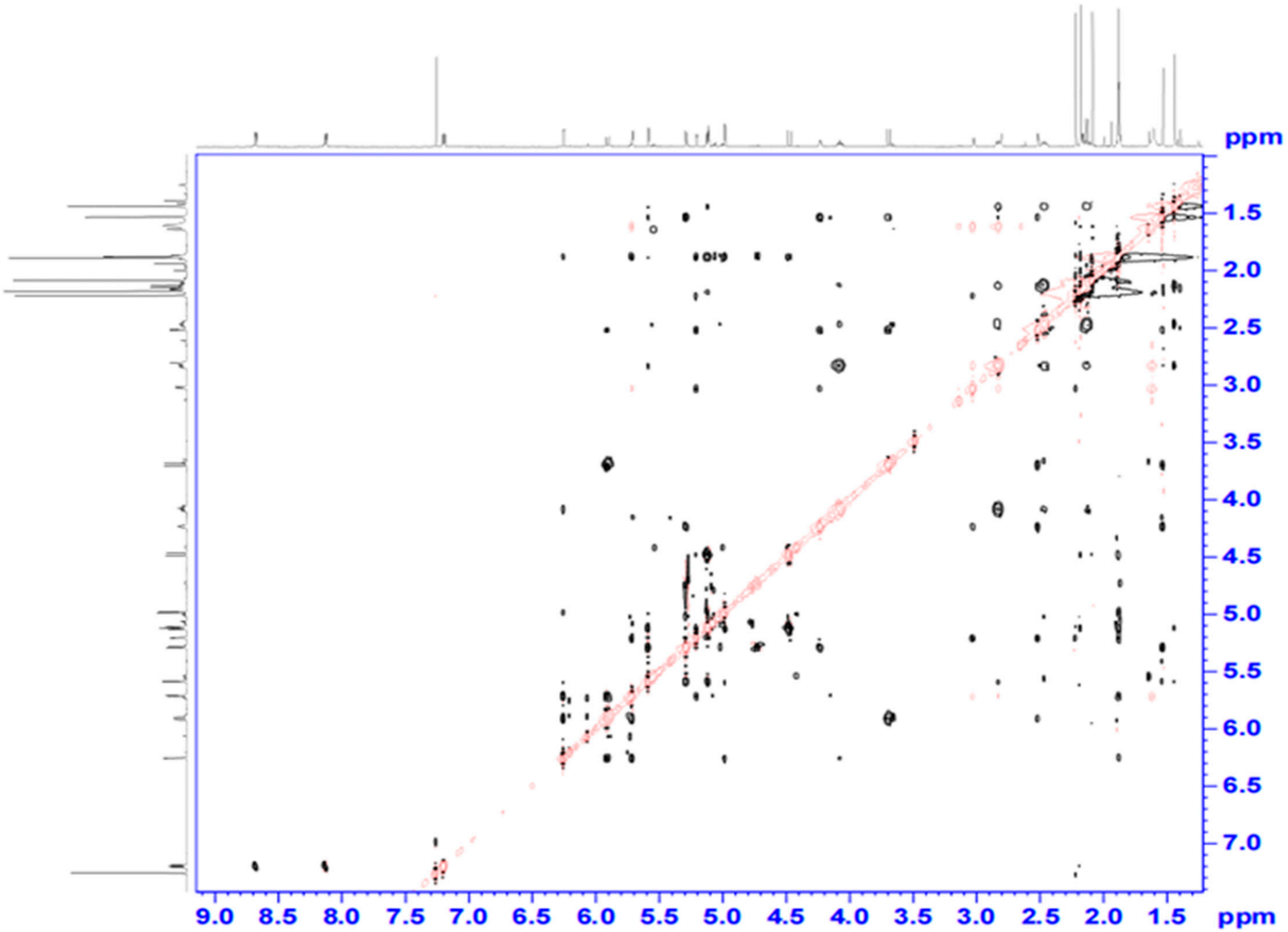

Figure S42. IR spectrum of **4**

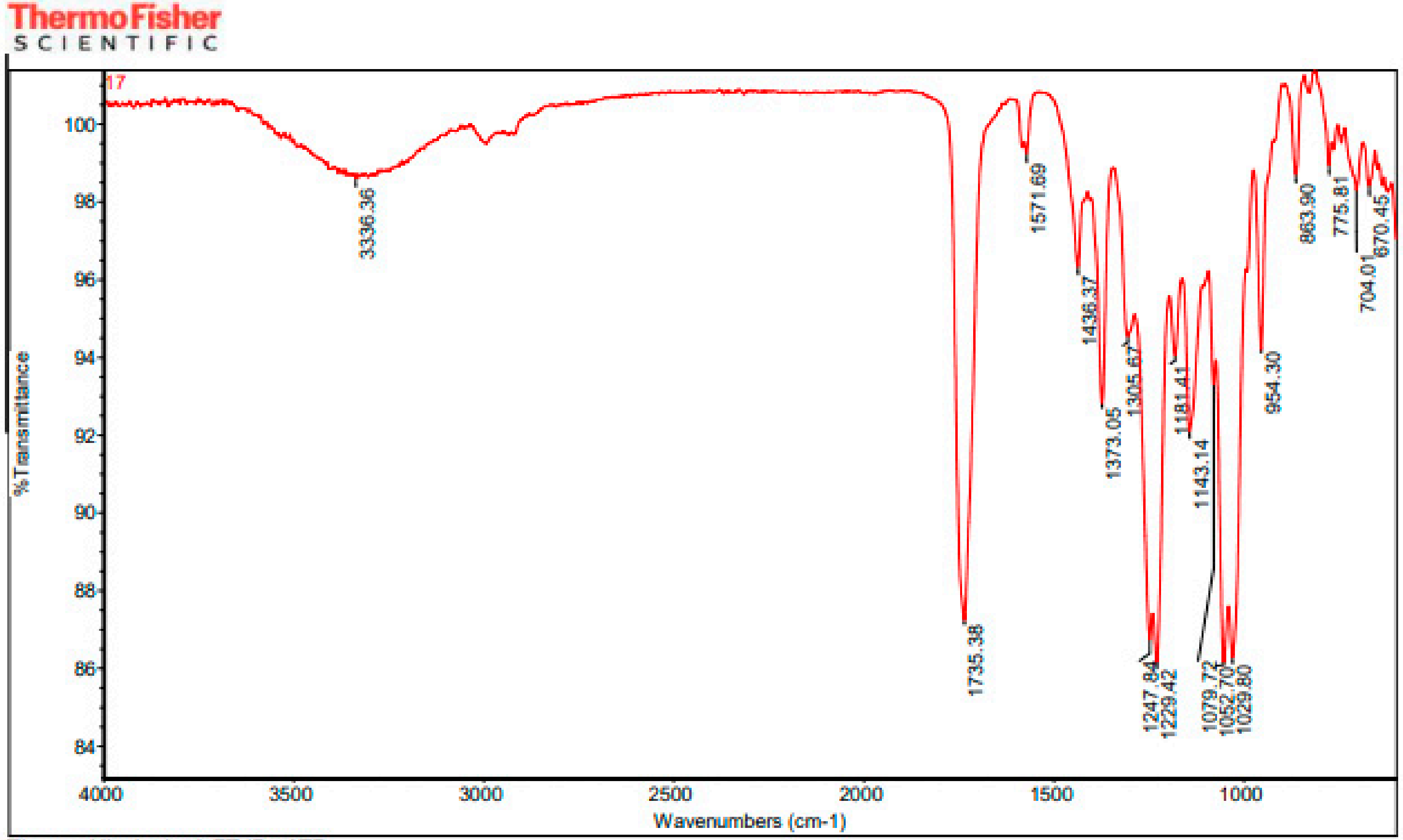

Thermo Nicolet is50 FT-IR ATR
17
Number of sample scans: 16
Number of background scans: 16
Resolution: 4.000
Sample gain: 8.0
Optical velocity: 0.4747
Aperture: 150.00

Figure S43. UV spectrum of **4**

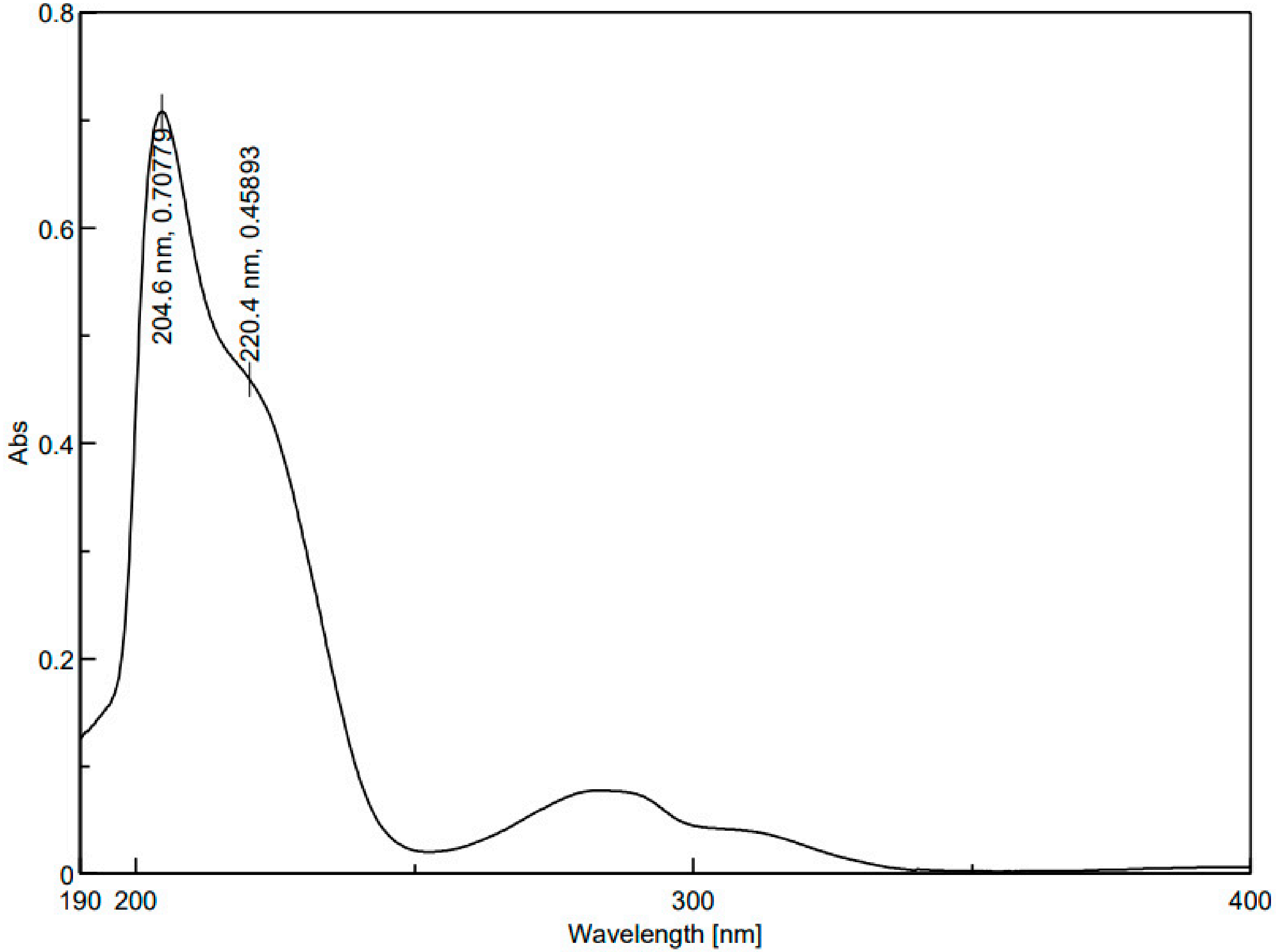

Figure S44. HR-ESI-MS spectrum of compound **4**

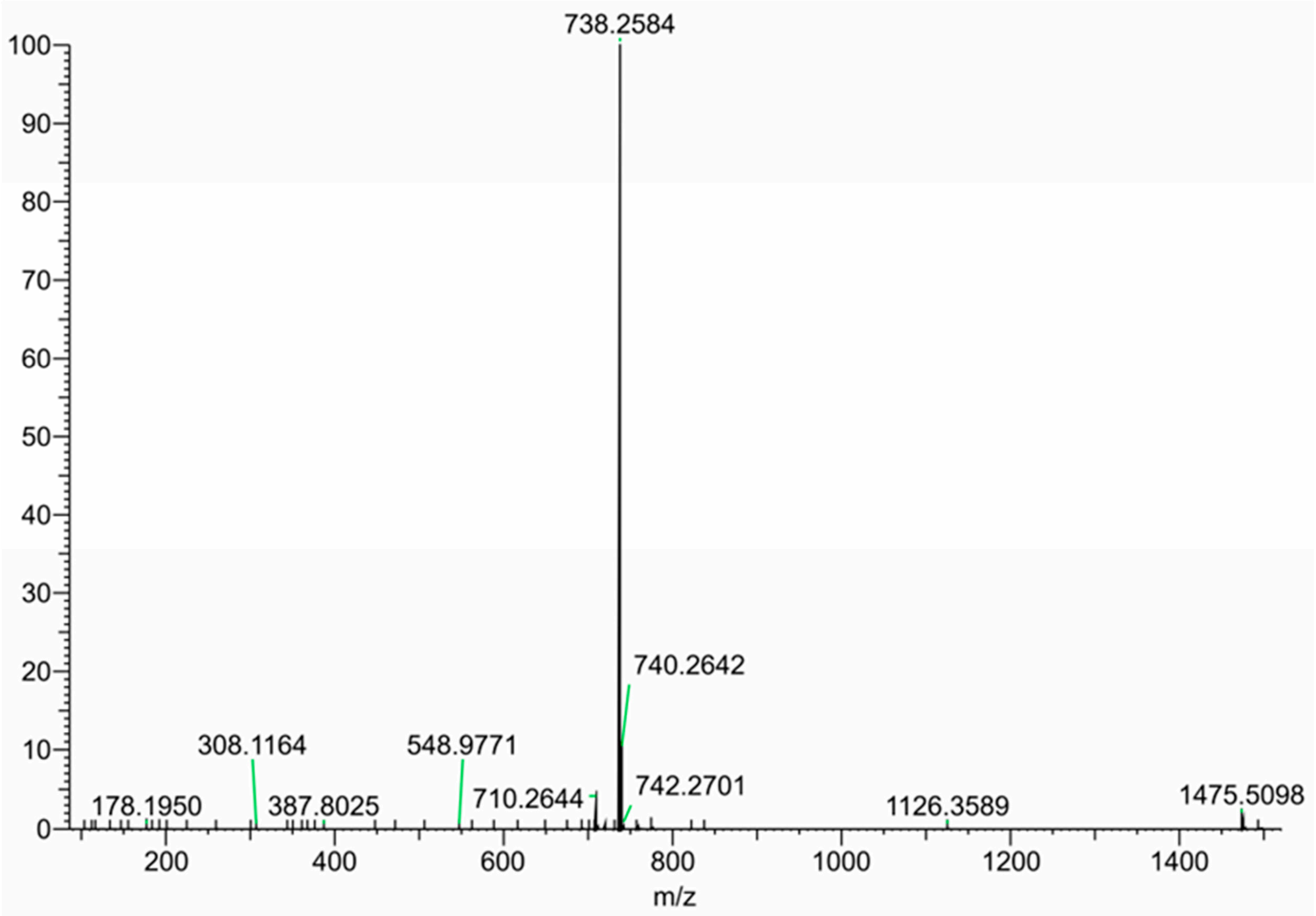

Figure S45. ^{1}H-NMR spectrum of compound **5** ($CDCl_3$, 600 MHz)

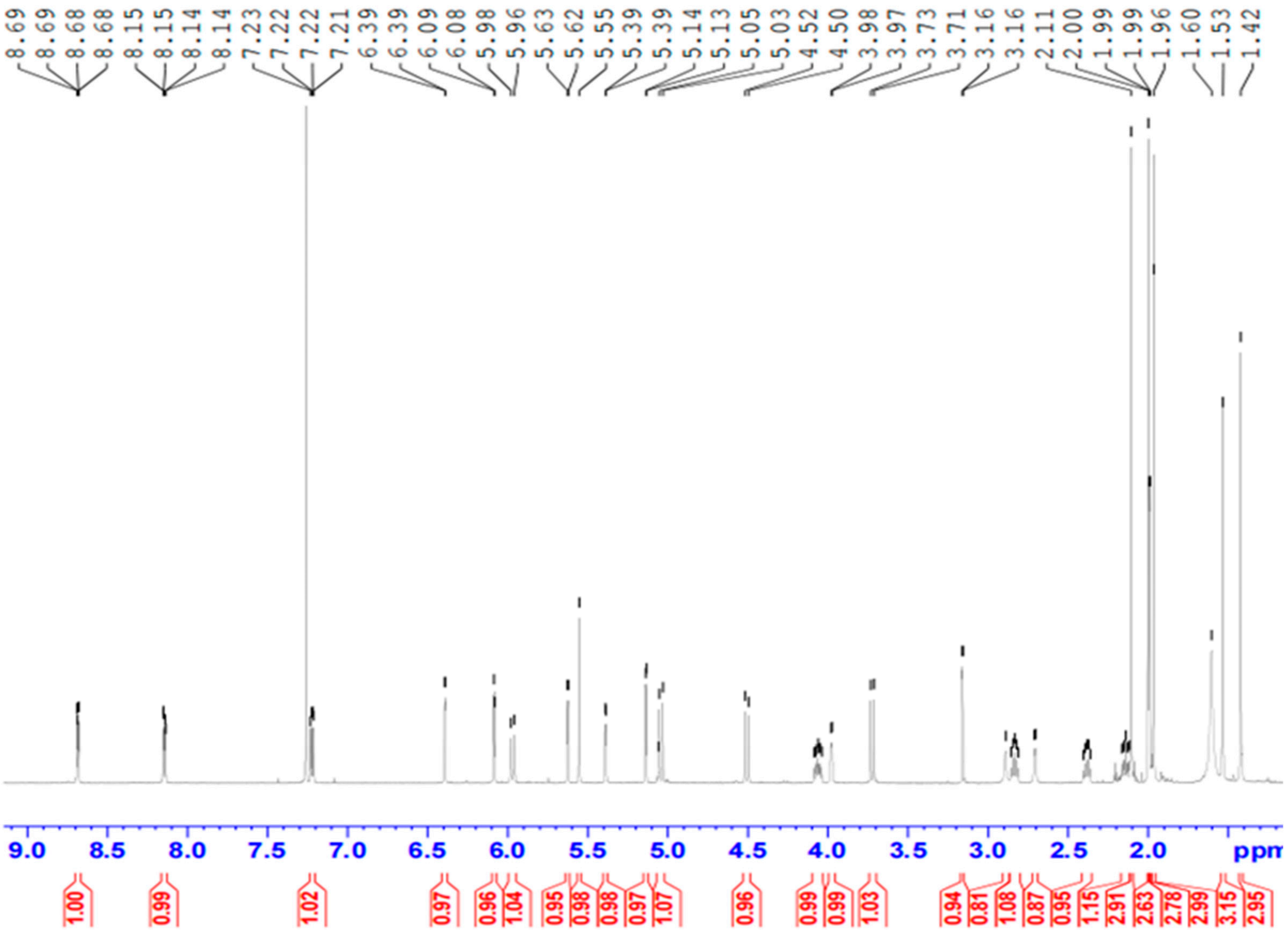

Figure S46. ^{13}C-NMR spectrum of compound **5** ($CDCl_3$, 150 MHz)

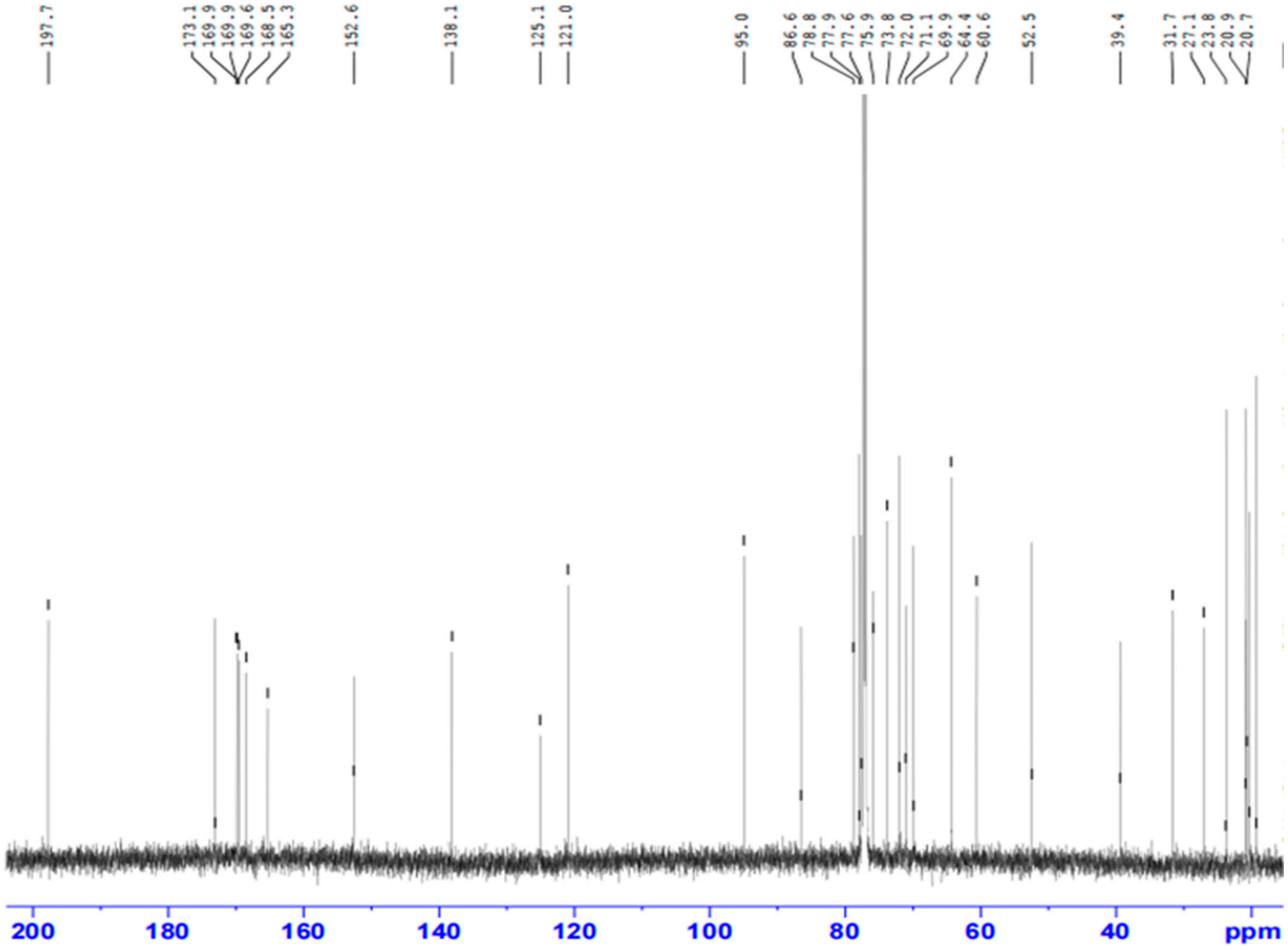

Figure S47. HR-ESI-MS spectrum of compound **5**

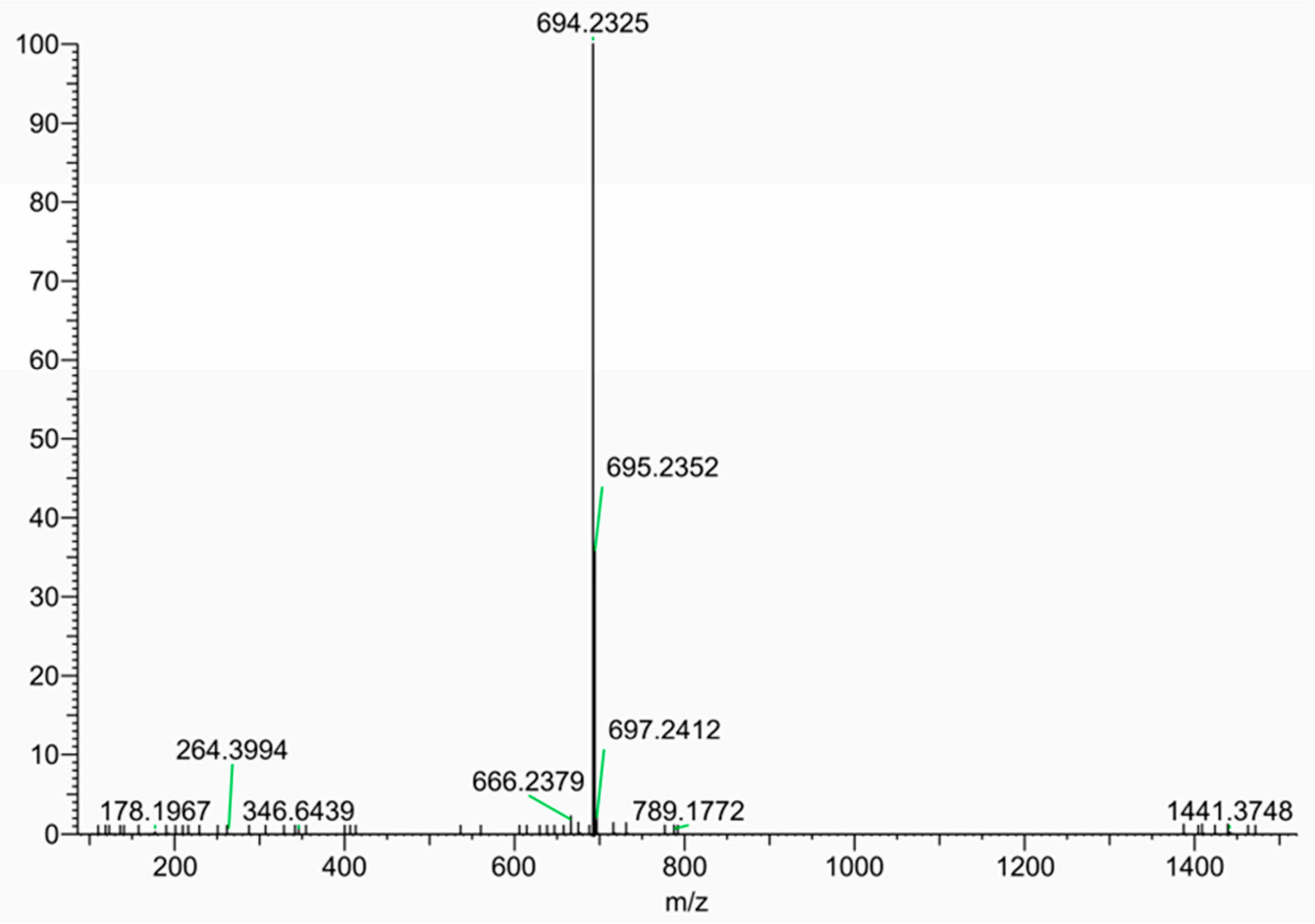

Figure S48. ^{1}H-NMR spectrum of compound **6** ($CDCl_3$, 600 MHz)

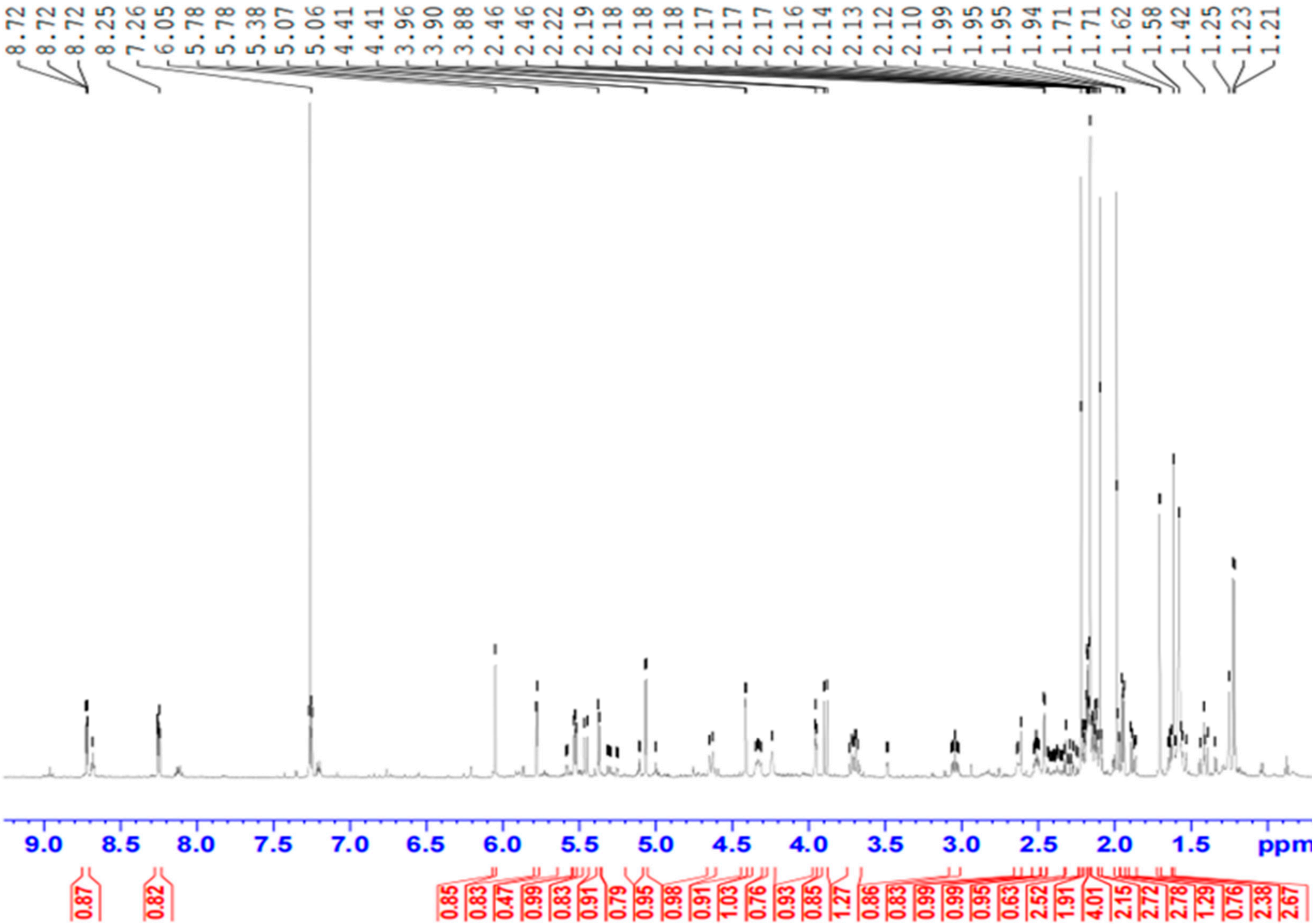

Figure S49. ^{13}C-NMR spectrum of compound **6** ($CDCl_3$, 150 MHz)

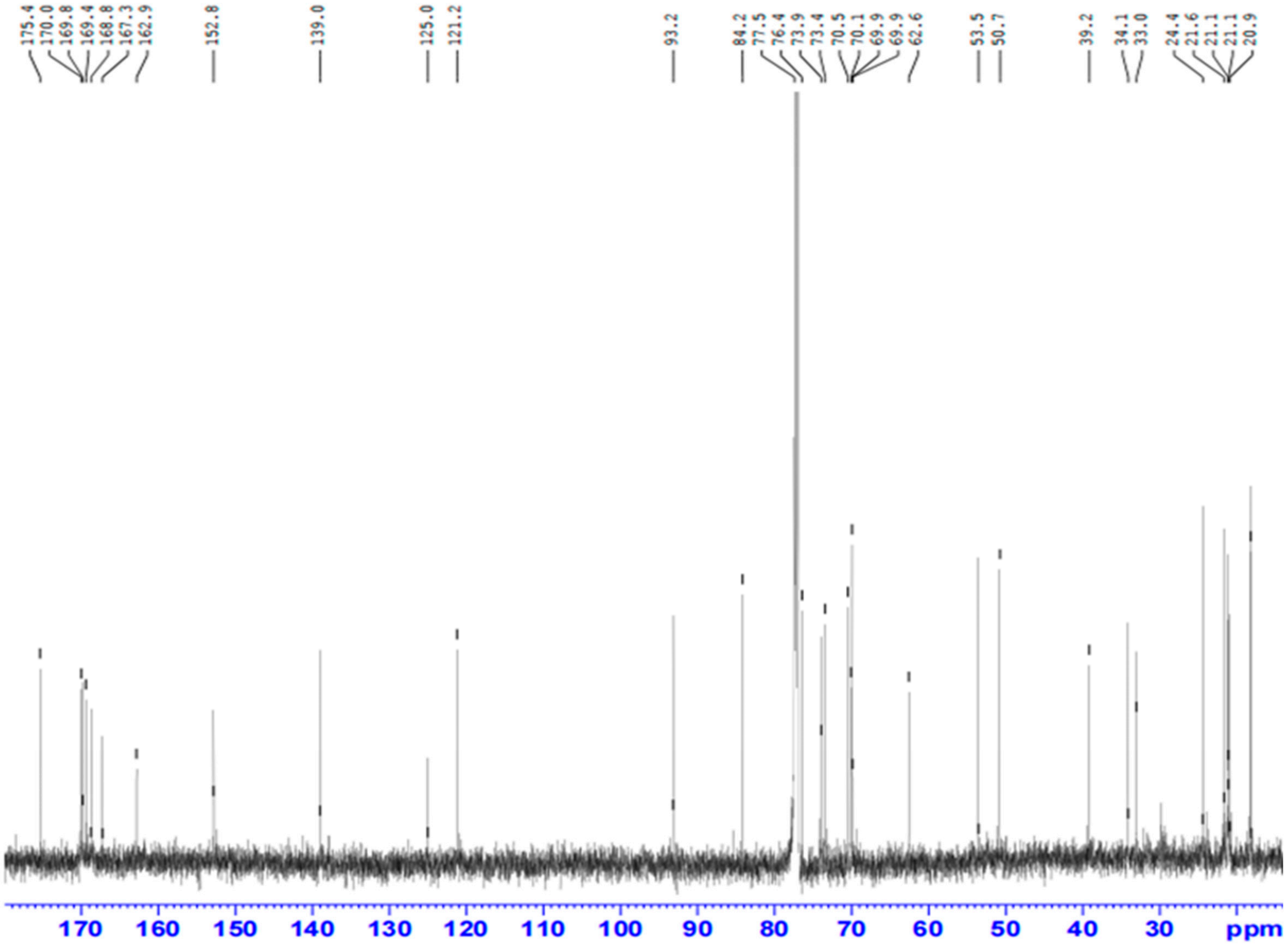

Figure S50. HR-ESI-MS spectrum of compound **6**

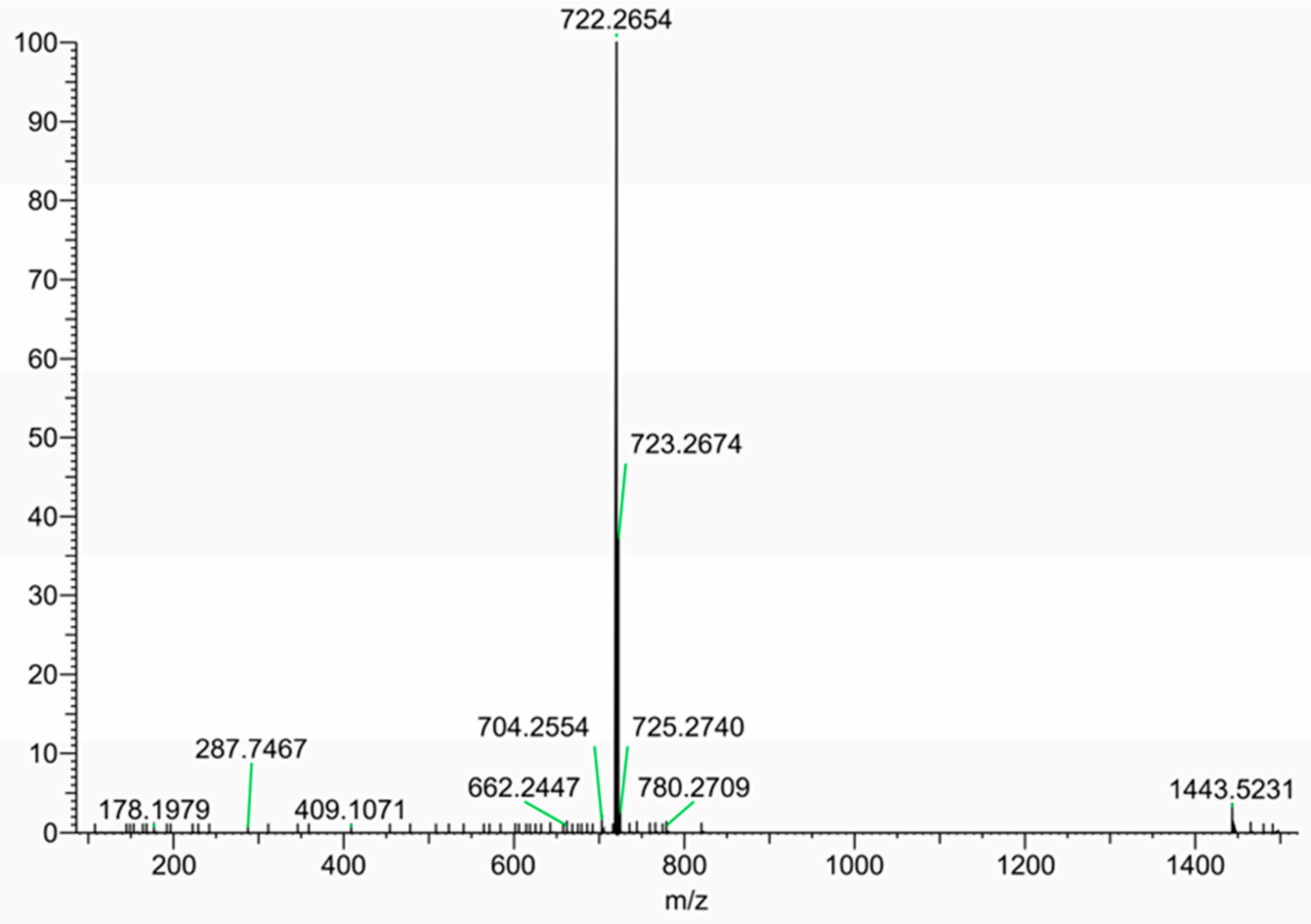

Figure S51. ^{1}H-NMR spectrum of compound **7** ($CDCl_3$, 600 MHz)

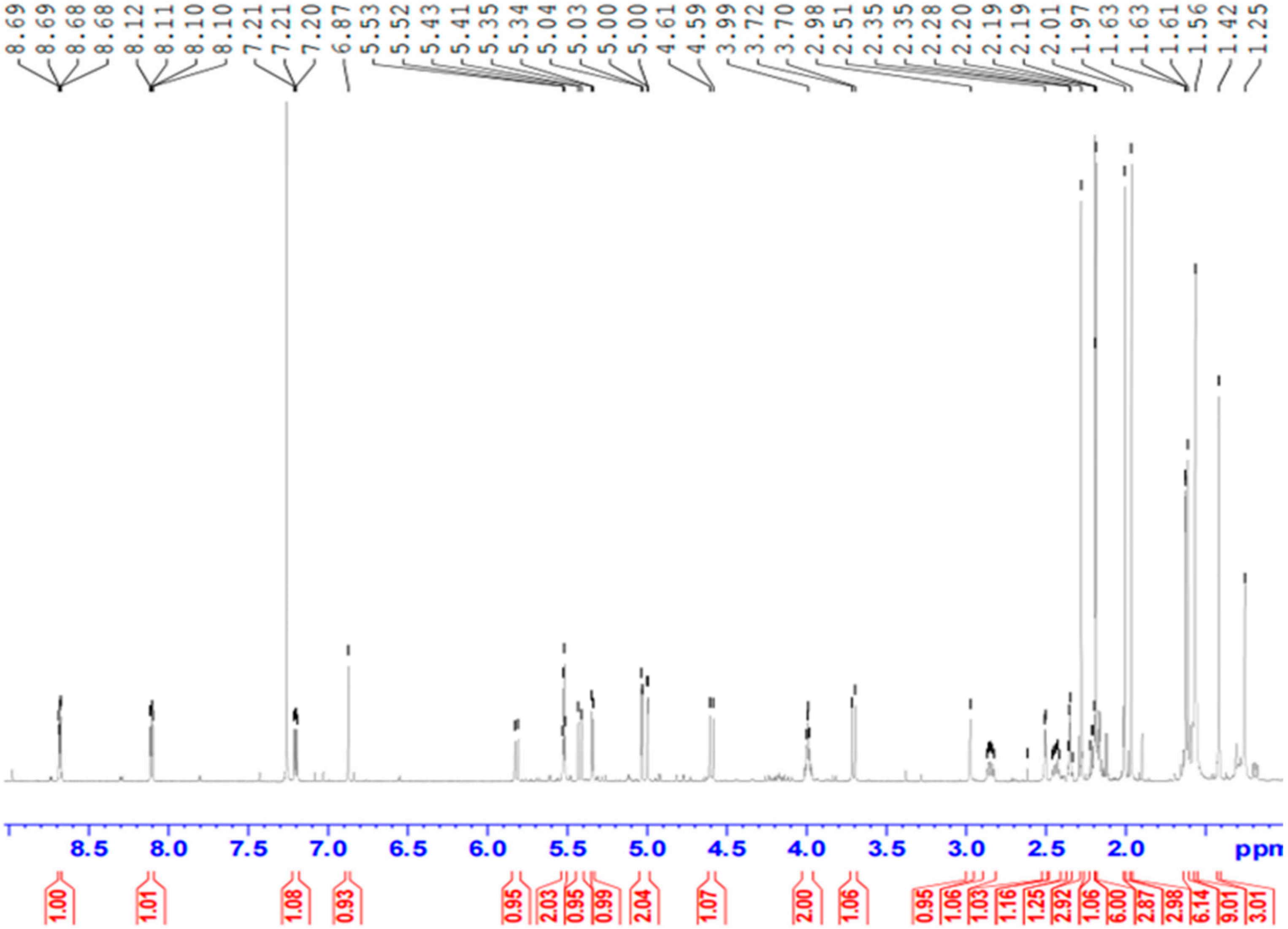

Figure S52. ^{13}C-NMR spectrum of compound **7** ($CDCl_3$, 150 MHz)

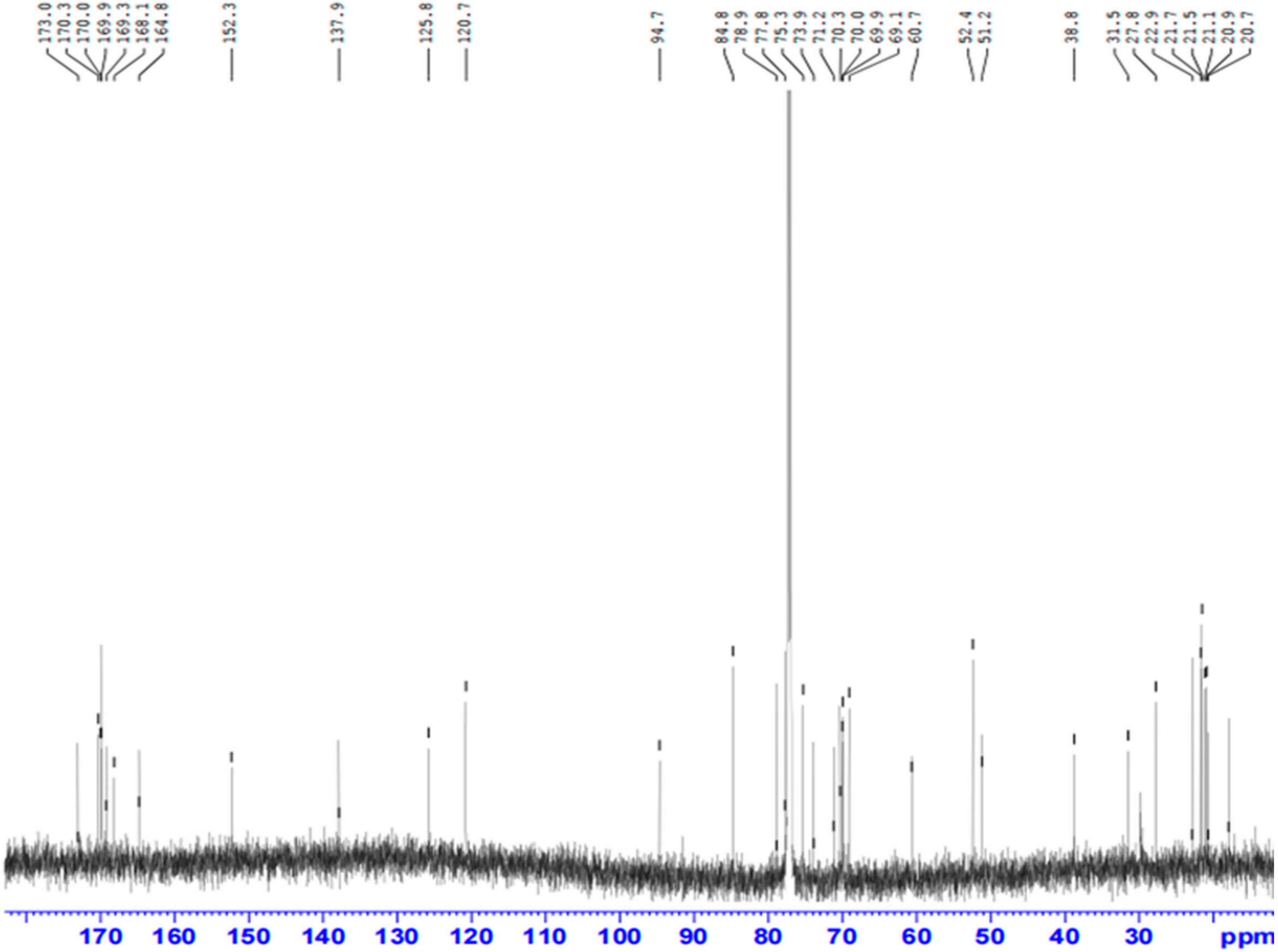

Figure S53. HR-ESI-MS spectrum of compound **7**

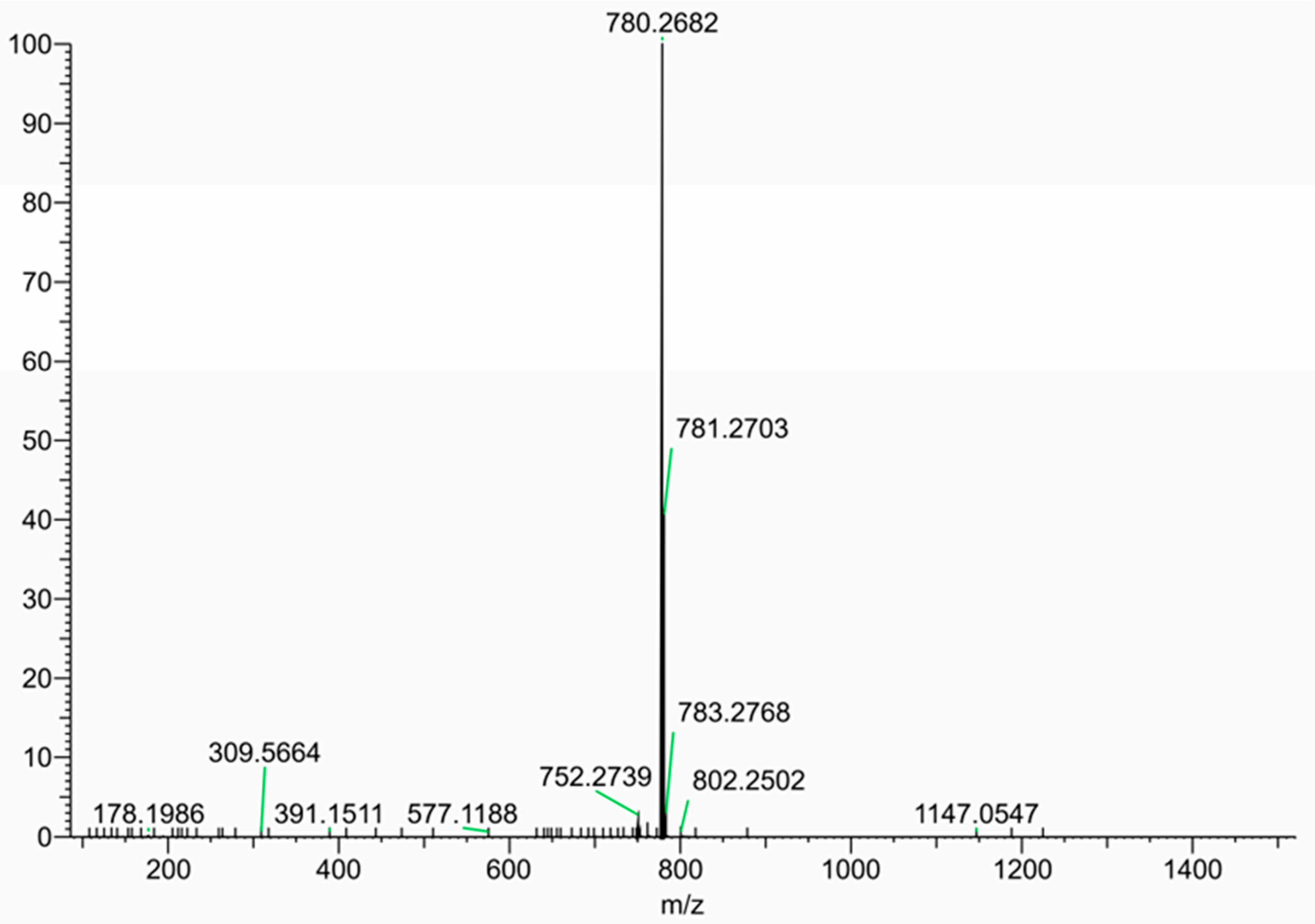

Figure S54. ^{1}H-NMR spectrum of compound **8** ($CDCl_3$, 600 MHz)

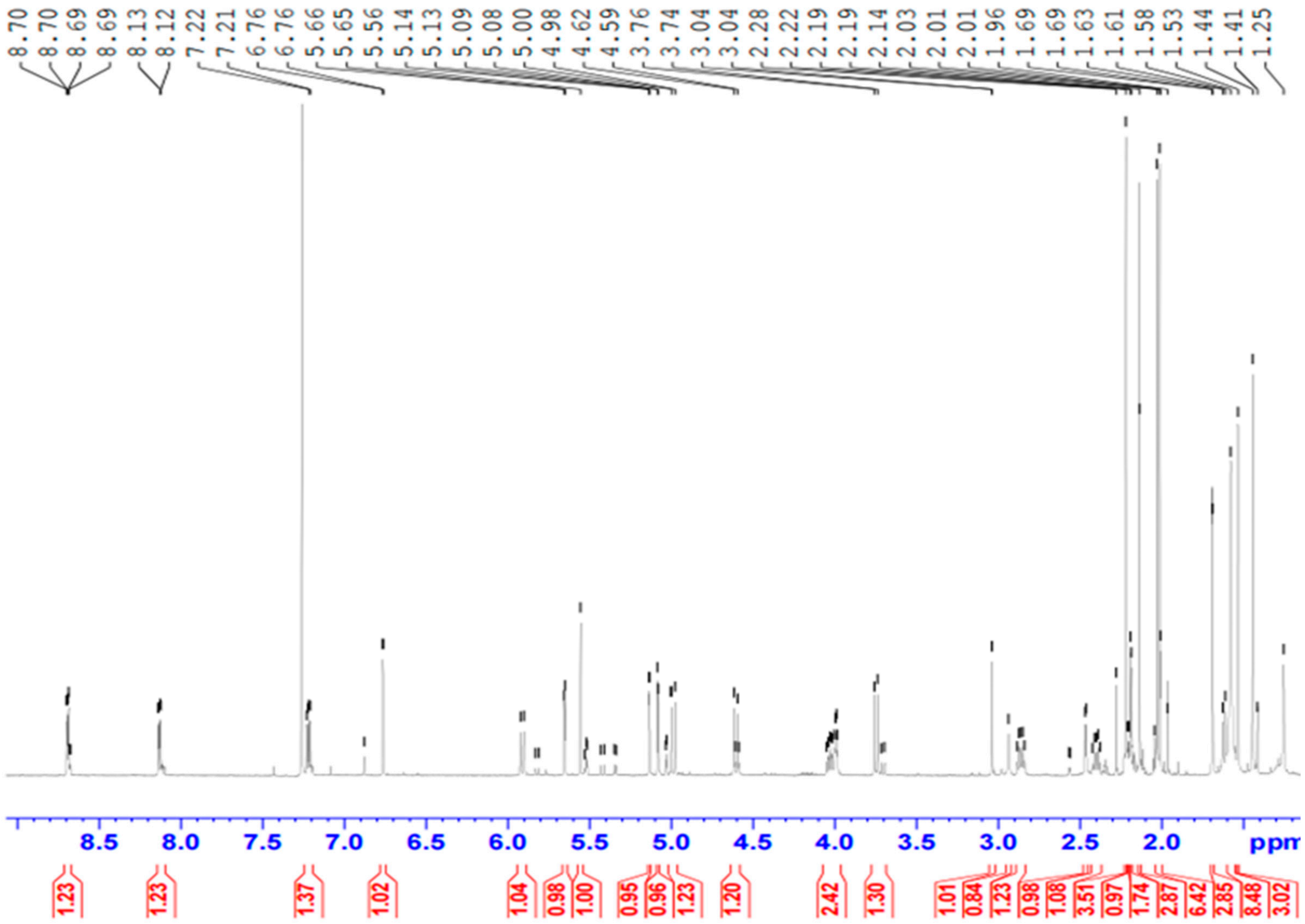

Figure S55. ^{13}C-NMR spectrum of compound **8** ($CDCl_3$, 150 MHz)

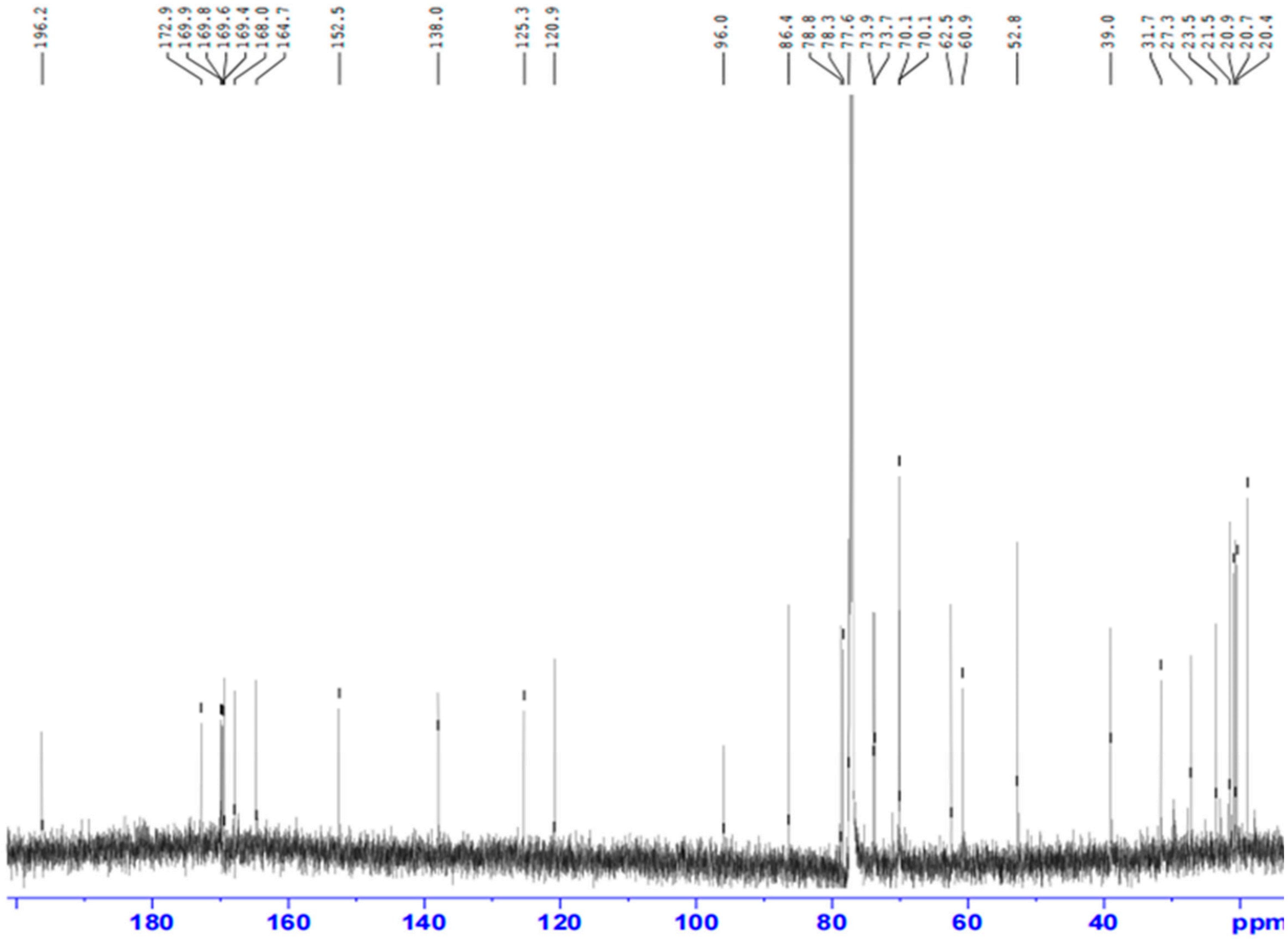

Figure S56. HR-ESI-MS spectrum of compound **8**

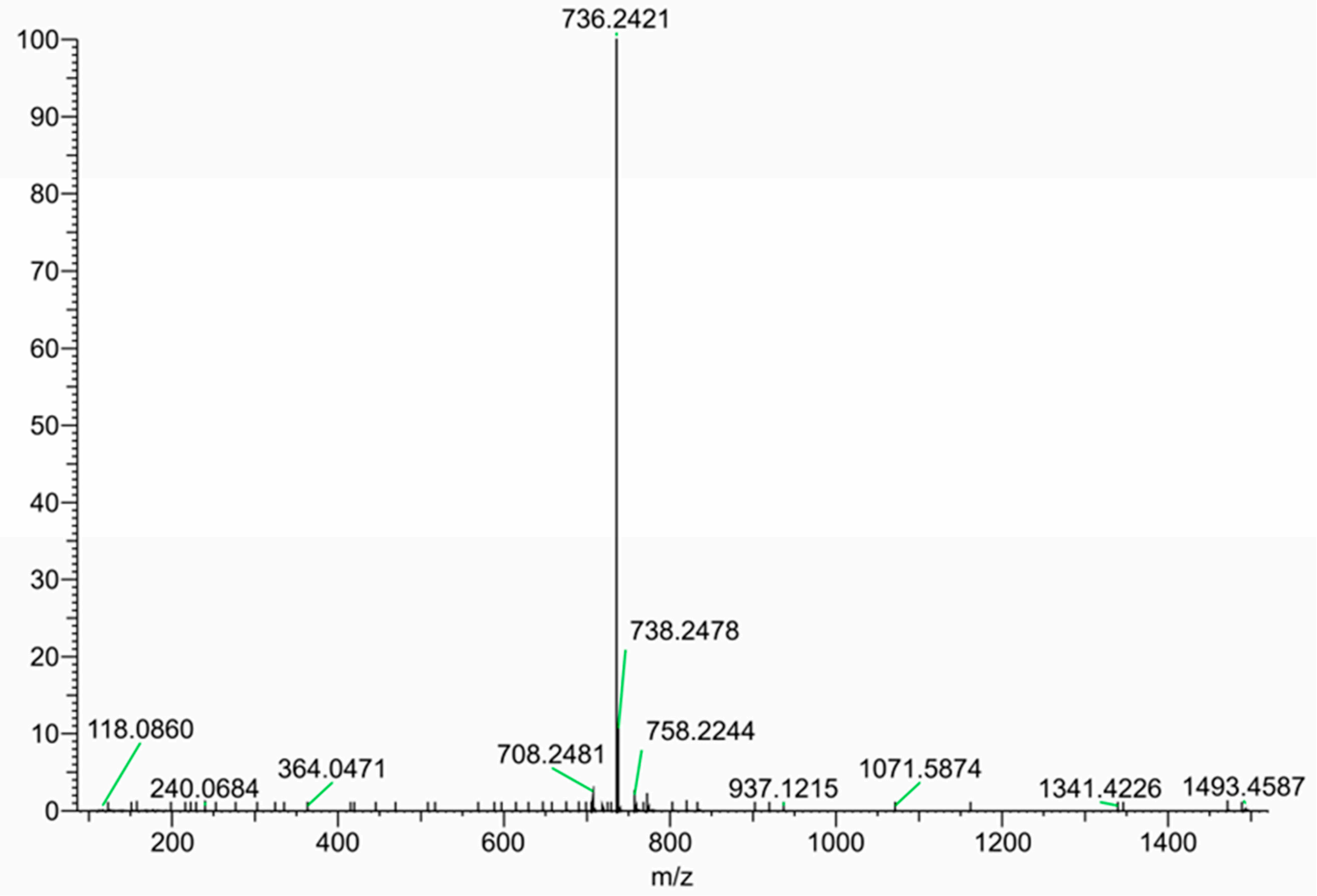

Figure S57. ^{1}H-NMR spectrum of compound **9** ($CDCl_3$, 600 MHz)

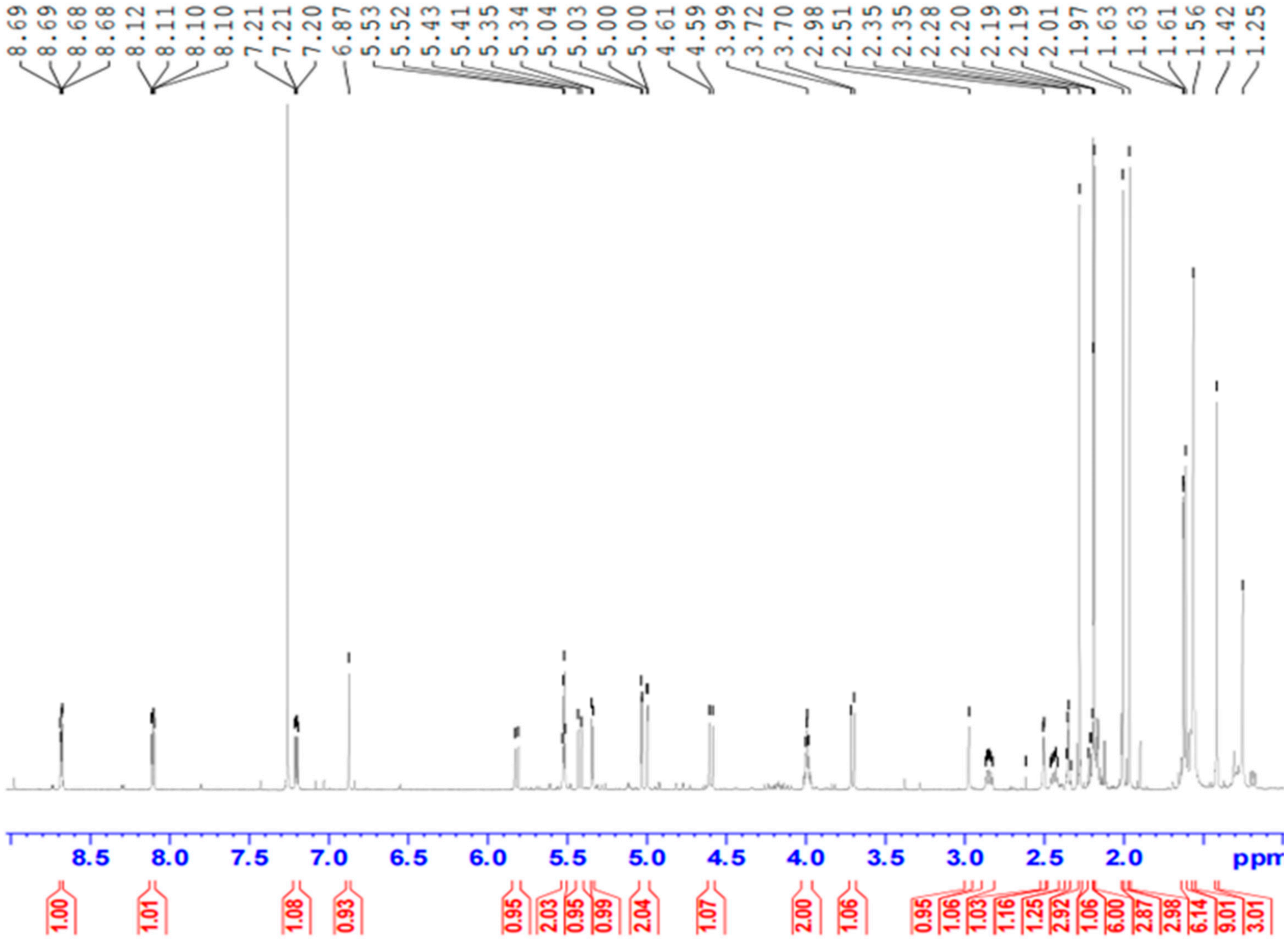

Figure S58. ^{13}C-NMR spectrum of compound **9** ($CDCl_3$, 150 MHz)

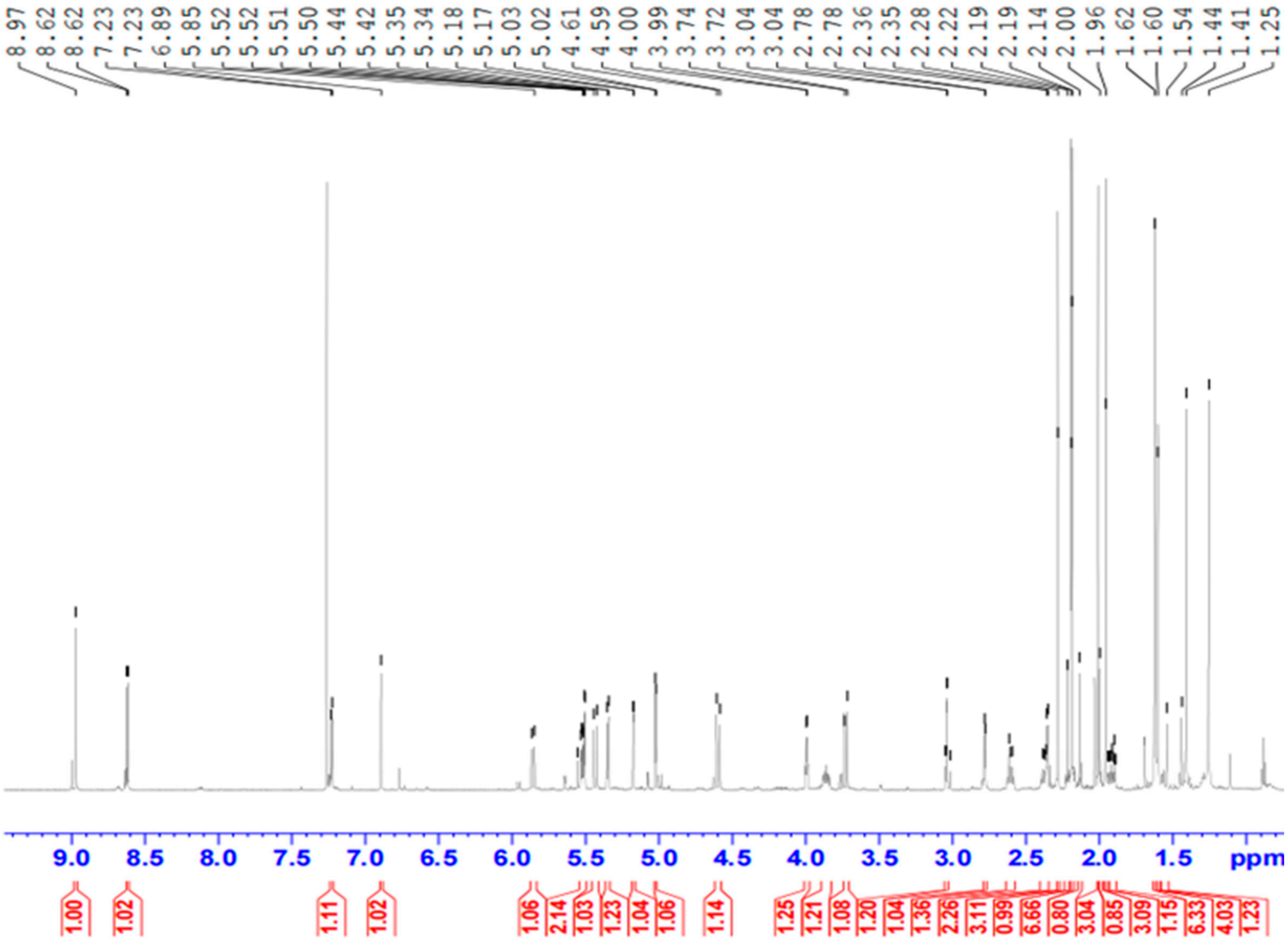

Figure S59. HR-ESI-MS spectrum of compound **9**

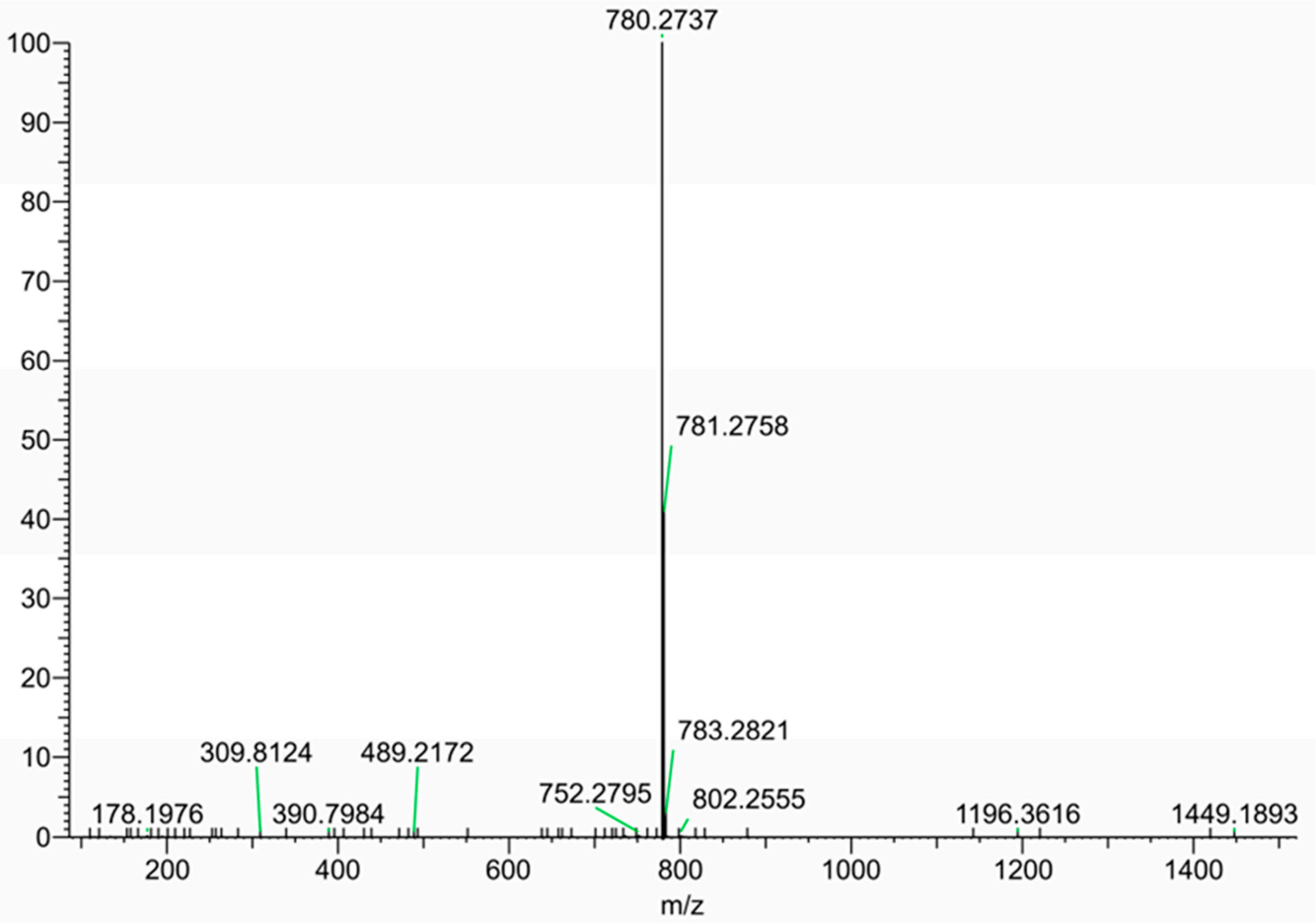

Supplement: Supplementary file 1 [file molecules-31-00271-s001.zip › molecules-4060260-supplementary.pdf]
